# Long-term prognostic impact of paravalvular leakage on coronary artery disease requires patient-specific quantification of hemodynamics

**DOI:** 10.1038/s41598-022-21104-8

**Published:** 2022-12-09

**Authors:** Seyedvahid Khodaei, Louis Garber, Julia Bauer, Ali Emadi, Zahra Keshavarz-Motamed

**Affiliations:** 1 Department of Mechanical Engineering (Mail to JHE-310), McMaster University, Hamilton, ON L8S 4L7 Canada; 2 School of Biomedical Engineering, McMaster University, Hamilton, ON Canada; 3 Department of Electrical and Computer Engineering, McMaster University, Hamilton, ON Canada; 4 School of Computational Science and Engineering, McMaster University, Hamilton, ON Canada

**Keywords:** Biomedical engineering, Engineering, Cardiology, Interventional cardiology

## Abstract

Transcatheter aortic valve replacement (TAVR) is a frequently used minimally invasive intervention for patient with aortic stenosis across a broad risk spectrum. While coronary artery disease (CAD) is present in approximately half of TAVR candidates, correlation of post-TAVR complications such as paravalvular leakage (PVL) or misalignment with CAD are not fully understood. For this purpose, we developed a multiscale computational framework based on a patient-specific lumped-parameter algorithm and a 3-D strongly-coupled fluid–structure interaction model to quantify metrics of global circulatory function, metrics of global cardiac function and local cardiac fluid dynamics in 6 patients. Based on our findings, PVL limits the benefits of TAVR and restricts coronary perfusion due to the lack of sufficient coronary blood flow during diastole phase (e.g., maximum coronary flow rate reduced by 21.73%, 21.43% and 21.43% in the left anterior descending (LAD), left circumflex (LCX) and right coronary artery (RCA) respectively (N = 6)). Moreover, PVL may increase the LV load (e.g., LV load increased by 17.57% (N = 6)) and decrease the coronary wall shear stress (e.g., maximum wall shear stress reduced by 20.62%, 21.92%, 22.28% and 25.66% in the left main coronary artery (LMCA), left anterior descending (LAD), left circumflex (LCX) and right coronary artery (RCA) respectively (N = 6)), which could promote atherosclerosis development through loss of the physiological flow-oriented alignment of endothelial cells. This study demonstrated that a rigorously developed personalized image-based computational framework can provide vital insights into underlying mechanics of TAVR and CAD interactions and assist in treatment planning and patient risk stratification in patients.

## Introduction

Transcatheter aortic valve replacement (TAVR) has become a standard minimally invasive alternative to the traditional surgical aortic valve replacement (SAVR) for patients with aortic valve stenosis (AS) who are at high risk of a complicated surgery. TAVR was recently approved for low-risk patients^1^, indicating that it may become the superior treatment modality for the whole risk spectrum^2^.

However, there are drawbacks to TAVR arising from an improperly placed transcatheter heart valve: (1) *paravalvular leakage* (PVL), a back flow from the aorta to the left ventricle during diastole, is a major complication and an independent predictor of mortality following TAVR. PVL results in increased heart workload and leads to congestive heart failure^3^. It is estimated that 7.8% to 40.8% of PVL post-TAVR is mild, 5% to 37.9% is moderate, and 0.5% to 13.6% is severe^4^; (2) *coronary obstruction*: As the coronary ostia are located superior to the aortic valve, the presence of PVL jets may impede blood flow into the coronary arteries. The PVL most frequently occurs between the left and right coronary cusps (i.e., at the location of native valve commissures: 1 to 2 o’clock of the short axis view)^5^. Accessing one or both coronary arteries is challenging and would require the use of more aggressive methods, such as multiple catheter exchanges, in up to 46% of the patients after first TAVR [4]. The risk of coronary obstruction occurring with TAVR is potentially as high as 23%^6^. A patient suffering with coronary artery obstruction will typically present with severe hypertension and ventricular arrhythmias^7^.

Development, progression, diagnosis, and treatment of cardiovascular disease is closely governed by fluid mechanics^8–10^. Indeed, the correlations between biological fluid mechanics and observed pathological events can be explained on the basis of adverse hemodynamics^9,11^. Detailed analysis of fluid mechanics within the cardiovascular system has led many researchers to conclude that valvular disease depends on the complex hemodynamics of both the ventricle and the vascular system^12–17^.

Interactive coupling of ventricle, valve and vascular systems should be taken into consideration for accurate quantitative evaluation of hemodynamics in patients who receive TAVR to quantify **global** flow environment (metrics of cardiac function and circulatory function, e.g., heart workload and its contribution breakdown of each component of the cardiovascular diseases) and the **local** microenvironment of flowing blood (coronary and valve fluid dynamics, e.g., details of the instantaneous 3-D flow)^12–16^. Despite the importance and advances in medical imaging, the current clinical diagnostic tools cannot sufficiently quantify flow conditions in patients with many cardiovascular diseases, including  patients with valvular diseases who undergo TAVR^18–21^. More specifically, several imaging modalities exist for the coronary arteries, such as angiography, computed tomography coronary angiography (CTCA), cardiac magnetic resonance (CMR), echocardiography, ultrafast ultrasound, intravascular ultrasound (IVUS), and optical coherence tomography (OCT). However, all modalities, with the exception of CMR, are unable to quantify local and global hemodynamics non-invasively^22,23^. CMR can only quantify local hemodynamics but has been limited to patients without a pacemaker, with the exception of MRI-compatible pacemakers^22^. In addition, coronary imaging with MRI is not routinely performed in clinical practice due to its limited spatial resolution^24^.

In this study, the effect of TAVR complications such as PVL and misalignments on the coronary arteries and aortic root were assessed using patient-specific lumped-parameter algorithm and a 3-D fluid-structure interactions (FSI) model (Figs. 1, 2, 3, 4, 5, 6, 7, 8) to quantify the global and local hemodynamics in 6 patients with severe AS who underwent TAVR (Figs. 9, 10, 11, 12, 13, 14, 15, 16, 17). The proposed framework could provide a platform for testing the intervention scenarios and evaluating their effects on the hemodynamics.

**Figure 1:**
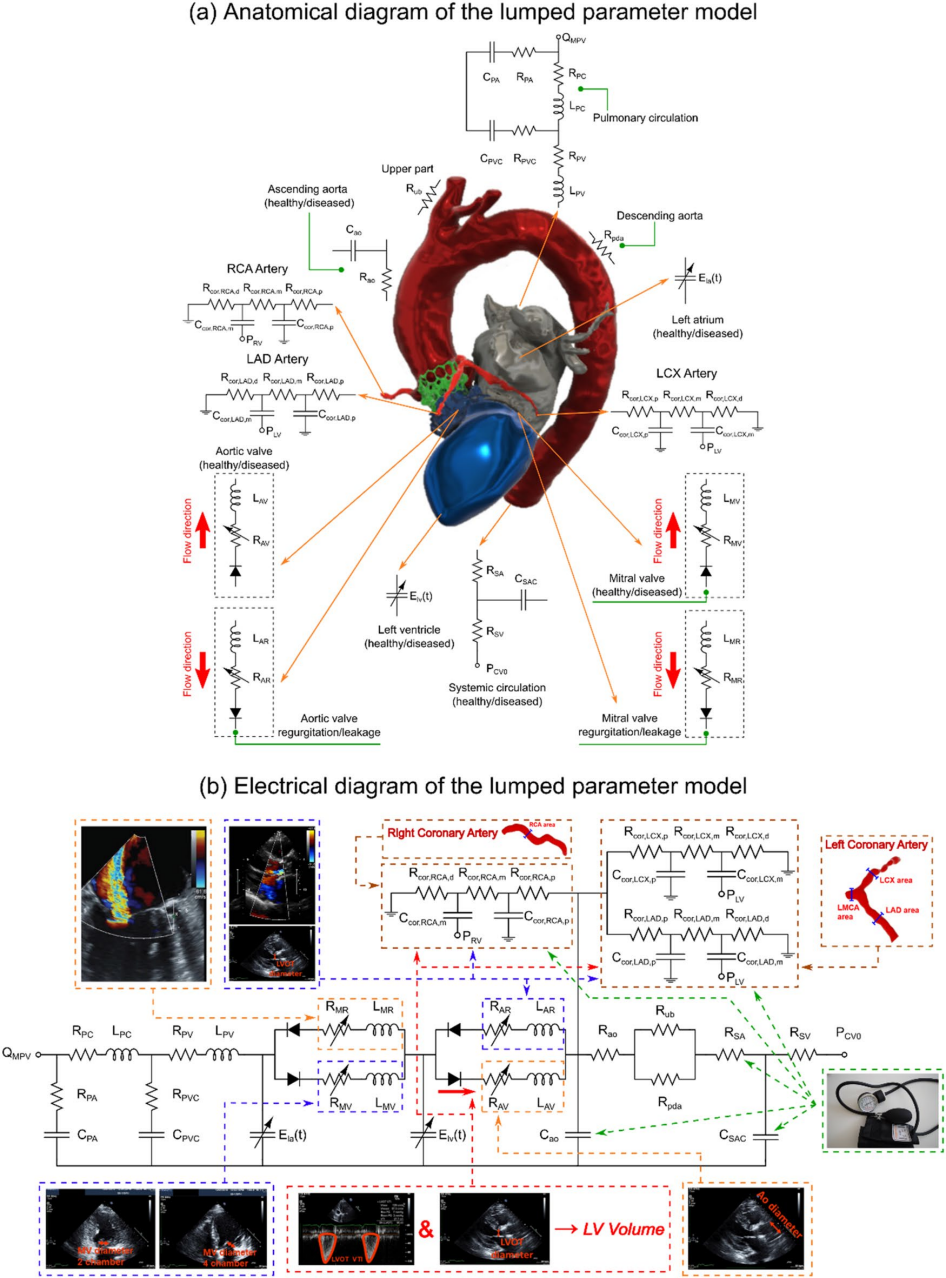
Electrical and anatomical schematic diagrams of the lumped parameter modeling. (**a**) Anatomical illustration; (**b**) Electrical depiction. This model includes the following sub-models: left main coronary artery, left anterior descending coronary artery, left circumflex coronary artery and right coronary artery, left ventricle, aortic valve, left atrium, mitral valve, aortic valve regurgitation, mitral valve regurgitation, systemic circulation, pulmonary circulation. Abbreviations are the same as in Table 2.

**Figure 2:**
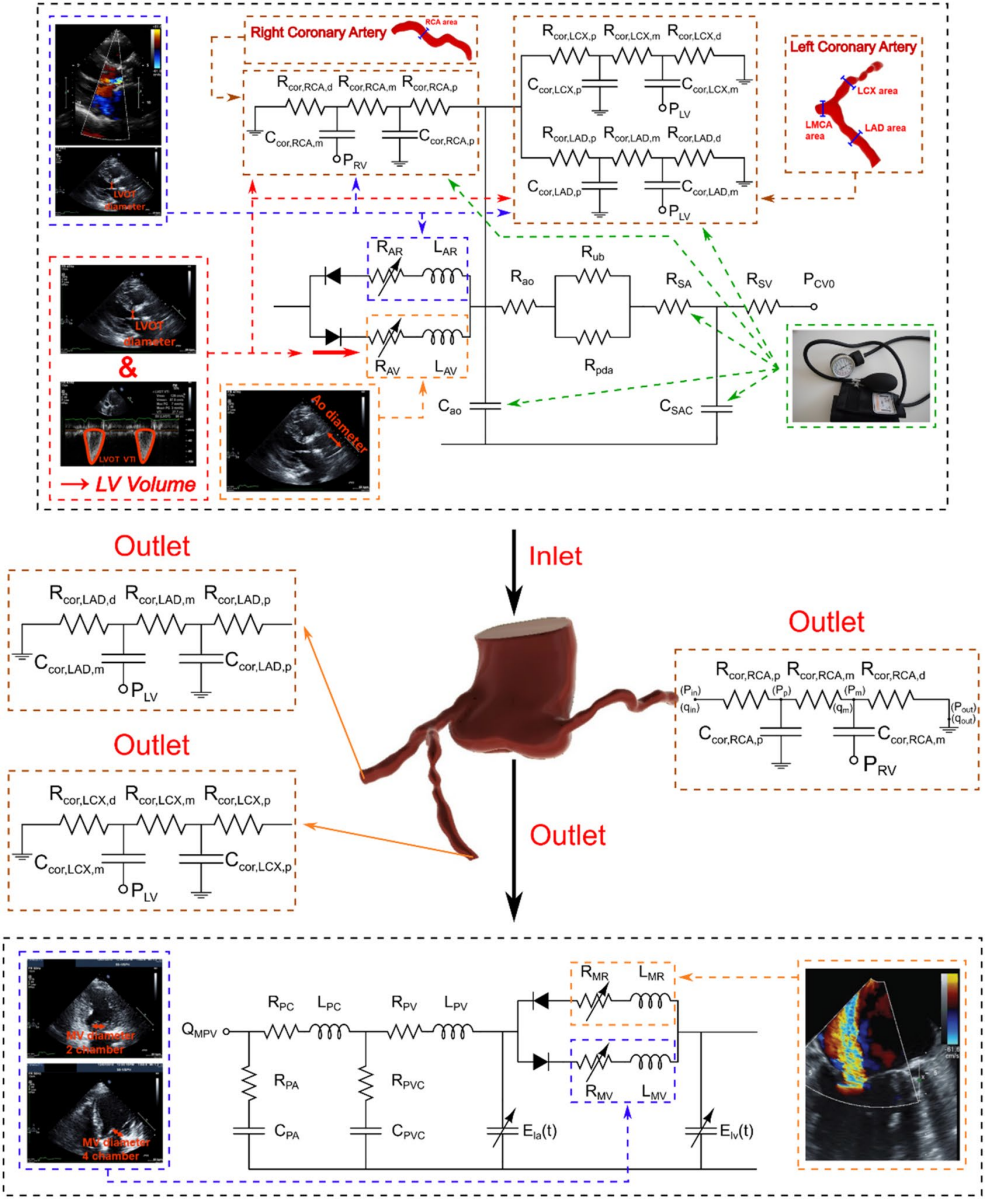
Schematic diagram of computational domain. Electrical and anatomical schematic diagrams of the patient-specific lumped parameter model that provides patient-specific boundary conditions for the fluid domain. This model includes the following sub-models. (1) ascending aorta, (2) left ventricle, (3) left anterior descending coronary artery, (4) left circumflex coronary artery, and (5) right coronary artery. Abbreviations are the same as in Table 2. *Simulation domain and FSI modeling*. Patient-specific LPM simulating the function of the left side of the heart and coronary arteries provided the patient-specific boundary conditions of the inlet and outlets.

**Figure 3:**
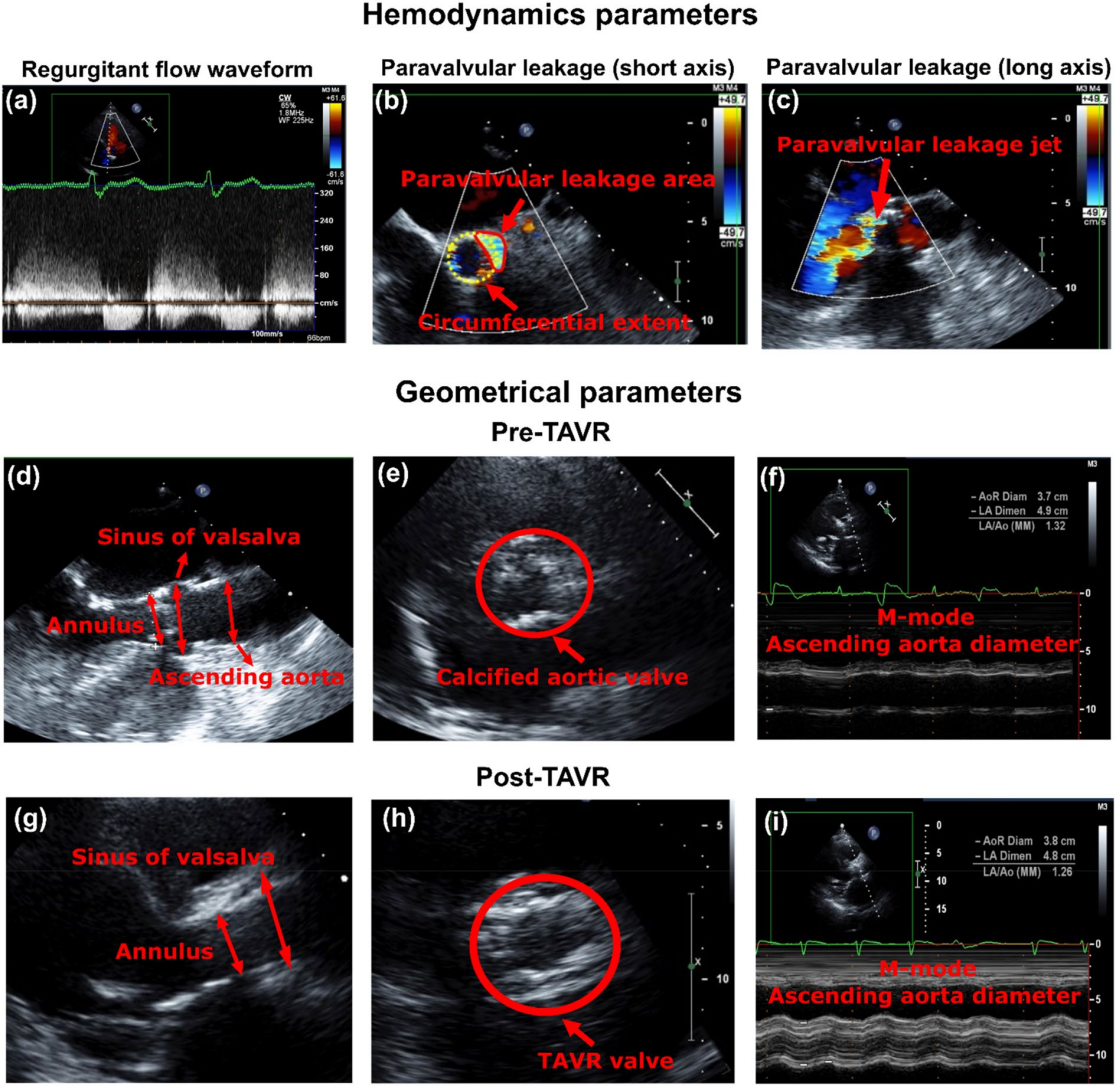
Hemodynamics parameter: (**a**) Regurgitant flow velocity waveform at the paravalvular leakage cite (Apical 5 chamber view); (**b**) Parasternal short-axis color doppler view of the prosthetic valve and the paravalvular leakage area (Vena contracta area: 1.04 cm^2^) and its circumferential extent with respect to prosthetic valve diameter (35%); (**c**) Long axis color doppler view of paravalvular leakage jet interaction with diastolic flow behind the posterior mitral valve leaflet. Geometrical parameters: (**d**) Parasternal long-axis view associated with different parts of the aortic root and ascending aorta before TAVR; (**e**) Parasternal short-axis view of aortic valve before TAVR; (**f**) M-Mode measurement of ascending aorta before TAVR; (**g**) Parasternal long-axis view associated with different parts of the aortic root, prosthetic frame and ascending aorta after TAVR; (**h**) Parasternal short-axis view of TAVR; (**i**) M-Mode measurement of ascending aorta after TAVR.

**Figure 4:**
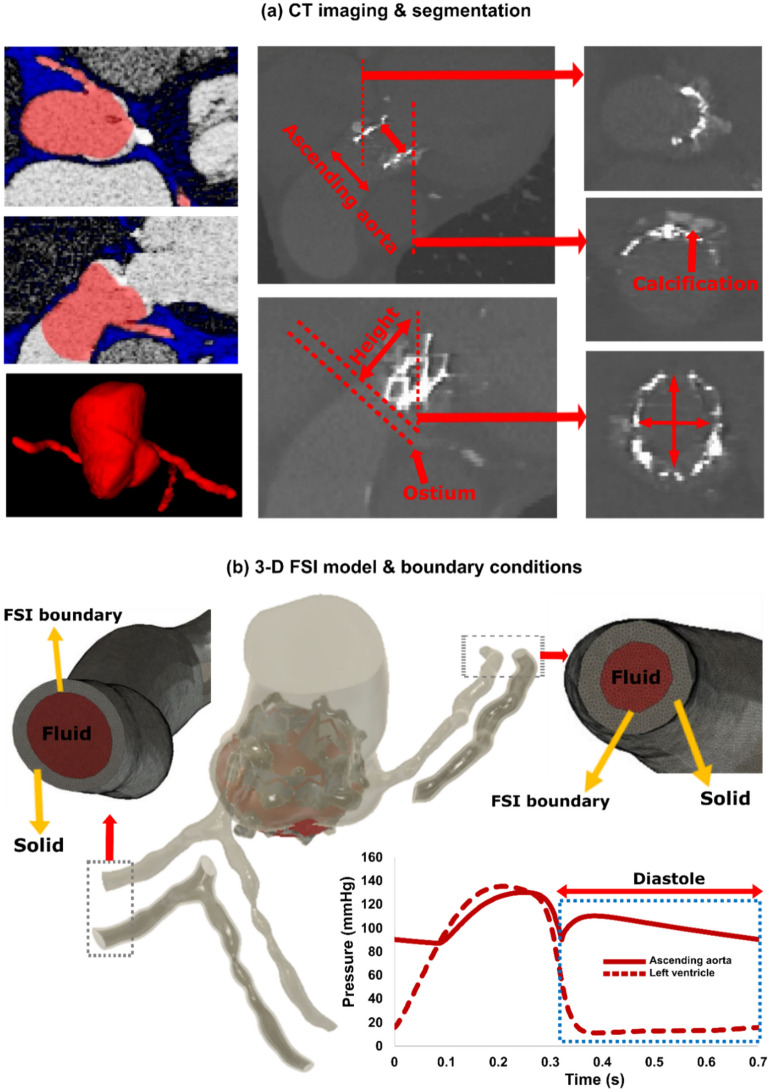
Reconstructed 3-dimensional geometry in a patient with AS who received TAVR using computed tomography, schematic diagram of LPM pressure boundary conditions and FSI. (**a**) CT views (coronal, sagittal and axial) of the ascending aorta, coronary branches, sinus of Valsalva and aortic valve for pre-TAVR (left column) and post-TAVR (middle and right column) as well as the segmentation process and geometry reconstruction; (**b**) computational domain and boundary condition. FSI simulations performed during diastole. Patient-specific LPM simulating the coronary perfusion pressure (ascending aorta pressure—LV diastolic pressure). This data was obtained from patient-specific imaged based lumped parameter model (Fig. 1).

**Figure 5:**
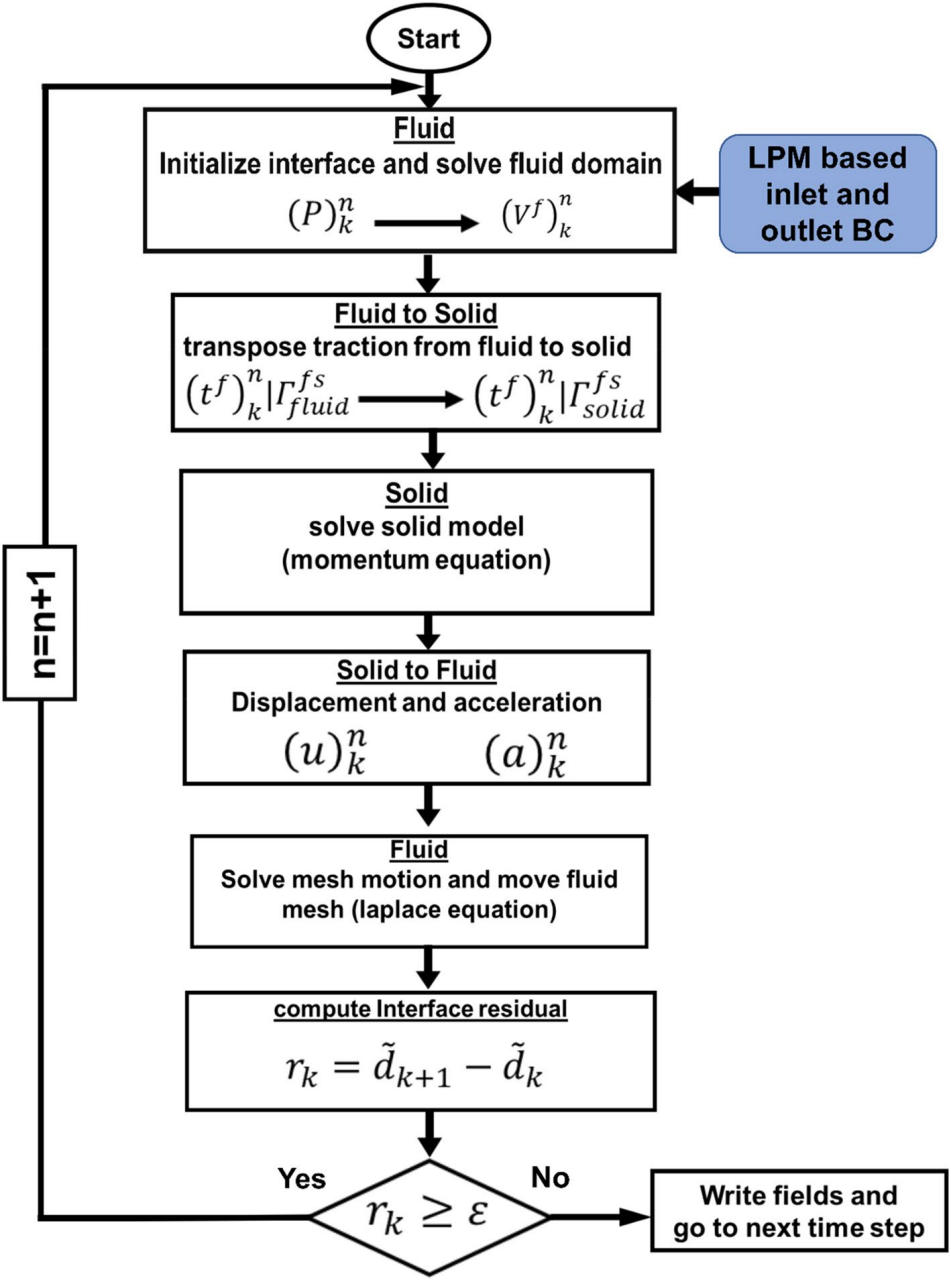
Patient-specific lumped parameter boundary conditions (BC) and strongly coupled FSI model flow chart.

**Figure 6:**
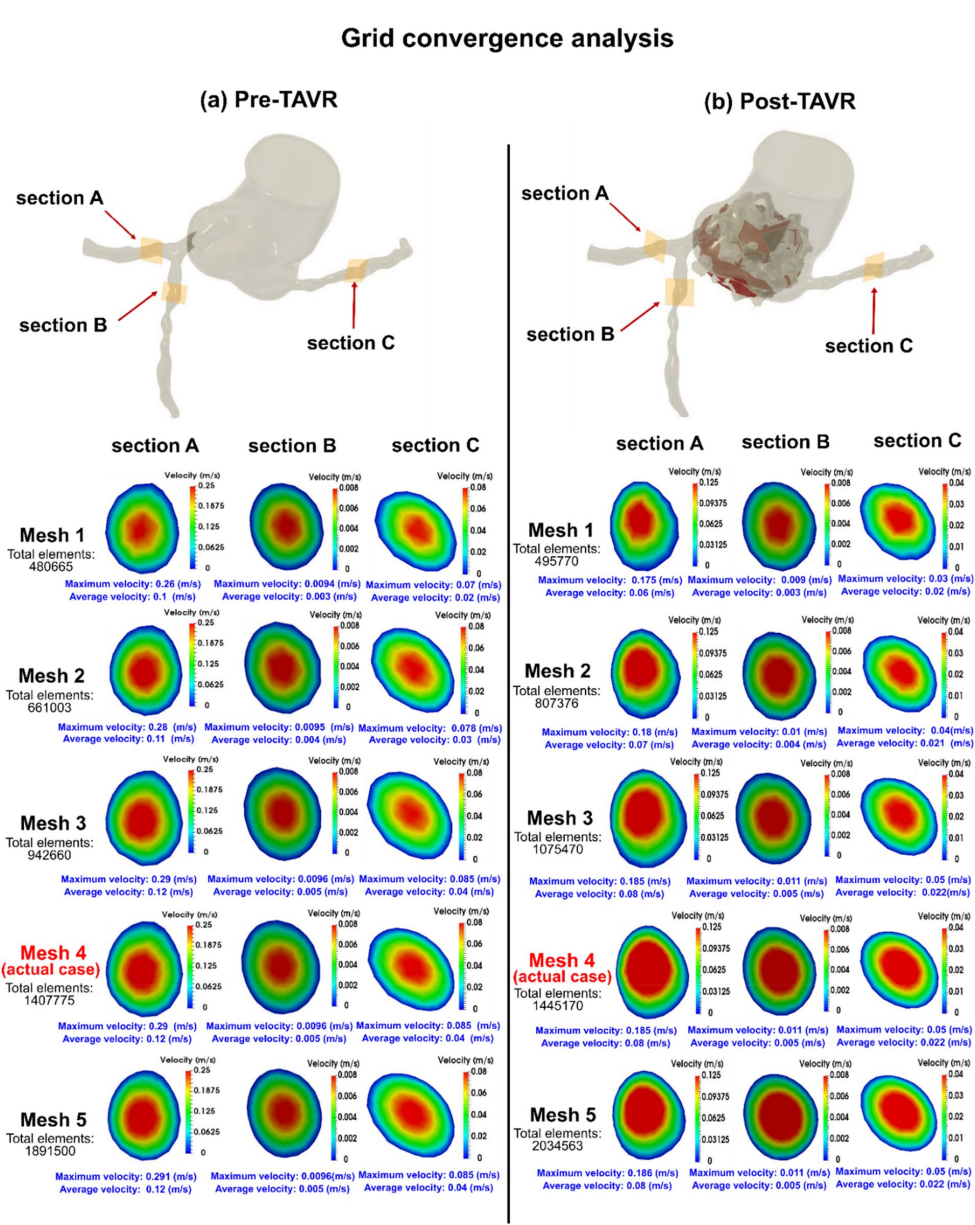
Grid convergence analysis. (**a**) Pre-TAVR: velocity comparisons for different mesh resolutions at the peak of filling phase for different coronary cross sections (sections A,B and C): difference in average velocity between mesh#3 and mesh#4 is less than 0.3% and between mesh#4 and #5 is less than 0.2%—difference in maximum velocity between mesh#3 and mesh#4 is less than 0.5% and between mesh#4 and mesh#5 is less than 0.3%; (**b**) Post-TAVR: velocity comparisons for different mesh resolutions at the peak of filling phase for different coronary cross sections (sections A,B and C): difference in average velocity between mesh#3 and mesh#4 is less than 0.3% and between mesh#4 and #5 is less than 0.2%—difference in maximum velocity between mesh#3 and mesh#4 is less than 0.5% and between mesh#4 and mesh#5 is less than 0.3%; In the aortic root and coronary arteries, the blood flow is laminar and does not experience turbulence during the diastolic phase. In this study, for all 3 patients, we considered the blood flow to be laminar.

**Figure 7:**
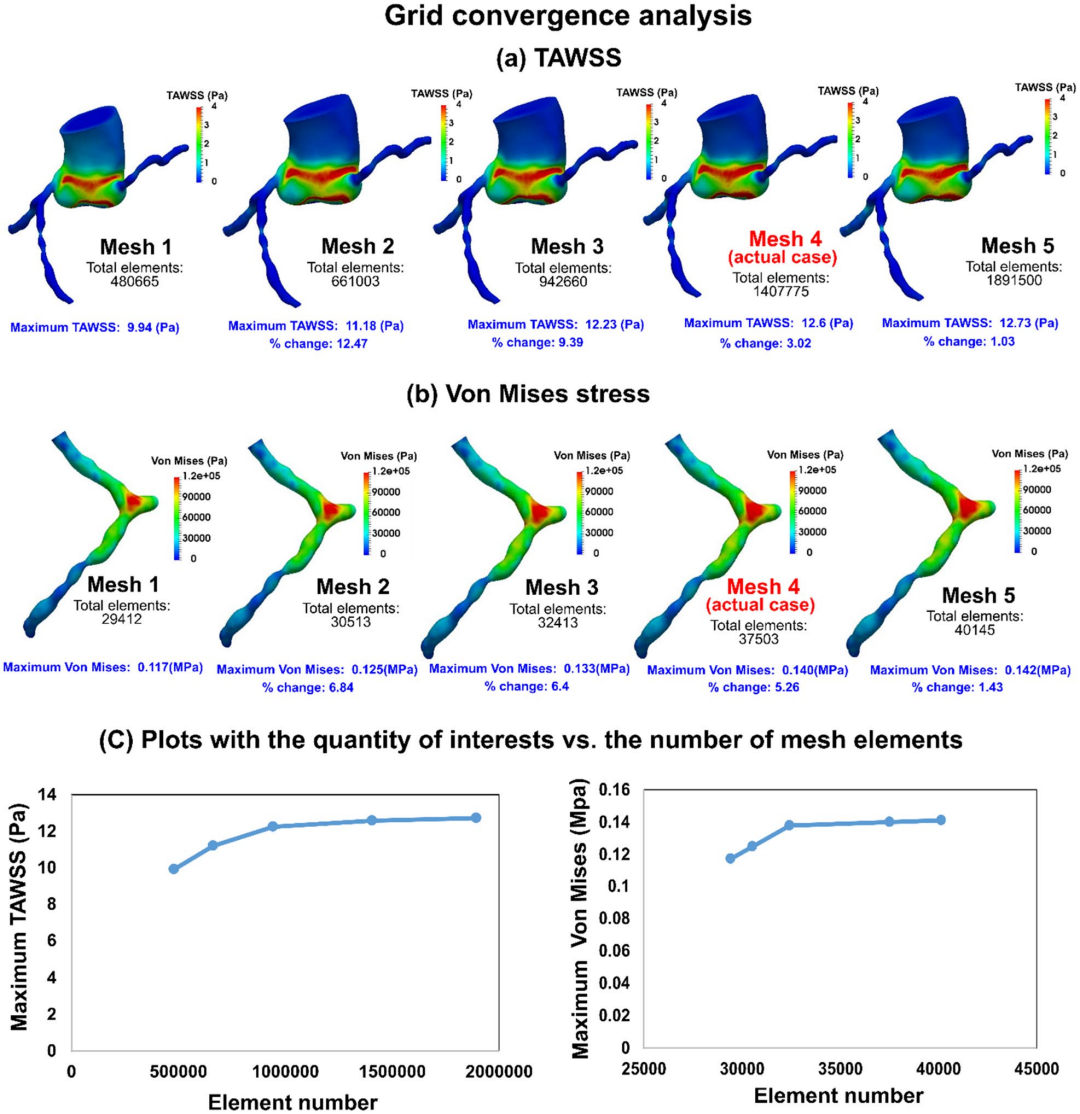
Grid convergence analysis. (**a**) TAWSS at the PVL location: TAWSS comparisons for different mesh resolutions at the peak of filling phase (maximum TAWSS): difference in peak TAWSS between mesh#3 and mesh#4 is 3% and between mesh#4 and #5 is 1.03%; (**b**) Von Mises stress: Von mises stress comparisons for different mesh resolutions of left coronary artery at the peak of filling phase: difference in maximum stress between mesh#3 and mesh#4 is 5.25% and between mesh#4 and #5 is 1.43%; (c) Plots for Maximum TAWSS and Maximum Von Mises stress versus the number of mesh elements.

**Figure 8:**
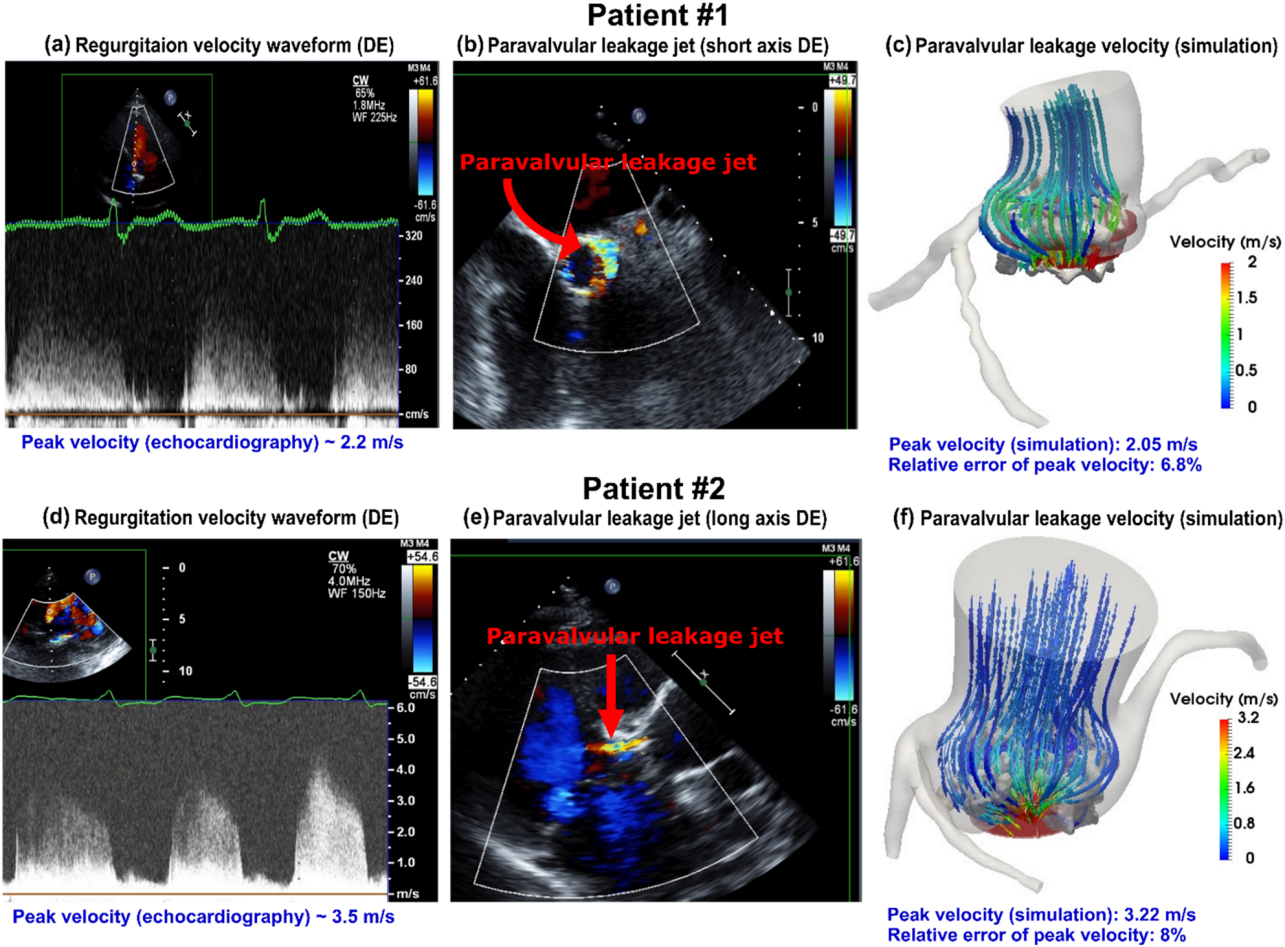
Validation: Doppler-based LPM and FSI framework versus patients Doppler echocardiography data. (**a**) & (**d**) Regurgitant flow waveform during diastole in patients #1 and #2; (**b**) Parasternal short axis view of PVL during diastole in patient #1; (**e**) Parasternal long axis view of PVL during diastole in patient #2; (**c**) & (**f**) PVL flow velocity streamlines during diastole in patients #1 and #2.

**Figure 9:**
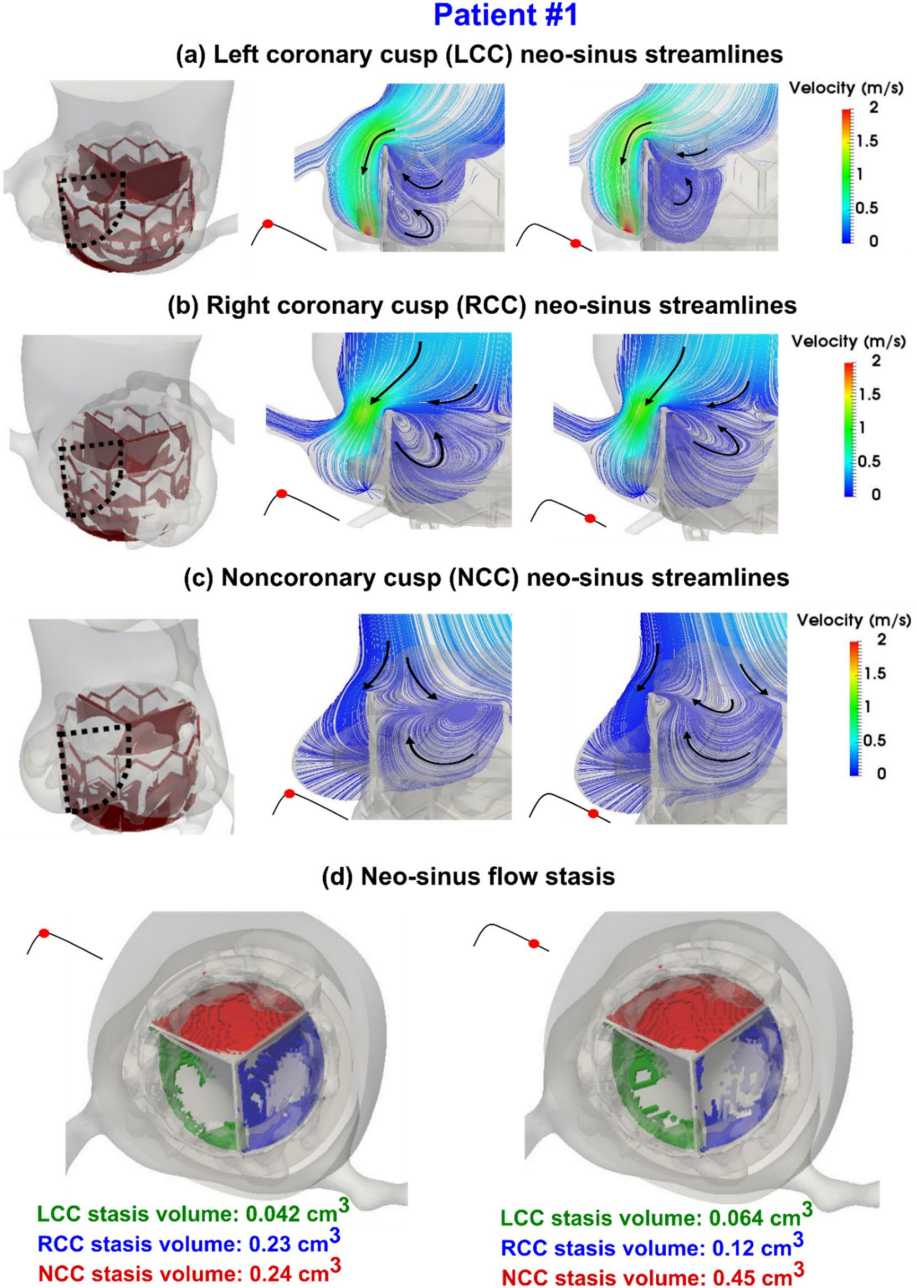
Evolution of vortical structure following TAVR in the aortic root and neo-sinus regions in patient #1 during diastole. (**a**) Mid-planar velocity of left coronary cusp neo-sinus; (**b**) Mid-planar velocity of right coronary cusp neo-sinus; (**c**) Mid-planar velocity of non-coronary cusp neo-sinus ; (**d**) Blood stasis volume. *Pre-TAVR*: severe aortic stenosis (EOA = 0.6 cm^2^), coronary artery disease and hypertension, diastolic dysfunction, atrial fibrillation, ejection fraction: 29%, brachial pressures: 61 and 107 mmHg, forward LV stroke volume: 35 mL; *Post-TAVR*: aortic valve (EOA = 1.7 cm^2^), hypertension, moderate mitral regurgitation, diastolic dysfunction, atrial fibrillation, ejection fraction: 34%, brachial pressures: 86 and 130 mmHg, forward LV stroke volume: 62 mL.

**Figure 10:**
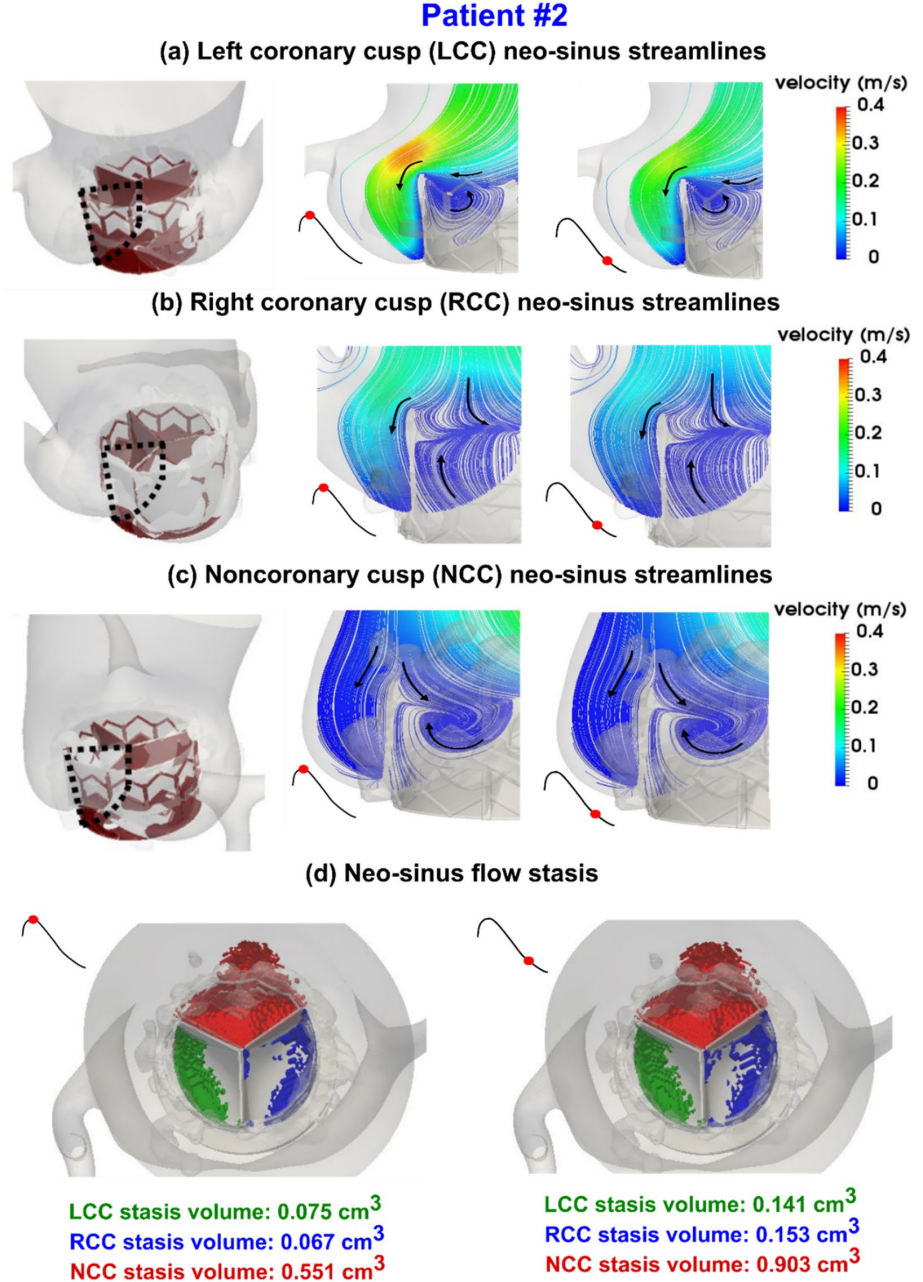
Evolution of vortical structure following TAVR in the aortic root and neo-sinus regions in patient #2 during diastole. (**a**) Mid-planar velocity of left coronary cusp neo-sinus; (**b**) Mid-planar velocity of right coronary cusp neo-sinus; (**c**) Mid-planar velocity of non-coronary cusp neo-sinus; (**d**) Blood stasis volume. *Pre-TAVR*: severe aortic stenosis (EOA = 0.6 cm^2^), coronary artery disease and dyslipidemia, diastolic dysfunction, ejection fraction: 38%, brachial pressures: 54 and 107 mmHg, forward LV stroke volume: 74 mL; *Post-TAVR*: aortic valve (EOA = 1.8 cm^2^), diastolic dysfunction, ejection fraction: 51%, brachial pressures: 59 and 120 mmHg, forward LV stroke volume: 92 mL.

**Figure 11:**
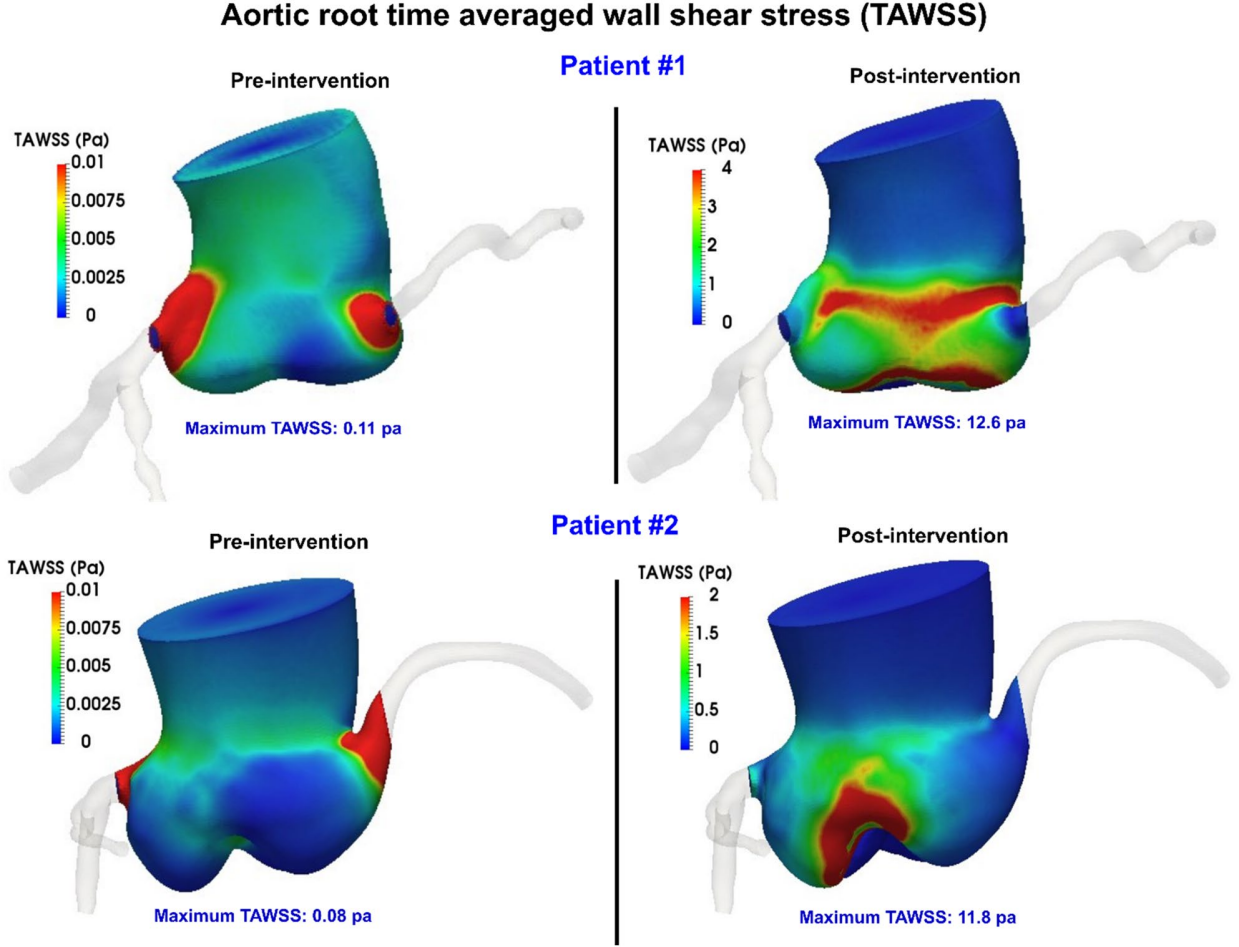
Time averaged wall shear stress of the aortic root during diastole for patients #1 and #2 in both pre and post intervention status.

**Figure 12:**
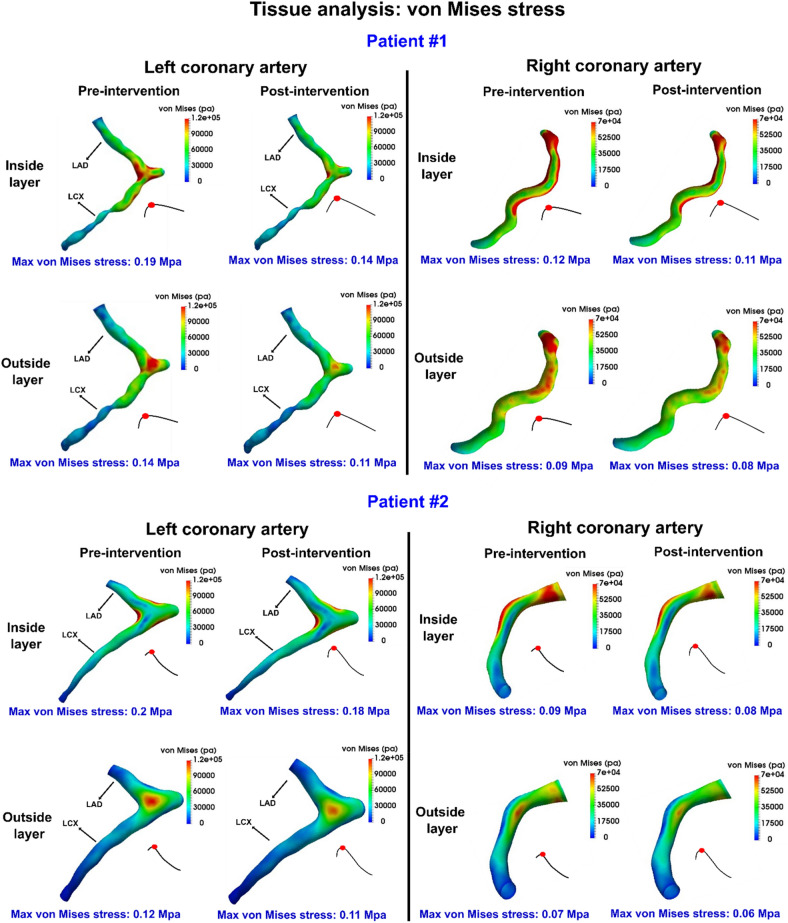
3D distribution contours of Mises stress at peak diastole in patient#1 and patient#2 between baseline and 90-day post-TAVR.

**Figure 13:**
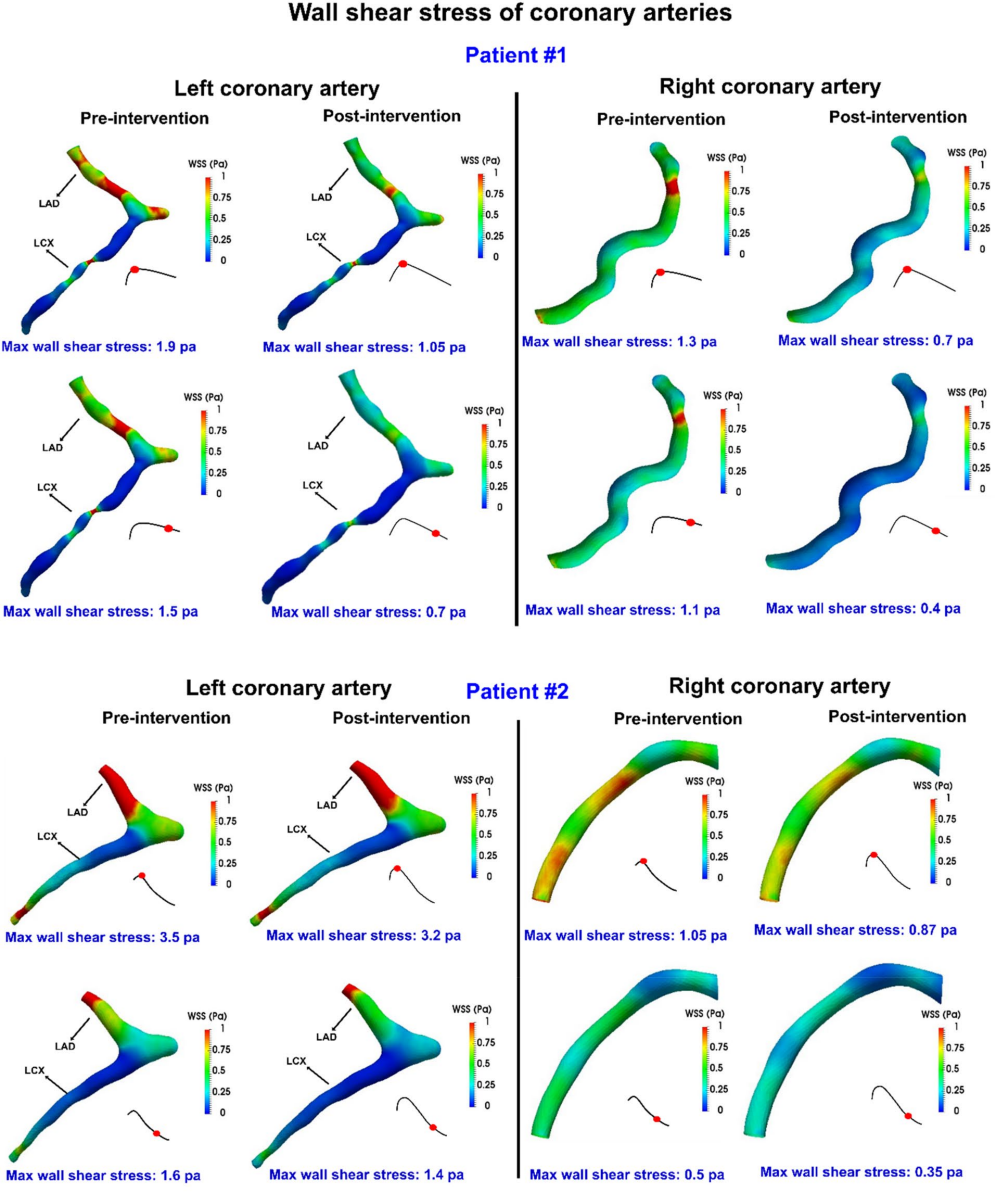
3D distribution contours of wall shear stress at peak diastole in patient#1 and patient#2 between baseline and 90-day post-TAVR.

**Figure 14:**
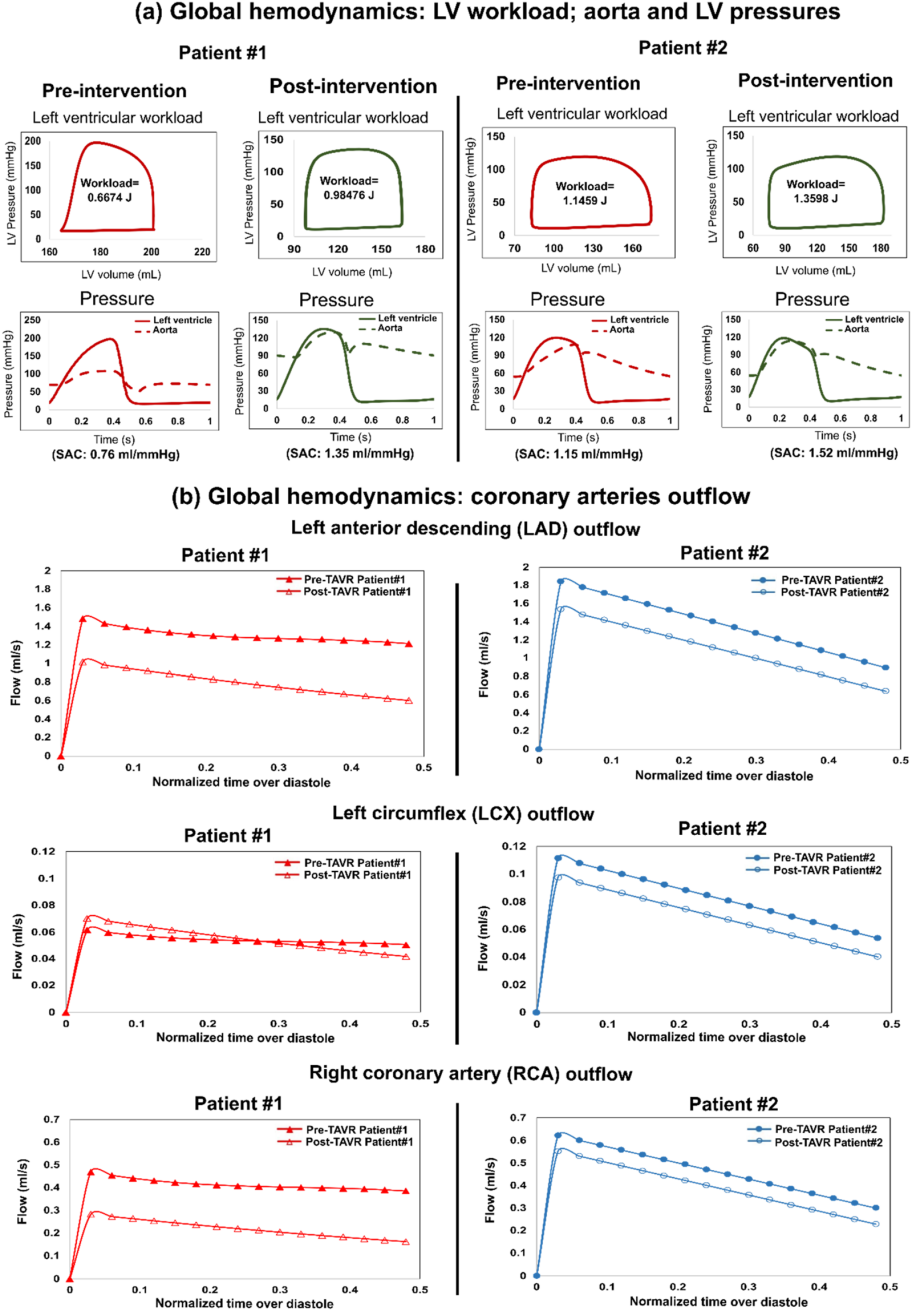
Global hemodynamics. Changes in predicted global hemodynamics before intervention and after TAVR for patients#1 and #2; (**a**) workload, left ventricle and ascending aorta pressure and systemic arterial compliance (SAC); (**b**) Changes in predicted coronary flowrate for LAD, LCX and RCA branches before intervention and after TAVR for both patients.

**Figure 15:**
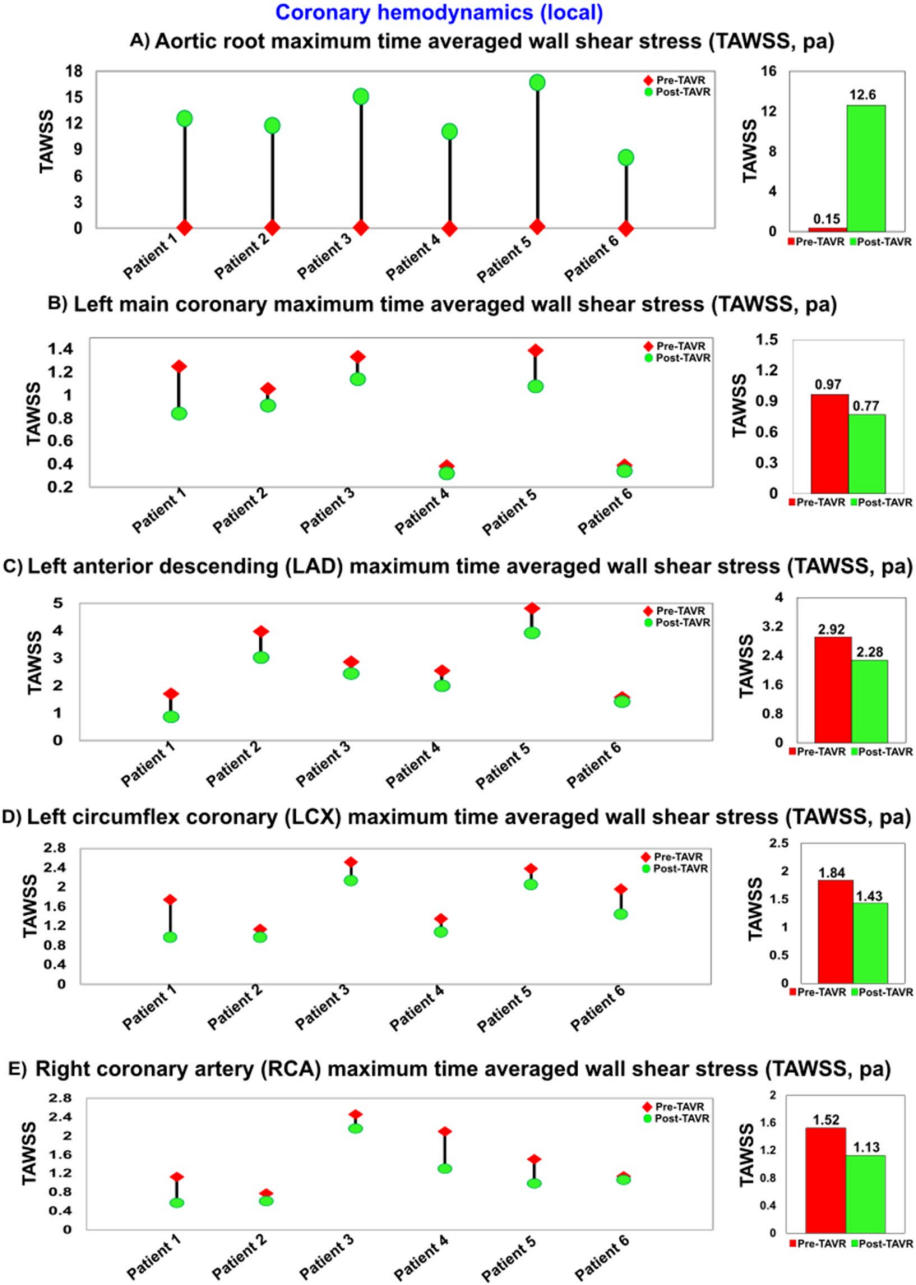
Changes in local hemodynamics in patients between baseline and 90-day post-TAVR (N = 6). (**a**) Aortic root maximum TAWSS; (**b**) Left main coronary maximum TAWSS; (**c**) Left anterior descending coronary artery maximum TAWSS; (**d**) Left circumflex coronary artery maximum TAWSS; (**e**) Right coronary artery maximum TAWSS.

**Figure 16:**
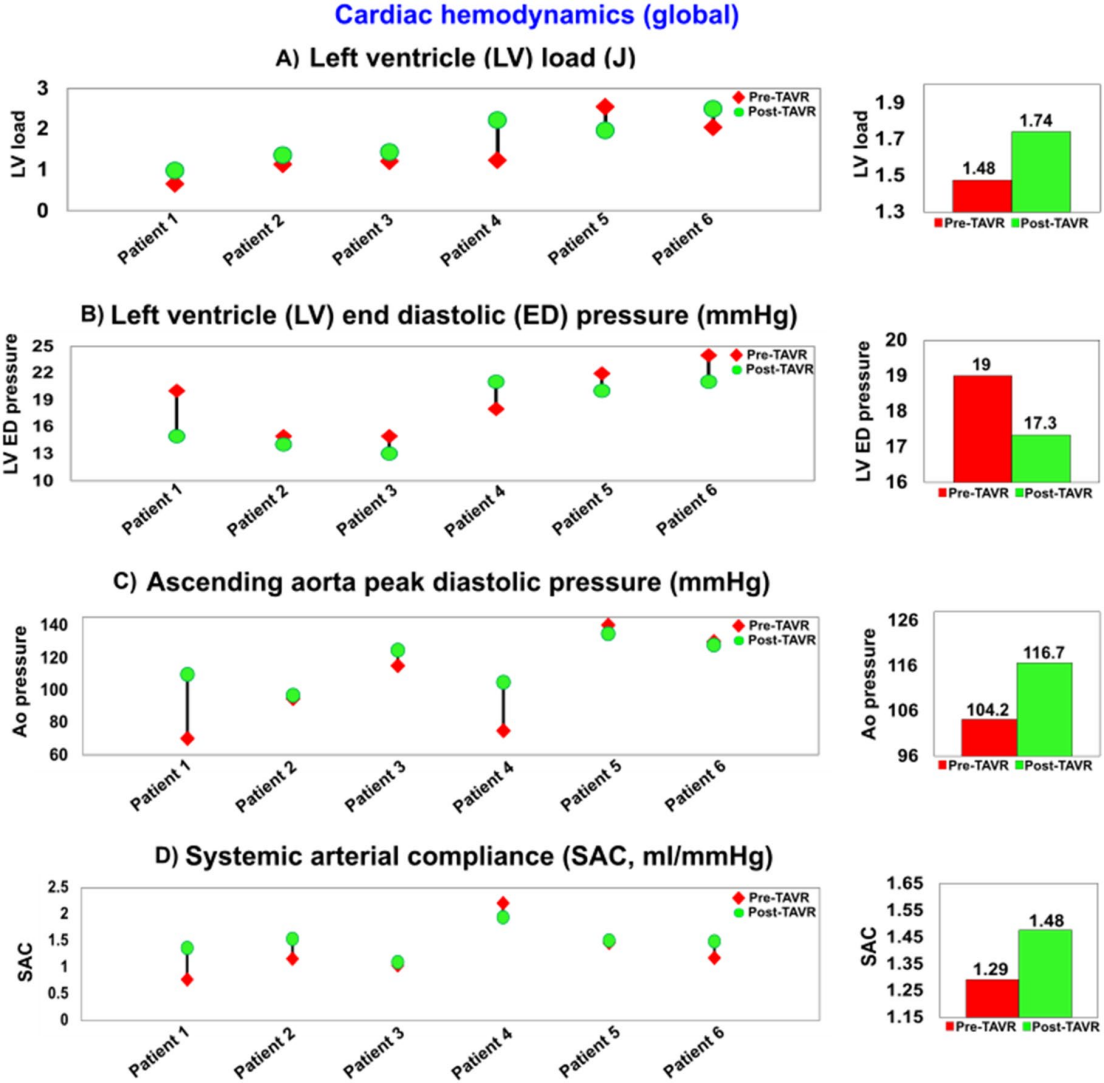
Changes in global hemodynamics (metrics of cardiac function & metrics of circulatory function) in patients between baseline and 90-day post TAVR (N = 6). (**a**) Left ventricle workload; (**b**) Left ventricle end diastolic pressure; (**c**) Ascending aorta peak pressure in diastole; (**d**) Systemic arterial compliance.

**Figure 17:**
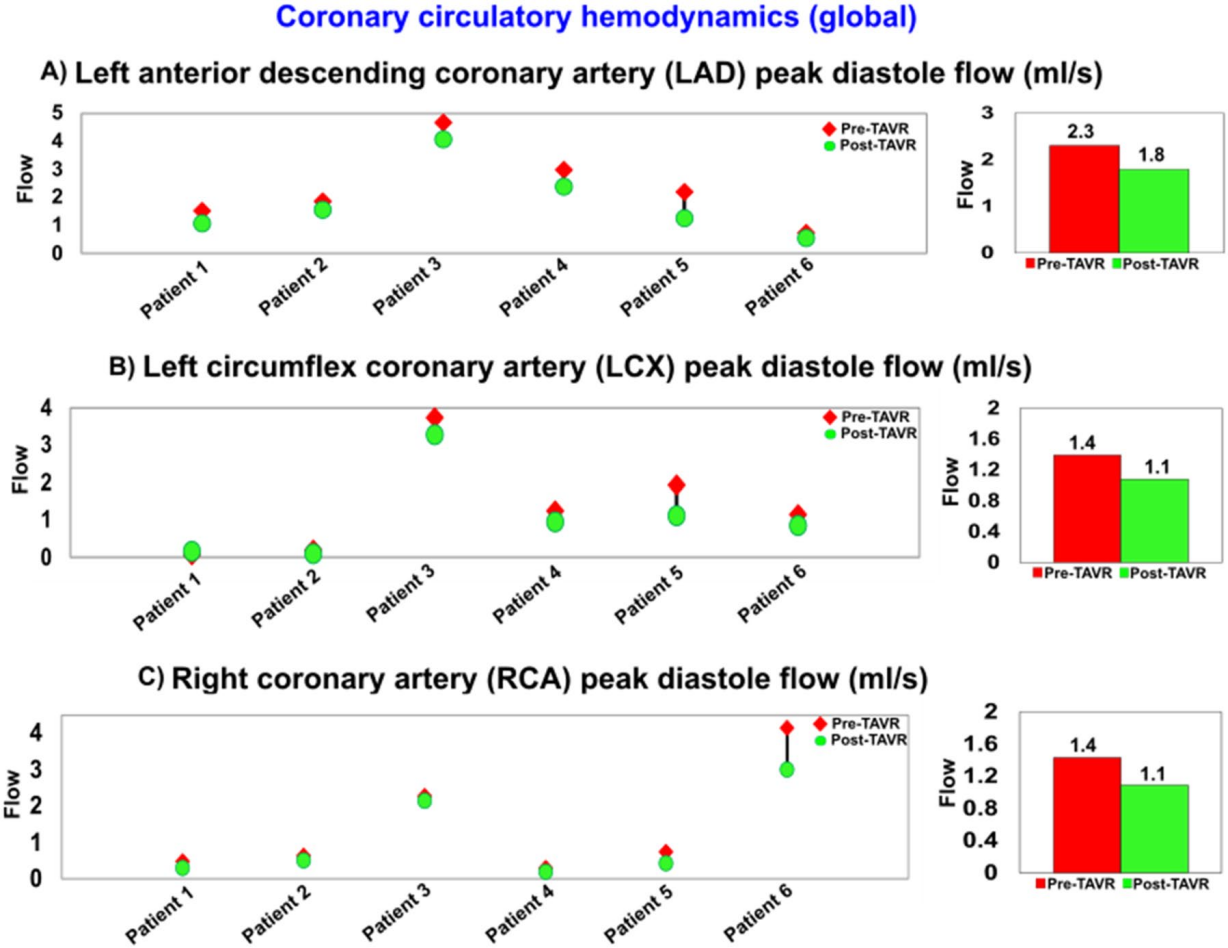
Changes in coronary circulatory hemodynamics in patients between baseline and 90-day post TAVR (N = 6). (**a**) Left anterior descending coronary artery peak diastolic flow; (**b**) Left circumflex coronary artery peak diastolic flow; (**c**) Right coronary artery peak diastolic flow.

## Materials and methods

### Clinical medical imaging

#### Study population & data acquisition

The study population included 6 patients with severe AS who received TAVR (Table 1; patients characteristics) in 2017 at St. Paul’s Hospital (Vancouver, Canada). The protocols were reviewed and approved by the Clinical Research Ethics Board (CREB) and informed consents were collected from all participants. The data was anonymized and transferred from St. Paul’s Hospital^12^ and the approval was granted by the CREB. Results were expressed as mean ± standard deviations (SD) (Table 1: Baseline patient characteristics).

**Table 1:** Baseline and post-TAVR patient characteristics.

C3VD patients (n = 6, mean ± SD)	Pre-TAVR (n = 6, mean ± SD)	Post-TAVR (n = 6, mean ± SD)
**Patient description**		
Mean age (years)	86 ± 3.55	86 ± 3.55
Gender	(Male: 5; Female: 1)	(Male: 5; Female: 1)
Mean weight (kg)	75 ± 12.9	N/A
Mean height (cm)	169 ± 14.5	169 ± 14.5
NYHA classifications		
Patient No. 1	Pre-TAVR: Grade 4	Post-TAVR: Grade 4
Patient No. 2	Pre-TAVR: Grade 3	Post-TAVR: Grade 3
Patient No. 3	Pre-TAVR: Grade 3	Post-TAVR: Grade 3
Patient No. 4	Pre-TAVR: Grade 4	Post-TAVR: Grade 4
Patient No. 5	Pre-TAVR: Grade 4	Post-TAVR: Grade 4
Patient No. 6	Pre-TAVR: Grade 2	Post-TAVR: Grade 2
**Arterial hemodynamics**		
Systolic arterial pressure (mmHg)	Pre-TAVR: 124.5 ± 8.5	Post-TAVR: 137.3 ± 9.5
Diastolic arterial pressure (mmHg)	Pre-TAVR: 64.5 ± 2	Post-TAVR: 65.75 ± 8
Coronary artery disease (CAD)	n = 6	n = 6
Hypertension (HTN)	n = 6	n = 6
Dyslipidemia	n = 6	n = 6
**Aortic valve hemodynamics**		
Aortic valve effective orifice area (cm^2^)	0.7 ± 0.14	1.63 ± 0.06
Stenotic aortic valve type	Tricuspid: 6; Bicuspid: 0	N/A
Prosthetic diameter size (mm)	N/A	23 ± 1.7
Prosthetic type		
Edwards SAPIEN	N/A	n = 6
Maximum aortic valve pressure gradient (mmHg)	Pre-TAVR: 43 ± 3.5	Post-TAVR: 21 ± 2.5
Mean aortic valve pressure gradient (mmHg)	Pre-TAVR: 25 ± 4	Post-TAVR: 16.5 ± 5.5
**Left ventricle hemodynamics**		
Ejection fraction (%)		
Patient No. 1	Pre-TAVR: 29	Post-TAVR: 34
Patient No. 2	Pre-TAVR: 49	Post-TAVR: 57
Patient No. 3	Pre-TAVR: 37	Post-TAVR: 58
Patient No. 4	Pre-TAVR: 57	Post-TAVR: 59
Patient No. 5	Pre-TAVR: 18	Post-TAVR: 22
Patient No. 6	Pre-TAVR: 63	Post-TAVR: 73
Heart rate (bpm)	Pre-TAVR: 53 ± 9	Post-TAVR: 69 ± 13

### Patient-specific lumped parameter algorithm for complex valvular, vascular, mini-vascular and ventricular diseases

We have previously developed a non-invasive diagnostic computational-mechanics framework for complex valvular, vascular and ventricular disease (called C3V-LPM for simplicity), described in detail elsewhere^21^. In this study, we further developed the C3V-LPM to enable the quantification of local and global hemodynamics in patients with mixed and complex valvular, vascular, mini-vascular and ventricular diseases (known as C3VM-LPM) (Fig. 1, Table 2). The developed framework uses limited input parameters that can all be reliably measured non-invasively using DE, CT and a sphygmomanometer. The previously created model, C3V-LPM, was validated against clinical catheterization data in forty-nine AS patients with a substantial inter- and intra-patient variability with a wide range of disease^21^. In addition, some of the sub-models of the patient-specific lumped parameter algorithm have been used previously^12,14,25–31,^, with validation against in vivo cardiac catheterization (N = 34)^32,33^ in patients with vascular diseases, in vivo MRI data (N = 57)^34^ in patients with AS, and in vivo MRI data (N = 23)^35,36^ in patients with coarctation and mixed valvular diseases.

**Table 2:** The Cardiovascular parameters used in the lumped parameter model.

Description	Abbreviation	Value
**Valve parameters**		
Effective orifice area	EOA	Measured using DE
Inertance (mitral valve)	M_MV_	Constant value: 0.53 gcm^−2^^37^
**Systematic circulation parameters**		
Aortic resistance	R_ao_	Constant value: 0.05 mmHg.s.mL^−1^^21,25,32–34^
Aortic compliance	C_ao_	Initial value: 0.5 mL/mmHg Optimized based on brachial pressures *(Systolic and diastolic brachial pressures are optimization constraints)*
Systemic vein resistance	R_SV_	0.05 mmHg.s.mL^−1^^21,25,32–34^
Systemic arteries and veins compliance	C_SAC_	Initial value: 2 mL/mmHg Optimized based on brachial pressures *(Systolic and diastolic brachial pressures are optimization constraints)*
systemic arteries resistance (including arteries, arterioles and capillaries)	R_SA_	Initial value: 0.8 mmHg.s.mL^−1^ Optimized based on brachial pressures *(Systolic and diastolic brachial pressures are optimization constraints)*
Upper body resistance	R_ub_	Adjusted to have 15% of total flow rate in healthy case^21,25,32–34^
Proximal descending aorta resistance	R_pda_	Constant value: 0.05 mmHg.s.mL^−1^^21,25,32–34^
Elastance function*		
Maximum Elastance	E_max_	2.1 (LV) 0.17 (LA)^38,39^
Minimum Elastance	E_min_	0.06 (LV, LA)^38,39^
Elastance ascending gradient	m_1_	1.32 (LV, LA)^38,39^
Elastance descending gradient	m_2_	27.4 (LV) 13.1 (LA)^38,39^
Elastance ascending time translation	${\tau }_{1}$	0.269 T (LV) 0.110 T (LA)^38,39^
Elastance descending time translation	${\tau }_{2}$	0.452 T (LV) 0.18 T (LA)^38,39^
**Pulmonary circulation parameters**		
Pulmonary Vein Inertance	L_PV_	Constant value:0.0005 mmHg s^2^ mL^−1^^37^
Pulmonary Vein Resistance	R_PV_	Constant value: 0.002 mmHg s mL^−1^^37^
Pulmonary Vein and capillary Resistance	R_PVC_	Constant value: 0.001 mmHg s mL^−1^^37^
Pulmonary Vein and Capillary Compliance	C_PVC_	Constant value: 40 mL/mmHg^37^
Pulmonary Capillary Inertance	L_PC_	Constant value: 0.0003 mmHg s^2^ mL^−1^^37^
Pulmonary Capillary Resistance	R_PC_	Constant value: 0.21 mmHg s mL^−1^^37^
Pulmonary Arterial Resistance	R_PA_	Constant value: 0.01 mmHg s mL^−1^^37^
Pulmonary Arterial Compliance	C_PA_	Constant value: 4 mL/mHg^37^
Mean Flow Rate of Pulmonary Valve	Q_MPV_	*Forward LVOT-SV* is the only input flow condition (measured using DE) *Q*_*MPV*_ *is a flow parameter that was optimized so that the lump-parameter model could reproduce the desirable DE-measured Forward LVOT-SV*
**Input flow condition**		
Forward left ventricular outflow tract stroke volume	Forward LVOT-SV	Measured using DE
**Output condition**		
Central venous pressure	P_CV0_	Constant value: 4 mmHg^21,25,32–34^
**Coronary parameters**		
Proximal Coronary Resistance	R_cor,p_	Adjusted based on MAP, CO and vessel cross sectional area^40–43^
Medial Coronary Resistance	R_cor,m_	Adjusted based on MAP, CO and vessel cross sectional area^40–43^
Distal Coronary Resistance	R_cor,d_	Adjusted based on MAP, CO and vessel cross sectional area^40–43^
Proximal Coronary Compliance	C_cor,p_	Adjusted based on total vessel compliance and cross-sectional area^40,42,44^
Medial Coronary Compliance	C_cor,m_	Adjusted based on total vessel compliance and cross-sectionalarea ^40,42,44^
**Other**		
Constant blood density	$\rho$	Constant value: 1050 kg/m^3^^21,25,32–34^
Heart rate	HR	Measured using DE
Duration of cardiac cycle	T	Measured using DE
Systolic End Ejection time	T_EJ_	Measured using DE
End diastolic volume	EDV	Measured using DE
End systolic volume	ESV	Measured using DE

#### Left ventricle

Time-varying elastance, E(t), is a common method to simulate the ventricle muscle stiffness which relates the LV pressure and volume:

1 $$E\left( t \right) = \frac{{P_{LV} \left( t \right)}}{{V\left( t \right) - V_{0} }}$$

where $P_{LV} \left( t \right)$, $V\left( t \right)$ and $V_{0}$ represent the LV time-varying pressure, time-varying volume and unloaded volume, respectively. This elastance function is often represented by the double Hill function, initially proposed by Stergiopulos et al.^38^. This mathematical function is capable of capturing the contraction and relaxation dynamics of the ventricle

2 $$E\left( t \right) = N\left( {\frac{{\left( {\frac{t}{{\tau_{1} }}} \right)^{{m_{1} }} }}{{1 + \left( {\frac{t}{{\tau_{1} }}} \right)^{{m_{1} }} }}} \right)\left( {\frac{1}{{1 + \left( {\frac{t}{{\tau_{2} }}} \right)^{{m_{2} }} }}} \right) + E_{min}$$

3 $$N = \frac{{E_{max} - E_{min} }}{2}$$

where $\tau_{1}$, $\tau_{2}$, $m_{1}$, $m_{2}$, $E_{max}$ and $E_{min}$ are ascending time translation, descending time translation, ascending gradient, descending gradient, maximum elastance and minimum elastance, respectively (see Table 2). The first term in brackets represents the contraction of the chamber and the second term in brackets represents the relaxation of the chamber. As outlined in Table 2: (1) $\tau_{1}$ and $\tau_{2}$ are functions of the cardiac cycle duration (T) and vary for each patient; (2) $m_{1}$, $m_{2}$ are constant for all patients (Stergiopulos et al.^38^, Mynard et al.^39^, Seemann et al.^45^). Parameter values used in the elastance function were adapted to generate physiological waveforms for pressure, volume and flow that can be found in Table 2^38,46–54^.

#### Left atrium

The same time-varying elastance function, E(t), used for the LV was applied to the LA for the coupling of LA pressure and volume. The parameter values used for the LA are found in Table 2, while the elastance function is defined by Eqs. (2) and (3)^21^.

#### Modeling heart valves

##### Aortic valve

The aortic valve was simulated using the net pressure gradient approach $\left( {PG_{net} } \right)$ across the aortic valve during systole:

4 $$\left. {PG_{net} } \right|_{AV} = \frac{2\pi \rho }{{\sqrt {\left. {E_{L} Co} \right|_{AV} } }}\frac{\partial Q\left( t \right)}{{\partial t}} + \frac{\rho }{{2\left. {E_{L} Co} \right|_{AV}^{2} }}Q^{2} \left( t \right)$$

and

5 $$\left. {E_{L} Co} \right|_{AV} = \frac{{\left( {\left. {EOA} \right|_{AV} } \right)A_{AO} }}{{A - \left. {EOA} \right|_{AV} }}$$

where $\left. { E_{L} Co} \right|_{AV}$, $\left. { EOA} \right|_{AV}$, $A_{AO}$, $\rho$ and $Q$ are the valvular energy loss coefficient, the effective orifice area, ascending aorta cross sectional area, blood density and transvalvular flow rate, respectively.

##### Aortic regurgitation

Aortic regurgitation (AR) was modeled as the difference between the aortic pressure and the LV pressure during diastole:

6 $$\left. {PG_{net} } \right|_{AR} = \frac{2\pi \rho }{{\sqrt {\left. {E_{L} Co} \right|_{AR} } }}\frac{\partial Q\left( t \right)}{{\partial t}} + \frac{\rho }{{2\left. {E_{L} Co} \right|_{AR}^{2} }}Q^{2} \left( t \right)$$

and

7 $$\left. {E_{L} Co} \right|_{AR} = \frac{{EOA_{AR} A_{LVOT} }}{{A_{LVOT} - EOA_{AR} }}$$

where $\left. {E_{L} Co} \right|_{AR}$, $EOA_{AR}$ and $A_{LVOT}$ are the regurgitation energy loss coefficient, regurgitant effective orifice area and LVOT area, respectively.

##### Mitral valve

The mitral valve (MV) was modeled using a similar technique to the aortic valve which accounts for the net pressure gradient ($\left. {PG_{net} } \right|_{MV}$) across the MV during LA ejection. $\left. {PG_{net} } \right|_{MV}$ is a function of $\rho$, $Q_{MV}$, $EOA_{MV}$ and $M_{MV}$, which represent the density of the fluid, the transvalvular flow rate, effective orifice area and inertance, respectively.

8 $$\left. {PG_{net} } \right|_{MV} = \frac{{M_{MV} }}{{EOA_{MV} }}\frac{{\partial Q_{MV} \left( t \right)}}{\partial t} + \frac{\rho }{{2\left. {EOA} \right|_{MV}^{2} }}Q_{MV}^{2} \left( t \right)$$

##### Mitral regurgitation

Mitral regurgitation (MR) was also modeled based on the difference between the mitral pressure and the LA pressure during diastole.

9 $$\left. {PG_{net} } \right|_{MR} = \frac{{M_{MV} }}{{EOA_{MR} }}\frac{\partial Q\left( t \right)}{{\partial t}} + \frac{\rho }{{2\left. {EOA} \right|_{MR}^{2} }}Q^{2} \left( t \right)$$

where $\left. {EOA} \right|_{MR}$ is the MR effective orifice area.

#### Pulmonary flow

A rectified sine curve with duration $t_{ee}$ and amplitude Q_MPV_ simulated the pulmonary valve flow waveform:

10 $$Q_{PV} \left( t \right) = Q_{MPV} \sin \left( {\frac{\pi t}{{t_{ee} }}} \right),{\text{t }} \le {\text{ t}}_{{{\text{ee}}}} ; Q_{PV} \left( t \right) = 0,{\text{t}}_{{{\text{ee}}}} < {\text{ t }} \le {\text{ T}}$$

where Q_MPV_, t_ee_ and T are the mean flow rate of the pulmonary valve, end-ejection time, and cardiac cycle time, respectively. It is important to note the only input flow condition that can be reliably measured using DE in this study is the forward left ventricular outflow tract stroke volume (*Forward LVOT-SV*). The lumped-parameter model could reproduce this DE-measured metric when Q_MPV_, the mean flow rate of the pulmonary valve, was optimized.

#### Coronary arteries

C3VM-LPM was developed to simulate blood flow rate at the outlets of the left anterior descending (LAD) artery, left circumflex (LCX) artery and right coronary artery (RCA), in addition to other regions of the heart and the cardiovascular system. The 5-element electrical circuit used in this study to model each coronary branch was developed by Kim et al.^40^ and has been used extensively to generate boundary conditions for higher order coronary models^41,42,44,55–58^.

The circuits comprised of 3 resistors ($$R_{cor,p} ,R_{cor, m} ,R_{cor,d} )$$, 2 capacitors ($$C_{cor,p} , C_{cor, m} )$$ and an embedded pressure source ($$P_{im} )$$ (Figs. 1 and 2). While inductors are often included in the heart and circulatory models, they were not included in this coronary model since the inertial phenomena is not significant in the coronary artery branches^59^. By including an embedded pressure source, this layout has been shown to capture the bi-phasic nature of coronary flow, in which peak blood flow occurs during the diastole phase rather than during the systole phase^40,59^.

The following ordinary differential equations (ODEs) are generated to model the coronary lumped parameter model^55^:

11 $${\text{q}}_{{{\text{in}}}} = { }\frac{{{\text{P}}_{{{\text{in}}}} - {\text{ P}}_{{\text{p}}} }}{{{\text{R}}_{{{\text{cor}},{\text{p}}}} }}$$

12 $${\text{q}}_{{{\text{in}}}} = {\text{C}}_{{{\text{cor}},{\text{p}}}} \frac{{{\text{dP}}_{{\text{p}}} }}{{{\text{dt}}}} + {\text{q}}_{{\text{m}}}$$

13 $${\text{P}}_{{\text{p}}} = {\text{q}}_{{\text{m}}} {\text{R}}_{{{\text{cor}},{\text{m}}}} + {\text{P}}_{{\text{m}}}$$

14 $${\text{q}}_{{\text{m}}} = {\text{q}}_{{{\text{out}}}} + {\text{C}}_{{{\text{cor}},{\text{m}}}} \frac{{{\text{dP}}_{{{\text{im}}}} }}{{{\text{dt}}}}$$

15 $${\text{P}}_{{\text{m}}} = {\text{q}}_{{{\text{out}}}} {\text{R}}_{{{\text{cor}},{\text{d}}}} + {\text{P}}_{{{\text{out}}}}$$

where ${\text{q}}_{{{\text{in}}}}$, ${\text{P}}_{{{\text{in}}}} , {\text{q}}_{{{\text{out}}}}$ and ${\text{P}}_{{{\text{out}}}}$ are the blood flow and pressure into and out of the coronary branch. $R_{cor,p} ,R_{cor,m} ,R_{cor,d}$ are the proximal, medial, and distal resistors while $C_{cor,p} , C_{cor,m}$ are the proximal and medial capacitors. ${\text{P}}_{{\text{p}}}$, ${\text{P}}_{{\text{m}}}$ and $P_{im}$ are the proximal, medial and intramyocardial pressures.

$P_{im}$ is set to be either the left ventricle (LV) or right ventricle (RV) pressure, depending on the coronary artery that it is coupled to. In this study, we used the LV pressure for the left branches (LAD and LCX) and $0.5P_{LV}$^40^ to create the RV pressure for the right branch (RCA).

##### Determining arterial resistance and compliance in coronaries

*Total coronary resistance* The total coronary resistance was derived from a relationship between blood pressure and blood flow, where the mean flow rate to the coronary arteries was assumed to be 4.0% of the cardiac output^40,41^:

16 $$R_{cor,total} = \frac{MAP}{{Q_{cor,total} }} = \frac{MAP}{{\left( {0.04} \right){*}CO}}$$

where $R_{cor,total}$ is the total coronary resistance. Mean arterial pressure (MAP) is calculated based on systolic blood pressure (SBP), diastolic blood pressure (DBP) and heart rate (HR)^60^:

17 $$MAP = DBP + \left[ {\frac{1}{3} + \left( {HR*0.0012} \right)} \right]\left( {SBP - DBP} \right)$$

*Coronary vessel resistance and compliance* The total coronary resistance was divided between each of the branches based on a generalization of Murray’s law^43^, which relates resistance to vessel diameter:

18 $$R_{cor,j} = \frac{{\mathop \sum \nolimits_{i = 1}^{n} \sqrt {A_{i} }^{2.6} }}{{\sqrt {A_{j} }^{2.6} }} R_{cor,total }\; where\; j = \left\{ {LAD, LCX\; or\; RCA} \right\}$$

where $R_{cor,j}$ is the total coronary resistance in the desired vessel and $A_{i}$ is the cross sectional area of each of the coronary vessels^40^. Further division of the total vessel resistance into the 3 resistive elements in the circuit was based on the work of Sankaran et al.^42^:

19 $$R_{cor,j,p } = \left( {0.32} \right)R_{cor,j} \quad R_{cor,j,m } = \left( {0.52} \right)R_{cor,j} \quad R_{cor,j,d } = \left( {0.16} \right)R_{cor,j}$$

where $R_{cor,j,p } , R_{cor,j,m } , R_{cor,j,d }$ are the proximal, medial, and distal resistors.Similarly, the left coronary vessel compliances were computed by dividing up the total left coronary compliance based on vessel diameters:

20 $$C_{cor,j} = \frac{{A_{j} }}{{\mathop \sum \nolimits_{i = 1}^{n} A_{i} }} C_{cor,total }^{L}$$

where $C_{cor,j}$ is the left coronary vessel compliance, $C_{cor,total }^{L}$ is the total left coronary compliance and $A_{i}$ is the cross sectional area of each of the left coronary branches^40^. A manual tuning process was utilized to determine total left coronary compliance value that lead to physiological coronary flow waveforms^44,61,62^.

The compliances were then divided across the 2 capacitors based on the following relationship^42^:

21 $$C_{cor,j,p} = \left( {0.11} \right)C_{cor,j} \quad C_{cor,j,m} = \left( {0.89} \right)C_{cor,j}$$

where $C_{cor,j,p}$ and $C_{cor,j,m}$ are the proximal and medial capacitors. The same process was employed for the right coronary vessels.

#### Input parameters

The following patient-specific parameters were inputs for the C3VM-LPM algorithm: forward left ventricular outflow tract stroke volume (Forward LVOT-SV), cardiac cycle time, ejection time, aortic valve effective orifice area (EOA), mitral valve EOA, ascending aorta cross sectional area, left ventricle outflow tract area, EOA during aortic regurgitation and EOA during mitral regurgitation measured by DE as well as brachial systolic and diastolic pressures measured by a sphygmomanometer. All the details about patient-specific parameter estimation were described in^21^. In addition, coronary geometry dimensions (left main coronary artery (LMCA) average diameter, LAD coronary artery average diameter, LCX coronary artery average diameter and right coronary artery (RCA) average diameter), measured from the patient-specific reconstructed coronary artery geometry using CT data, are the input parameters for the C3VM-LPM algorithm.

#### Computational algorithm

MathWorks Simscape (MathWorks, Inc.) was used to formulate and solve the system of ordinary differential equations (ODEs) which govern the lumped parameter circuit. Additional functions were written in Matlab and Simscape to supplement and enhance the Simscape code. The ode23t trapezoid rule variable step solver with an initial time step of 0.1 ms was used to solve these ODEs. Initially the voltages and currents in the capacitors and inductors were set to zero and the model was run for ~ 150 cycles to reach a steady state. For the patient specific optimization, the residual criterion was set to 10^−6^.

We generated a signal to model LV elastance using a double Hill function representation of a normalized elastance curve for human adults^21^. The LV pressure, P_LV_, calculated using the initial values of the model input parameters from Table 2, and the time-varying elastance (Eq. 1), were used to compute the instantaneous LV volume, V(t). Subsequently, the time derivative of the instantaneous LV volume was calculated to find the LV flow rate. This approach was also applied to obtain the volume of the left-atrium, pressure, and flow rate.

#### Patient-specific response optimization

To correctly patient-variability, four parameters of the model were optimized such that the lumped-parameter model reproduced the physiological measurements obtained from the patient. Simulink Design Optimization toolbox was used to optimize the response of the lumped-parameter model based on Matlab’s fmincon function.

Since *Forward LVOT-SV* can be measured reliably using DE, Q_MPV_ was tuned to minimize the difference between the *Forward LVOT-SV* calculated by the model and the one measured using DE in each patient:

22 $${\text{Forward LVOT-SV}} = A_{LVOT} VTI_{LVOT} = \frac{{\pi (D_{LVOT} )^{2} }}{4}VTI_{LVOT}$$

where $D_{LVOT}$, $A_{LVOT}$, and $VTI_{LVOT}$ are LVOT diameter, LVOT area, and LVOT velocity–time integral, respectively ^21^.

In the second step, R_SA_, C_SAC_, and C_ao_ were optimized so that maximum and minimum aortic pressures were respectively equal to the systolic and diastolic pressures measured using a sphygmomanometer in each patient. Since the left ventricle experiences the total systemic resistance rather than the individual resistances, and the systemic arteries resistance, $R_{SA}$, is one order of magnitude greater than both the aortic resistance, $R_{ao}$, and systemic vein resistance, $R_{SV}$, for the sake of simplicity we considered $R_{ao}$ and $R_{SV}$ as constants and optimized $R_{SA}$ as the main contributor of the total systemic resistance. *C*_*ao*_ was considered to be 0.6 of C_SAC_ because 60% of the total arterial compliance resides in the proximal aorta^63^.

End systolic volume (ESV) or end diastolic volume (EDV) measured by DE was fed to the lumped-parameter model to adjust only starting and ending volumes in the P–V loop diagram. For this purpose, the Biplane Ellipsoid model was used to calculate the instantaneous LV volume at the end of diastole or systole as follows^21^:

23 $$\forall = { }\frac{{A_{1} { }A_{2} }}{{(L_{1} + L_{2} )/2}}$$

where A_1_, A_2_, L_1_ and L_2_ are LV area measured in the apical four-chamber view, LV area measured in the apical two-chamber view, LV length measured in the apical four-chamber view and LV length measured in the apical two-chamber view, respectively.

In addition, we conducted an extensive parameter sensitivity analysis that revealed changes in pulmonary parameters (e.g., C_PVC_) have negligible effects on the model output variables. Therefore, we did not include these pulmonary parameters in the parameter-optimization process and considered them as constants given in Table 2.

### Fluid–structure interaction simulation study

The blood flow inside the coronary arteries was simulated using similar 3-D FSI set-up as described in our previous works^18,19^, using open-source FOAM-Extend library^64^. The transcatheter aortic valve (TAV) frame and aortic wall are assumed to be rigid during diastole^65,66^. All details about governing equations, FSI method and modeling were presented in Supplementary Materials.

#### Boundary conditions

Previous studies^65,67,68^ have used time-dependent pressure waveform of ascending aorta and ventricle (assuming fixed and rigid valve and aorta) to obtain the main hemodynamic features of PVL during diastole. However, the accurate and patient specific time-dependent pressure waveform is necessary for any CFD or FSI simulation as the whole topology of fluid domain is affected by pressure waveforms, and that’s why a patient-specific pressure waveform is crucial for an accurate simulation. Our patients specific lumped parameter algorithm generated boundary conditions (Figs. 1 and 2) non-invasively to provide^21^: (1) the time-dependent pressure waveform of ascending aorta during diastole which was applied as inlet boundary condition; (2) the time-dependent coronary flow waveforms which were applied as coronary outlet boundary condition; (3) for PVL, the ventricle pressure was applied as outlet at the leakage area location (leakage area was measured and located based on short axis DE, Fig. 3b) to provide patient-specific PVL pressure gradient (pressure difference between ascending aorta and ventricle during diastole). For the PVL simulation, we used the same approach in previous studies^67,68^. The leakage surface is the interface wall between aortic root (behind the TAV stent) and LVOT which is considered for outlet boundary condition. This surface is in the vicinity of the outer region of TAV stent. Therefore, the blood flow was not free (i.e., pressure zero in the outlet) to move towards ventricle. Instead, the blood flow was driven towards the LVOT (and ventricle) by pressure gradient between ascending aorta and left ventricle during diastole (Fig. 4). All the simulations were performed during diastole and the aortic valve was therefore assumed to be rigidly closed since the large deformation of valve and aorta occurs mainly during systole, while they remain relatively motionless during diastole with negligible effect of detailed closing shape of the leaflets on the PVL^68,69^. It is important to note that PVL occurs only during diastole^70^ and previous studies^67,68,71^ have performed the simulation only for diastole under assumption of rigid valves/aorta^65,67,71^ and found that this assumption does not affect the conclusions of their studies. They have also validated their results with experimental and clinical data^65,67,71^. We followed the same approach^65,67,71^ in our study and performed validation ("Validation: Doppler-based LPM and FSI framework versus clinical Doppler echocardiography data" section) with clinical DE velocity magnitude. However, we acknowledge that this a limitation of our study and we addressed this in the limitation section. Yet, it is worth mentioning that in the absence of essential characterization of patient-specific material properties required for FSI simulation of valve flow (which is the case in all ongoing FSI simulations^69,72,73^), the results of FSI simulation could be an incorrect representative of the flow^66^. Therefore, whether the FSI simulation of the valve and aorta with such limitations improves the results is still debatable especially if the end goal is to provide a patient-specific framework^74^.

### Validation: doppler-based LPM and FSI framework versus clinical Doppler echocardiography data

Figure 8a to f compares the peak PVL velocity simulated using our computational framework and DE data for two patients as a sample (8a and 8d: regurgitant flow waveform 8b: parasternal short axis view of PVL jet; 8e: parasternal long axis view of PVL jet). The simulated peak velocities are in a good agreement with the ones measured by DE in both patients with a maximum relative error of 8% for the peak velocity at the beginning of diastole phase (early filling). For the whole diastole phase, the results show good agreements between velocity calculated using the computational framework and the ones measured using DE in both cases investigated in this study.

## Results

### Effect of anatomic and deployment characteristics on aortic root and neo-sinus local hemodynamics (post-TAVR)

The blood flow vortical structure and stagnation in the aortic root, sinus of Valsalva and neo-sinus region depends on the ascending aorta and ventricular pressures, aortic root geometry, aortic valve geometry, stent height, deployment angle and coronary ostium location. We investigated hemodynamic metrics computed by our computational framework (Figs. 9 and 10) as follows:

#### Vortical structure

It has been shown that for a TAVR without PVL, the coronary flow influences the flow patterns of aortic root and neo-sinus and favors the transfer of blood flow towards ostium during diastole^75^. However, our results showed that in the presence of PVL, the aortic root vortices will not favor the transfer of blood flow towards ostium in the aortic root and neo-sinus region. As shown in Figs. 9a,b,c and 10a,b,c, for patients #1 and #2, the mainstream of PVL flow originates from ascending aorta towards the leakage orifice behind the stent and between LCC and RCC leaflets with a maximum of 2.05 m/s and 3.22 m/s for patients #1 and #2 respectively. However, the maximum velocity between left ostium and stent was 1.53 m/s and 0.42 m/s for patients #1 and #2 respectively. This can be explained by the fact that the size of the gap between the edge of stent frame and the ostium is smaller for patient #1 than patient #2, leading to higher divergent velocity towards the leakage area.

For both patients, a vortex forms in the neo-sinus region of all the leaflets (LCC, RCC and NCC) as shown in Figs. 9a,b,c and 10a,b,c. Our results showed this vortex is very different for LCC, RCC and NCC and for different patients. For patient #1, the vortex arises from the leaflet surface at early diastole and dominates the whole neo-sinus region at late diastole, leading to an efficient washout of blood flow from the LCC (Fig. 9a). However, during the whole diastole, the center of vortex remains close to the stent edge for RCC (Fig. 9b), and for NCC (Fig. 9c), the center of vortex remains close to the upper commissure and vanishes at late diastole. For both RCC and NCC (Fig. 9b,c), the vortex does not move down to reach the leaflet surface, leading to a reduced washout of blood flow. For patient #2 though, the vortex center remains distant from the leaflets for LCC (Fig. 10a) and NCC (Fig. 10c) at early diastole and gets closer to the leaflets for RCC (Fig. 10b). Although in mid diastole, the vortex size in LCC increases, and does not dominate the whole neo-sins similar to the vortex for patient #1. In other words, for patient #2, vortices aid the washout in RCC more than LCC and NCC.

#### Stagnant and low-velocity flow

For patients #1 and #2 as a sample, NCC neo-sinus had higher regions of stagnant flow than RCC and LCC neo-sinuses; 0.24 cm^3^ at early diastole and 0.45 cm^3^ at late diastole for patient#1 (Fig. 9d), and 0.55 cm^3^ at early diastole and 0.903 cm^3^ at late diastole for patient#2 (Fig. 10d). For patient #1 the RCC had significantly higher stagnant flow than LCC for the whole diastole; 0.23 cm^3^ and 0.042 cm^3^ at early diastole and 0.12 cm^3^ and 0.064 cm^3^ at late diastole for RCC and LCC respectively. However, for patient #2, the LCC had slightly higher stagnant flow at early diastole and RCC had slightly higher stagnant flow at late diastole; 0.075 cm^3^ and 0.067 cm^3^ at early diastole and 0.141 cm^3^ and 0.153 cm^3^ at late diastole for LCC and RCC respectively. Interestingly, although patient #1 had more severe PVL than patient #2, the stagnant flow volume in the neo-sinus region was almost 2 folds larger for patient #2 than patient #1 (LCC and NCC). This can be explained by the fact that the blood stasis depends not only on the PVL severity, but also on the patient-specific aortic root geometry, ascending aorta and LV pressures and the deployment details of TAVR. In other words, our results showed that PVL severity alone cannot reveal the risk of thrombosis in the neo-sinus region.

#### Aortic root wall shear stress

Wall shear stress, as a tangential force induced by blood flow, has a major influence on regulating endothelial function^76^. In general, very high wall shear stress (typically higher than 3 Pa) could contribute to tissue rupture^77^. PVL could disturb the flow in the aortic root sinus after TAVR, leading to increased wall shear stress. As an example, the maximum local TAWSS at the aortic root was increased drastically after TAVR for patients #1 and #2 (Fig. 11); from 0.11 Pa and 0.08 Pa pre-TAVR to 12.6 Pa and 11.8 Pa post-TAVR for patient #1 and patient #2 respectively. Such considerably high TAWSS might be a concern for patients who received TAVR. Moreover, our finding showed that the distribution of wall shear stress at the aortic root is very different for each patient, depending on the characteristics of TAVR deployment and aortic root geometry (Fig. 11).

### Coronary arteries blood flow and tissue assessment (pre-TAVR and post-TAVR)

In the presence of PVL after TAVR, the supplied blood flow through the coronary arteries is altered. We investigated the metrics of tissue (solid domain) and flow (fluid domain) computed by strongly coupled FSI algorithm as follows:

#### Coronary arteries von-mises stress

Although there is no cut-off threshold available in the literature for the rupture of arterial wall von-Mises stress, an average stress of 0.3 MPa has been reported to initiate the first crack in the artery wall^78^. As shown in Fig. 12, the distribution of von-Mises stress and its maximum, is different for inner and outer layers of tissue. The maximum von-Mises stress magnitude for all coronary branches in our study was less than 0.3 MPa during diastole for both pre-TAVR and post-TAVR. A universal reduction in maximum von-Mises stress was observed after TAVR for all coronary arteries; As an example, 26.3% reduction for left coronary branches and 11.11% reduction for right coronary in patient #1, and 10% reduction for left coronary branches and 14.3% reduction for right coronary in patient #2.

#### Coronary arteries wall shear stress

Endothelial cells which are exposed to low wall shear stress display a pro-inflammatory state, which is associated with plaque progression^76,79^. Although providing an exact cut-off value for low wall shear stress is still challenging, some studies suggested that wall shear stress lower than 1 Pa^76^ or 1.2 Pa^79^ is associated with higher plaque progression in a further serial study of coronary atherosclerosis. We calculated the wall shear stress over diastole for all patients in pre and post TAVR states. For patients#1 and #2 as examples, local and maximum wall shear stress were decreased for all coronary branches (LCX, LAD and RCA) as shown in Fig. 13. For patient #2, the maximum wall shear stress slightly reduced for LAD and LCX branches; 8.5% and 12.5% for early and late diastole. However, for RCA, maximum wall shear stress decreased from 1.05 to 0.87 Pa (17%) at the peak diastole and from 0.5 to 0.35 Pa (30%) at late diastole. For patient #1, the maximum wall shear stress in the LAD and LCX branches reduced from 1.9 to 1.05 Pa (44.7%) at the peak diastole and from 1.5 to 0.7 Pa (53.3%) at late diastole. Also, for RCA, maximum wall shear stress decreased significantly from 1.3 to 0.7 Pa (46.2%) at the peak diastole and from 1.1 Pa to 0.4 Pa (63.6%) at late diastole. Such considerable reduction in wall shear stress in a coronary artery (lower than 1.2 Pa) could promote plaque progression for patients who received TAVR treatment.

### Computed global hemodynamics

#### Cardiac function

LV workload represents the total energy required by the ventricle to eject blood, and is an effective metric of LV load and clinical state^12,14,18,19^. For patients #1 and #2 for example, despite the reduction of transvalvular pressure gradient, the LV workload increased after TAVR due to the presence of PVL (Fig. 14a); 35% and 18.67% increase in workload after TAVR for patient #1 and #2 respectively. Although the LV pressure decreased post-TAVR, severe PVL contributed to a shift from ventricular pressure overload to a ventricular volume overload.

#### Circulatory function

Systemic arterial compliance (SAC) is an index for predicting vascular disease states. For patients with AS, a low SAC (lower than 0.64 ml/m^2^/mmHg) is associated with increased risk of morbidity^80^. As shown in Fig. 14a, SAC improved for patients #1 and #2 after TAVR, with SAC increasing to > 1 (ml/mmHg) for both patients after intervention.

Increased aortic pressure is expected after TAVR as a result of the removal of AS obstruction ^81,82^. As shown in Fig. 14a, maximum aortic pressure increased 57% for patient #1 after TAVR. For patient #2, however, maximum aortic pressure increased only 5.1%. Moreover, maximum left atrium pressure reduced by 39% for patient #1, while the change was almost negligible (less than 3% increase) for patient #2.

#### Coronary circulatory function

Inadequate coronary flowrate and coronary hypoperfusion could lead to exacerbated heart failure^83^. It has been shown that the TAVR deployment characteristics (such as implant depth, angle and PVL) could affect the coronary flow^83–86^. As shown in Fig. 14b, for all patients in our study, although the perfusion pressure has increased after TAVR, the PVL and flow disturbance in the aortic root significantly reduced the flowrate in almost all coronary branches. For example, maximum flowrate was reduced by 34% and 37% in LAD and RCA branches of patient #1 after TAVR. For LCX branch in patient#1, the flowrate remained almost unchanged, however, the flow in this branch was initial significantly reduced before TAVR because of the stenosis in the middle section of the artery (peak flow for LCX was 0.062 mL/s, while for LCA and RCA is 1.5 mL/s and 0.48 mL/s respectively). For patient #2, the maximum flowrate was reduced by 19% in LAD, 17% in LCX, and 14% in RCA branches. Even after the maximum flowrate at the peak diastole, the flow reduction persists for all coronary branches during the whole diastole for both patients after TAVR (Fig. 14b). Such considerable reduction of flow could contribute to cases of ischemic lesions and promote thrombus formation.

## Discussion

CAD is present in approximately 50% of the TAVR population, but this has decreased as the use of TAVR has migrated towards younger patients^83^. The question, however, of if CAD should be treated or reduced in severity prior to TAVR is still a topic for debate^83^. Additionally, TAVR and percutaneous coronary intervention (PCI) can be performed in parallel, which may reduce mortality as well as the number of vascular punctures required but may also require a larger volume of contrast agent, which could place additional strains on the kidneys^83^.

Coronary arteries are supplied with blood mainly during diastole, and due to the disturbed flow associated with PVL^87^, blood entering the coronary circulation may be disrupted. The complications resulting from this is relatively unknown, and more research is needed. Hemodynamic changes, which were assessed using non-invasive computational models in this paper, may provide insight into health complications following TAVR, which may go undetected in purely anatomical examinations^88^. In the present work, there are several findings which should be individually discussed:

### Improvements of coronary perfusion pressure and systemic arterial compliance after TAVR are poor indicators of coronary flow recovery in presence of paravalvular leak

AS disrupts coronary flow due to the low coronary perfusion pressure^81,89^ and extravascular compressive forces^81,90^, commonly associated with lower systemic arterial compliance and higher arterial resistance^81,91,92^. After TAVR, immediate increase in coronary flow is expected, as a result of increased aortic diastolic pressure (with increased forward pressure gradient at the coronary ostium) and decreased LV end diastolic pressure^81,82^. However, our findings revealed that for patients who undergo TAVR and suffer from PVL, despite the increase of aortic pressure and systemic arterial compliance, there is considerable decrease in coronary flow during diastole. We observed (Figs. 9 and 10) that in the presence of PVL, a considerable portion of the forward flow towards coronary ostium diverges towards the left ventricle, leading to a decreased coronary flow. Furthermore, in agreement with recent studies^93–95^, our results demonstrate the coronary flow is impeded if the distance between stent and coronary ostium is restricted after TAVR (Fig. 9). Such decrease in coronary blood flow is associated with reduced capacity to augment myocardial oxygenation, leading to LV dysfunction, increased apoptosis (which is linked to myocardial fibrosis and is an independent indicator of mortality) and sudden death^81,96–98^.

#### In all patients with PVL following TAVR: No improvement of coronary flow post-TAVR

Although an increase in coronary flow is expected after AS removal and TAVR implantation^82^, our results showed that for all patients who had PVL following TAVR, a universal reduction of flow occurs during diastole for all coronary branches (Fig. 17). Recent studies suggest that despite the early improvements of systolic flow right after TAVR, coronary diastolic flow might not improve during the long-term (6-month) follow up^99^. Our results show that the coronary diastolic flow recovery is even worse for patients with PVL following TAVR. Reduced flow in coronaries could affect the outcomes of revascularization and might play a role in the pathophysiological abnormalities leading to heart failure or increased risk of cardiovascular death^96^.

### Sinus and neo-sinus washout after TAVR may be impaired in presence of paravalvular leak

TAVR can disturb the vortical structures inside the Valsalva sinuses, which are essential for the washout of sinus flow, assisting the smooth closure of the valve and providing flow to the coronary arteries during diastole^100–102^. While the sinus and neo-sinus washout efficacy of different transcatheter heart valves are still under debate^103^, our findings demonstrate that in addition to the TAVR influence on the aortic root morphology, PVL exacerbates the washout mechanism for the sinus and neo-sinus regions. We observed that the PVL jet substantially drains the flow from the sinus and neo-sinus regions, leading to pull the vortices out of the neo-sinus regions. Consequently, the vortices in the neo-sinus regions have less power to transfer the flow out of the leaflet roots. In addition, our results showed that NCC neo-sinus could be influenced the most by PVL, however, the LCC and RCC neo-sinuses irregular washout amplification depends on the severity of PVL and its location. The inefficient sinus and neo-sinus washout favors the thrombotic events after TAVR^86,100^.

### Subclinical leaflet thrombosis risk and hypo-attenuated leaflet thickening may be exacerbated in presence of paravalvular leak

The clinical understanding of leaflet thrombosis after TAVR is limited and little is known about the correlation of leaflet thrombosis with local hemodynamics^104,105^. Hypo-attenuating leaflet thickening (HALT) is a thin layer of thrombus covering the aortic side of the leaflets due to subclinical leaflet thrombosis^106^. Several risk factors have been reported for thrombosis after TAVR, including reduced valve durability, restricted leaflet motion and stroke^107–110^. In addition to the agreement between our findings and previous studies^105,109,111^ regarding TAVR stent morphology effect on blood stasis, we found that the PVL exacerbates the blood stasis volume in the neo-sinus regions nonuniformly and asymmetrical with respect to the valve center. While it has been reported that flow stasis risk is almost equal for LCC, RCC and NCC neo-sinuses^109^, our results revealed that not only PVL increases the blood stasis and thrombus risk in neo-sinus regions globally, but also is different for each neo-sinus depending on the PVL severity and location. We observed that the NCC neo-sinus is more prone to be exposed to stagnant flow and is therefore at higher risk of leaflet thrombosis than LCC and RCC.

### PVL exacerbated aortic root and coronary arteries hemodynamics (local)

The jets emerging from the PVL orifice substantially alters the vortical structure in the aortic root, creating disturbed flow, leading to very high shear stress at the aortic root wall. Our results demonstrate that PVL amplifies non physiological flow patterns, and consequently increases TAWSS after TAVR, especially around the leakage location. The local abnormalities in WSS are thought to stimulate aneurysm formation or lead to progressive dilation of aortic root and ascending aorta^88,112^.

On the other hand, our findings show that PVL leads to a significantly lower shear stress at the coronary walls due to the decreased blood supply during diastole after TAVR. This makes the coronary arteries susceptible to atherosclerosis, due to the low wall shear stress-induced inflammatory activation of endothelium mainly at the inner bend of curved arteries, ostia of branches and lateral walls of bifurcations^76,79^. Therefore, the decreased wall shear stress is associated with enlargement of plaque area, increased plaque eccentricity and reduced vessel area^76,113–115^.

#### In all patients with PVL following TAVR: increased shear stress of aortic root and decreased shear stress of coronary arteries

For all patients in our study, PVL following TAVR exacerbated the shear stress during diastole (calculated through TAWSS) at aortic root and coronary arteries. TAWSS universally reduced in all branches of coronary arteries for all patients, and in contrast, significant increase of TAWSS was observed at the aortic root and around the leakage cite (Fig. 15). While the correlation of decreased shear stress at the coronaries with increased risk of plaque progression has been shown previously^76,79^, recent clinical studies also suggest that increased WSS at the aortic root could lead to ascending aorta dilation and rupture^116,117^.

### PVL worsened the left ventricular hemodynamics (global)

Our results showed that moderate to severe PVL increased the burden on the LV for all patients. Despite the LV pressure reduction and increase in aortic pressure post-TAVR, LV workload increased for all patients as a result of volume overload following PVL. Therefore, PVL following the malpositioning of TAVR causes an overloaded LV, resulting in faster cardiac tissue damage and LV dysfunction. In addition, an overloaded LV may lead to other valvular diseases such as mitral regurgitation or exacerbate the existed regurgitation for patients with mixed valvular disease who receive TAVR^12,19,21^.

As shown in Fig. 16, our results showed that for all patients, the overall decrease in end diastolic pressure and increase of ascending aorta pressured lead to improved perfusion pressure. Moreover, systemic arterial compliance was improved for most of the patients (SAC reduced only for one patient (Fig. 16)). However, PVL following TAVR lead to an increased workload for most patients (LV load reduced only for one patient (Fig. 16)). The increased workload contributes to progressive myocardial fibrosis and eventually myocardial dysfunction^118,119^.

### Limitations of current clinical imaging modalities to capture coronary flow

Over the past decade, the use of medical imaging has drastically increased. In spite of amazing advancements in medical imaging, *medical imaging on its own cannot quantify local and global hemodynamics in coronaries*^120,121^. As the need for patient-specific diagnostic methods continues to be studied, understanding the strengths and limitations of imaging modalities for coronaries is critical toward creating precise diagnostic tools:(1) *Computed tomography coronary angiography (CTCA)*: CTCA has a high spatial resolution allowing for visualization of coronary plaque and stenosis geometry^22,122^. However, CTCA suffers from temporal resolution challenges and requires the use of radiation, which is associated with health concerns especially in younger patients who need several scans throughout their lifetime^123^. CTCA does not provide any local and global hemodynamics measurements; (2) *4D flow magnetic resonance imaging (4D flow MRI):* 4D flow MRI is an emerging technology to allow local hemodynamic assessment in valvular, vascular and ventricular diseases. However, use of 4D flow MRI is limited in patients with implanted medical devices as they remain a major risk during the examination. Moreover, complete and thorough analysis of local hemodynamics in coronaries is not possible^22^, due to the limited temporal resolution (4-D flow MRI has relatively high spatial resolution but lower temporal resolution (20 ms highest)). 4D flow MRI could not provide global hemodynamics; (3) *Doppler echocardiography (DE):* DE does not have the ability to quantify local hemodynamics through coronaries as well global hemodynamics^124^ (4) *Ultrafast ultrasound:* Ultrafast ultrasound is an alternative option for DE, as it can image the heart at a rate of a thousand images per second^125^. Recently, ultrafast ultrasound has been combined with coronary Doppler imaging for quantification of local hemodynamics, which has aided in the diagnosis of PVR^125^. However, it has a limited imaging depth of 45 mm and cannot provide absolute quantification of flowrate for adult patients with coronary disease^125^ (5) *Intravascular Ultrasound (IVUS) & Optical Coherence Tomography (OCT):* IVUS and OCT provide information regarding severity of calcification, plaque morphology, and accurate vessel size^126,127^. However, none of them can provide local and global hemodynamics^128^. (6) *Coronary angiography*: Coronary angiography involves the transmission of a catheter into the coronary artery and the injection of a contrast medium into the bloodstream which is then viewed under X-ray examination^129^. Despite the benefits, coronary angiography is a highly invasive procedure that has shown to poorly measure FFR and evaluate the hemodynamic significance in coronaries^129^.

### Limitations of current computational modeling to capture coronary flow

A clinically useful computational diagnostic framework should evaluate both global and local hemodynamics by quantifying three main requirements: (1) metrics of circulatory function (global hemodynamics), (2) metrics of cardiac function (global hemodynamics) and (3) Cardiac fluid dynamics (local hemodynamics)^18–21,33,130^. Few studies have been conducted to investigate the hemodynamic complexities after TAVR due to the presence of PVL using computational fluid dynamics (CFD)^67–69,131–133^. However, since: (1) patient-specific boundary conditions were not used; (2) hemodynamic validation was not performed; and (3) coronary arteries were excluded from the computational domain, the models developed in these studies did not satisfy the three requirements outlined in the Introduction^67–69,131–133^. In addition, several studies have recently used FSI as a promising tool for coronary arteries exclusively, since it allows consideration of the interactions of artery wall elastic behavior and blood flow mechanics, thus demonstrating its worth as a more realistic tool for numerical modelling of coronary arteries^58,134–141^. While only a few numbers of these studies^58^ coupled lumped parameter model-based boundary conditions with FSI modelling, the lumped-parameter models were not patient-specific. Moreover, all of these studies have excluded the aortic root and sinus geometry from the computational domain^58,134–140^, and most of these studies have used simplified and idealized geometries of coronaries^137,138,142,143^. Exclusion of the aortic sinus at the upstream or using idealized geometry for coronaries could significantly affect the flow structure.

In this study, the requirements mentioned in the Introduction and Discussions have been examined in our study to evaluate the influence of TAVR on coronary arteries and the aortic root, when complications such as PVL or misalignment exist. In summary, our study showed that TAVR removed the aortic valve obstruction during ejection, reduced aortic valve pressure gradient and increased ejection fraction for all patients. However, considering the local flow parameters and cardiac function, all patients had adverse events after TAVR and are at high risk of heart failure. Therefore, despite the improvements of global circulatory function and clinical parameters, our results illustrating the details of local hemodynamics in these patients could partially explain how complications of TAVR could adversely increase the risk of thrombosis at aortic root and neo-sinus region of the valve leaflets, as well as plaque progression inside coronary arteries and subsequent long-term complications.

## Conclusions

An optimal TAVR strategy is patient-specific, and there are varying factors that impacts the coronary hemodynamics including the global hemodynamic and circulatory system adaptation to post-TAVR environment, aortic root and aortic valve anatomical characteristics, coronary geometry, valve to coronary distance and PVL. The optimal stirring flow towards coronary arteries is diverged towards ventricle in presence of PVL and is associated with increased myocardium workload followed by progressive myocardial fibrosis and eventually myocardial dysfunction. The findings of this study suggests that exceptional consideration should be paid to the patients with paravalvular leakage after TAVR, as these patients are at higher risk of reduced coronary flow with reduced capacity to augment myocardial oxygenation, increased workload, leaflet thrombosis, plaque progression and future CAD. These complications are often asymptomatic and can lead to serious health conditions in the future and may have gone undetected if a hemodynamic assessment was not done. The scarcity of clinical trial data for complex dual pathology (CAD and AS) for the patient who undergo TAVR has urged surgeons to decide for revascularization on a case-by-case basis until further trial data. This makes the clinical endpoint and the decision for revascularization very subjective. Patient-specific computational simulations can predict the risk of post-TAVR complications such as PVL and leaflet thrombosis and their impacts on coronary hemodynamics to guide the surgeons for optimal intervention planning. The developed framework in this work is just such a tool to improve the clinical outcomes and guiding interventions for patients who receive TAVR and might be at risk of CAD over the course of time^144^.

## Limitations

This study was performed and validated on 6 patients who underwent TAVR in both pre- and post- intervention states (12 cases). Future studies must consider further validation of the computational framework in a large population of AS patients in both pre- and post-intervention states, however, our results in this study demonstrate the ability of the framework to track changes in both cardiac, and vascular states. One limitation in our 3D FSI simulation is modelling only the diastole phase with TAV to be rigidly closed. It is important to note that PVL occurs only during the diastole and focusing only on diastole phase allows to simplify the simulation and reduce computational challenges and costs^67,68,71^. However, the good agreement between the FSI simulation and DE velocity data showed that this limitation does not affect the conclusions of this study. Another limitation in this study was assuming the coronary arteries fixed without the movements caused by the beating heart. However, some studies suggest that vessels dynamic motions might have negligible impacts on some parameters such as TAWSS^37^. Our computational framework is currently developed based on 6 cases and the inclusion of more cases will aid in improving the results with broader validations that could eventually be linked to patient’s outcomes.

## Supplementary Information

Supplementary Information.

## Data Availability

The codes and the optimization algorithm are available from the correspondence author upon request.

## References

[1] Coylewright M, Forrest JK, McCabe JM, Nazif TM (2020). TAVR in low-risk patients. J. Am. Coll. Cardiol..

[2] Waksman R (2018). Transcatheter aortic valve replacement in low-risk patients with symptomatic severe aortic stenosis. J. Am. Coll. Cardiol..

[3] Shah S (2019). Characteristics and longer-term outcomes of paravalvular leak after aortic and mitral valve surgery. J. Thorac. Cardiovasc. Surg..

[4] Fanous EJ (2020). Paravalvular leak assessment: Challenges in assessing severity and interventional approaches. Curr. Cardiol. Rep..

[5] Pibarot P, Hahn RT, Weissman NJ, Monaghan MJ (2015). Assessment of paravalvular regurgitation following TAVR: A proposal of unifying grading scheme. JACC Cardiovasc. Imaging.

[6] Forrestal BJ (2020). Risk of coronary obstruction and feasibility of coronary access after repeat transcatheter aortic valve replacement with the self-expanding Evolut valve. Circ. Cardiovasc. Interv..

[7] Valvo R, Costa G, Barbanti M (2019). How to avoid coronary occlusion during TAVR valve-in-valve procedures. Front. Cardiovasc. Med..

[8] Taylor CA, Steinman DA (2010). Image-based modeling of blood flow and vessel wall dynamics: Applications, methods and future directions. Ann. Biomed. Eng..

[9] Siebes M, Ventikos Y (2010). The role of biofluid mechanics in the assessment of clinical and pathological observations. Ann. Biomed. Eng..

[10] Anvari, S., Nambiar, S., Pang, J. & Maftoon, N. Computational models and simulations of cancer metastasis. *Arch. Comput. Methods Eng.* (2021).

[11] Lieber BB, Siebes M, Yamaguchi T (2005). Correlation of hemodynamic events with clinical and pathological observations. Ann. Biomed. Eng..

[12] Keshavarz-Motamed Z (2020). Mixed Valvular disease following transcatheter aortic valve replacement: quantification and systematic differentiation using clinical measurements and image-based patient-specific in Silico modeling. J. Am. Heart Assoc..

[13] Pibarot P, Dumesnil JG (2007). Assessment of aortic stenosis severity: check the valve but don’t forget the arteries!. Heart.

[14] Ben-Assa E (2019). Ventricular stroke work and vascular impedance refine the characterization of patients with aortic stenosis. Sci. Transl. Med..

[15] Antonini-Canterin F (2013). The ventricular-arterial coupling: From basic pathophysiology to clinical application in the echocardiography laboratory. J. Cardiovasc. Echography.

[16] Ikonomidis I (2019). The role of ventricular–arterial coupling in cardiac disease and heart failure: Assessment, clinical implications and therapeutic interventions. A consensus document of the European Society of Cardiology Working Group on Aorta & Peripheral Vascular Diseases, European Association of Cardiovascular Imaging, and Heart Failure Association. Eur. J. Heart Fail..

[17] Keshavarz-Motamed Z, Motamed PK, Maftoon N (2015). Non-invasive determination of aortic valve trans-catheter pressure gradient: an analytical model. Med. Eng. Phys..

[18] Khodaei S (2021). Towards a non-invasive computational diagnostic framework for personalized cardiology of transcatheter aortic valve replacement in interactions with complex valvular, ventricular and vascular disease. Int. J. Mech. Sci..

[19] Khodaei S (2021). Personalized intervention cardiology with transcatheter aortic valve replacement made possible with a non-invasive monitoring and diagnostic framework. Sci. Rep..

[20] Baiocchi M (2021). Effects of choice of medical imaging modalities on a non-invasive diagnostic and monitoring computational framework for patients with complex Valvular, vascular, and ventricular diseases who undergo Transcatheter aortic valve replacement. Front. Bioeng. Biotechnol..

[21] Keshavarz-Motamed Z (2020). A diagnostic, monitoring, and predictive tool for patients with complex valvular, vascular and ventricular diseases. Sci. Rep..

[22] Dewey M (2020). Clinical quantitative cardiac imaging for the assessment of myocardial ischaemia. Nat. Rev. Cardiol..

[23] Johnston CM, Krafft AJ, Russe MF, Rog-Zielinska EA (2018). A new look at the heart—novel imaging techniques. Herzschrittmachertherapie Elektrophys..

[24] Adamson PD, Newby DE (2019). Non-invasive imaging of the coronary arteries. Eur. Heart J..

[25] Keshavarz-Motamed Z, Garcia J, Pibarot P, Larose E, Kadem L (2011). Modeling the impact of concomitant aortic stenosis and coarctation of the aorta on left ventricular workload. J. Biomech..

[26] Keshavarz-Motamed Z (2015). The role of aortic compliance in determination of coarctation severity: Lumped parameter modeling, in vitro study and clinical evaluation. J. Biomech..

[27] Keshavarz-Motamed Z (2014). Effect of coarctation of the aorta and bicuspid aortic valve on flow dynamics and turbulence in the aorta using particle image velocimetry. Exp. Fluids.

[28] Keshavarz-Motamed Z (2012). A new approach for the evaluation of the severity of coarctation of the aorta using Doppler velocity index and effective orifice area: In vitro validation and clinical implications. J. Biomech..

[29] Benevento E, Djebbari A, Keshavarz-Motamed Z, Cecere R, Kadem L (2015). Hemodynamic changes following aortic valve bypass: A mathematical approach. PLoS ONE.

[30] Sadeghi R, Tomka N, Khodaei S, Daeian M, Gandhi K, Garcia J, Keshavarz-Motamed Z (2022). Impact of extraanatomical bypass on coarctation fluid dynamics using patient-specific lumped parameter and Lattice Boltzmann modeling. Nat. Sci. Rep..

[31] Asaadi, M. *et al*. On left ventricle stroke work efficiency in children with moderate aortic valve regurgitation or moderate aortic valve stenosis. *Pediatr. Cardiol.* 1-9 (2021). 10.1007/s00246-021-02690-2 34357415

[32] Keshavarz-Motamed Z (2016). Elimination of transcoarctation pressure gradients has no impact on left ventricular function or aortic shear stress after intervention in patients with mild coarctation. JACC Cardiovasc. Interv..

[33] Sadeghi R, Khodaei S, Ganame J, Keshavarz-Motamed Z (2020). Towards non-invasive computational-mechanics and imaging-based diagnostic framework for personalized cardiology for coarctation. Sci. Rep..

[34] Keshavarz-Motamed Z (2014). Non-invasive determination of left ventricular workload in patients with aortic stenosis using magnetic resonance imaging and doppler echocardiography. PLoS ONE.

[35] Sadeghi R, Gasner N, Khodaei S, Garcia J, Keshavarz-Motamed Z (2022). Impact of mixed valvular disease on coarctation hemodynamics using patient-specific lumped parameter and Lattice Boltzmann modeling. Int. J. Mech. Sci..

[36] Sadeghi R (2022). Reducing morbidity and mortality in patients with coarctation requires systematic differentiation of impacts of mixed valvular disease on coarctation hemodynamics. J. Am. Heart Assoc..

[37] Tanné D, Kadem L, Rieu R, Pibarot P (2008). Hemodynamic impact of mitral prosthesis-patient mismatch on pulmonary hypertension: an in silico study. J. Appl. Physiol. Bethesda Md.

[38] Stergiopulos N, Meister JJ, Westerhof N (1996). Determinants of stroke volume and systolic and diastolic aortic pressure. Am. J. Physiol.-Heart Circ. Physiol..

[39] Mynard JP, Davidson MR, Penny DJ, Smolich JJ (2012). A simple, versatile valve model for use in lumped parameter and one-dimensional cardiovascular models. Int. J. Numer. Methods Biomed. Eng..

[40] Kim HJ (2010). Patient-specific modeling of blood flow and pressure in human coronary arteries. Ann. Biomed. Eng..

[41] Taylor CA, Fonte TA, Min JK (2013). Computational fluid dynamics applied to cardiac computed tomography for noninvasive quantification of fractional flow reserve: Scientific basis. J. Am. Coll. Cardiol..

[42] Sankaran S (2012). Patient-specific multiscale modeling of blood flow for coronary artery bypass graft surgery. Ann. Biomed. Eng..

[43] Zhou Y, Kassab GS, Molloi S (1999). On the design of the coronary arterial tree: A generalization of Murray’s law. Phys. Med. Biol..

[44] Coogan JS, Humphrey JD, Figueroa CA (2013). Computational simulations of hemodynamic changes within thoracic, coronary, and cerebral arteries following early wall remodeling in response to distal aortic coarctation. Biomech. Model. Mechanobiol..

[45] Seemann F (2019). Noninvasive quantification of pressure-volume loops from brachial pressure and cardiovascular magnetic resonance. Circ. Cardiovasc. Imaging.

[46] Gleason WL, Braunwald E (1962). Studies on the first derivative of the ventricular pressure pulse in man. J. Clin. Invest..

[47] Van de Werf F (1984). Diastolic properties of the left ventricle in normal adults and in patients with third heart sounds. Circulation.

[48] Kass DA, Midei M, Graves W, Brinker JA, Maughan WL (1988). Use of a conductance (volume) catheter and transient inferior vena caval occlusion for rapid determination of pressure-volume relationships in man. Cathet. Cardiovasc. Diagn..

[49] Takeuchi M, Odake M, Takaoka H, Hayashi Y, Yokoyama M (1992). Comparison between preload recruitable stroke work and the end-systolic pressure-volume relationship in man. Eur. Heart J..

[50] Senzaki H, Chen CH, Kass DA (1996). Single-beat estimation of end-systolic pressure-volume relation in humans: A new method with the potential for noninvasive application. Circulation.

[51] Brown KA, Ditchey RV (1988). Human right ventricular end-systolic pressure-volume relation defined by maximal elastance. Circulation.

[52] Dell’Italia LJ, Walsh RA (1988). Application of a time varying elastance model to right ventricular performance in man. Cardiovasc. Res..

[53] Maniar HS (2003). Impact of pericardial restraint on right atrial mechanics during acute right ventricular pressure load. Am. J. Physiol. Heart Circ. Physiol..

[54] Liang F, Takagi S, Himeno R, Liu H (2009). Multi-scale modeling of the human cardiovascular system with applications to aortic valvular and arterial stenoses. Med. Biol. Eng. Comput..

[55] Yin M, Yazdani A, Karniadakis GE (2019). One-dimensional modeling of fractional flow reserve in coronary artery disease: Uncertainty quantification and Bayesian optimization. Comput. Methods Appl. Mech. Eng..

[56] Li B, Wang W, Mao B, Liu Y (2019). A method to personalize the lumped parameter model of coronary artery. Int. J. Comput. Methods.

[57] Fossan FE (2018). Uncertainty quantification and sensitivity analysis for computational FFR estimation in stable coronary artery disease. Cardiovasc. Eng. Technol..

[58] Tajeddini F (2020). High precision invasive FFR, low-cost invasive iFR, or non-invasive CFR?: Optimum assessment of coronary artery stenosis based on the patient-specific computational models. Int. J. Numer. Methods Biomed. Eng..

[59] Mantero S, Pietrabissa R, Fumero R (1992). The coronary bed and its role in the cardiovascular system: a review and an introductory single-branch model. J. Biomed. Eng..

[60] Razminia M (2004). Validation of a new formula for mean arterial pressure calculation: The new formula is superior to the standard formula. Catheter. Cardiovasc. Interv..

[61] Garcia D (2009). Impairment of coronary flow reserve in aortic stenosis. J. Appl. Physiol..

[62] Ofili EO (1995). Differential characterization of blood flow, velocity, and vascular resistance between proximal and distal normal epicardial human coronary arteries: analysis by intracoronary Doppler spectral flow velocity. Am. Heart J..

[63] Stergiopulos N, Segers P, Westerhof N (1999). Use of pulse pressure method for estimating total arterial compliance in vivo. Am. J. Physiol.-Heart Circ. Physiol..

[64] Weller HG, Tabor G, Jasak H, Fureby C (1998). A tensorial approach to computational continuum mechanics using object-oriented techniques. Comput. Phys..

[65] Singh-Gryzbon S (2020). Influence of patient-specific characteristics on transcatheter heart valve neo-sinus flow: An in silico study. Ann. Biomed. Eng..

[66] Hatoum H (2021). Predictive model for thrombus formation after transcatheter valve replacement. Cardiovasc. Eng. Technol..

[67] de Jaegere P (2016). Patient-specific computer modeling to predict aortic regurgitation after transcatheter aortic valve replacement. JACC Cardiovasc. Interv..

[68] Mao W, Wang Q, Kodali S, Sun W (2018). Numerical parametric study of Paravalvular leak following a Transcatheter aortic valve deployment into a patient-specific aortic root. J. Biomech. Eng..

[69] Lavon K (2019). Biomechanical modeling of transcatheter aortic valve replacement in a stenotic bicuspid aortic valve: Deployments and paravalvular leakage. Med. Biol. Eng. Comput..

[70] Azadani AN (2009). Energy loss due to paravalvular leak with transcatheter aortic valve implantation. Ann. Thorac. Surg..

[71] Bianchi M (2019). Patient-specific simulation of transcatheter aortic valve replacement: impact of deployment options on paravalvular leakage. Biomech. Model. Mechanobiol..

[72] Basri AA (2020). Fluid structure interaction on paravalvular leakage of transcatheter aortic valve implantation related to aortic stenosis: A patient-specific case. Comput. Math. Methods Med..

[73] Rocatello G (2018). Patient-specific computer simulation to elucidate the role of contact pressure in the development of new conduction abnormalities after catheter-based implantation of a self-expanding aortic valve. Circ. Cardiovasc. Interv..

[74] Esmailie F (2022). Biomechanics of transcatheter aortic valve replacement complications and computational predictive modeling. Struct. Heart.

[75] Madukauwa-David ID (2020). An evaluation of the influence of coronary flow on transcatheter heart valve neo-sinus flow stasis. Ann. Biomed. Eng..

[76] Gijsen F (2019). Expert recommendations on the assessment of wall shear stress in human coronary arteries: existing methodologies, technical considerations, and clinical applications. Eur. Heart J..

[77] Dolan JM, Kolega J, Meng H (2013). High wall shear stress and spatial gradients in vascular pathology: A review. Ann. Biomed. Eng..

[78] Ferrara A, Pandolfi A (2008). Numerical modelling of fracture in human arteries. Comput. Methods Biomech. Biomed. Engin..

[79] Cameron JN (2020). Exploring the relationship between biomechanical stresses and coronary atherosclerosis. Atherosclerosis.

[80] Bahlmann E (2019). Low systemic arterial compliance is associated with increased cardiovascular morbidity and mortality in aortic valve stenosis. Heart.

[81] McConkey HZR (2019). Coronary microcirculation in aortic stenosis: A physiological hornets’ nest. Circ. Cardiovasc. Interv..

[82] Ben-Dor I (2014). Coronary blood flow in patients with severe aortic stenosis before and after transcatheter aortic valve implantation. Am. J. Cardiol..

[83] Faroux L (2019). Coronary artery disease and transcatheter aortic valve replacement: JACC state-of-the-art review. J. Am. Coll. Cardiol..

[84] Scarsini R (2020). Long-term variations of FFR and iFR after transcatheter aortic valve implantation. Int. J. Cardiol..

[85] Calderan J, Mao W, Sirois E, Sun W (2016). Development of an in vitro model to characterize the effects of Transcatheter aortic valve on coronary artery flow. Artif. Organs.

[86] Pott D (2021). Hemodynamics inside the neo- and native sinus after TAVR: Effects of implant depth and cardiac output on flow field and coronary flow. Artif. Organs.

[87] Iwata S, Inano C, Ozaki M (2020). Perpendicular and turbulent flow after aortic valve replacement: Paravalvular or transvalvular leakage? – A case report. J. Cardiothorac. Surg..

[88] Farag ES (2019). Transcatheter aortic valve replacement alters ascending aortic blood flow and wall shear stress patterns: A 4D flow MRI comparison with age-matched, elderly controls. Eur. Radiol..

[89] Crea F, Camici PG, Bairey Merz CN (2014). Coronary microvascular dysfunction: An update. Eur. Heart J..

[90] Dunn RB, Griggs DM (1983). Ventricular filling pressure as a determinant of coronary blood flow during ischemia. Am. J. Physiol..

[91] Pibarot P, Dumesnil JG (2012). Low-flow, low-gradient aortic stenosis with normal and depressed left ventricular ejection fraction. J. Am. Coll. Cardiol..

[92] Tiwari N, Madan N (2018). Hypertension and transcatheter aortic valve replacement: parallel or series?. Integr. Blood Press. Control.

[93] Nai Fovino L (2020). Coronary angiography after transcatheter aortic valve replacement (TAVR) to evaluate the risk of coronary access impairment after TAVR-in-TAVR. J. Am. Heart Assoc..

[94] Oh J-H (2021). Distance between valvular leaflet and coronary ostium predicting risk of coronary obstruction during TAVR. IJC Heart Vasc..

[95] Heitkemper M (2021). Simple 2-dimensional anatomic model to predict the risk of coronary obstruction during transcatheter aortic valve replacement. J. Thorac. Cardiovasc. Surg..

[96] Lester SJ, Heilbron B, Gin K, Dodek A, Jue J (1998). The natural history and rate of progression of aortic stenosis. Chest.

[97] Vesey AT, Esson G, Chin C, Dweck M, Newby D (2015). Detection of cardiac fibrosis and cell death in patients with aortic stenosis. J. Am. Coll. Cardiol..

[98] Dweck MR (2011). Midwall fibrosis is an independent predictor of mortality in patients with aortic stenosis. J. Am. Coll. Cardiol..

[99] Vendrik J (2020). Long-term effects of transcatheter aortic valve implantation on coronary hemodynamics in patients with concomitant coronary artery disease and severe aortic stenosis. J. Am. Heart Assoc..

[100] Toninato R, Salmon J, Susin FM, Ducci A, Burriesci G (2016). Physiological vortices in the sinuses of Valsalva: An in vitro approach for bio-prosthetic valves. J. Biomech..

[101] Moore BL (2014). Influence of Anatomic Valve Conditions and Coronary Flow on Aortic Sinus Hemodynamics.

[102] Kaneko T (2021). Flow in the aortic sinus after valve-in-valve TAVR. JACC Cardiovasc. Interv..

[103] Hatoum H (2021). Neosinus and sinus flow after self-expanding and balloon-expandable transcatheter aortic valve replacement. JACC Cardiovasc. Interv..

[104] Ryo Y (2019). Early and late leaflet thrombosis after transcatheter aortic valve replacement. Circ. Cardiovasc. Interv..

[105] Midha PA (2017). The fluid mechanics of transcatheter heart valve leaflet thrombosis in the neosinus. Circulation.

[106] Rosseel L, De Backer O, Søndergaard L (2018). Clinical valve thrombosis and subclinical leaflet thrombosis in transcatheter aortic heart valves: clinical manifestations, diagnosis, and treatment. Precis. Clin. Med..

[107] Jose J (2017). Clinical bioprosthetic heart valve thrombosis after transcatheter aortic valve replacement. JACC Cardiovasc. Interv..

[108] Sellers SL (2019). Transcatheter aortic heart valves. JACC Cardiovasc. Imaging.

[109] Trusty PM (2020). The role of flow stasis in transcatheter aortic valve leaflet thrombosis. J. Thorac. Cardiovasc. Surg..

[110] Brown RA (2020). Subclinical leaflet thrombosis post transcatheter aortic valve replacement – an update for 2020. Struct. Heart.

[111] Raghav V (2019). Three-dimensional extent of flow stagnation in transcatheter heart valves. J. R. Soc. Interface.

[112] Trauzeddel RF (2016). Blood flow characteristics in the ascending aorta after TAVI compared to surgical aortic valve replacement. Int. J. Cardiovasc. Imaging.

[113] Samady H (2011). Coronary artery wall shear stress is associated with progression and transformation of atherosclerotic plaque and arterial remodeling in patients with coronary artery disease. Circulation.

[114] Eshtehardi P (2012). Association of coronary wall shear stress with atherosclerotic plaque burden, composition, and distribution in patients with coronary artery disease. J. Am. Heart Assoc..

[115] Papafaklis MI (2015). Effect of the local hemodynamic environment on the de novo development and progression of eccentric coronary atherosclerosis in humans: Insights from PREDICTION. Atherosclerosis.

[116] Soulat G (2022). Association of regional wall shear stress and progressive ascending aorta dilation in bicuspid aortic valve. JACC Cardiovasc. Imaging.

[117] Guala A (2022). Wall shear stress predicts aortic dilation in patients with bicuspid aortic valve. JACC Cardiovasc. Imaging.

[118] Rader F, Sachdev E, Arsanjani R, Siegel RJ (2015). Left ventricular hypertrophy in valvular aortic stenosis: Mechanisms and clinical implications. Am. J. Med..

[119] Kampaktsis PN (2021). Impact of paravalvular leak on left ventricular remodeling and global longitudinal strain 1 year after transcatheter aortic valve replacement. Future Cardiol..

[120] Di Carli Marcelo F, Geva T, Davidoff R (2016). The future of cardiovascular imaging. Circulation.

[121] Kadem M, Garber L, Abdelkhalek M, Al-Khazraji BK, Keshavarz-Motamed Z (2022). Hemodynamic modeling, medical imaging, and machine learning and their applications to cardiovascular interventions. IEEE Rev. Biomed. Eng..

[122] Fairbairn TA (2020). Sex differences in coronary computed tomography angiography-derived fractional flow reserve. JACC Cardiovasc. Imaging.

[123] Henein MY, Vancheri S, Bajraktari G, Vancheri F (2020). Coronary atherosclerosis imaging. Diagnostics.

[124] Zagatina A (2018). Role of coronary flow velocity in predicting adverse outcome in clinical practice. Ultrasound Med. Biol..

[125] Maresca D (2018). Noninvasive imaging of the coronary vasculature using ultrafast ultrasound. JACC Cardiovasc. Imaging.

[126] Shammas NW (2019). The role of precise imaging with intravascular ultrasound in coronary and peripheral interventions. Vasc. Health Risk Manag..

[127] Lee CH, Hur S-H (2019). Optimization of percutaneous coronary intervention using optical coherence tomography. Korean Circ. J..

[128] Darmoch F (2020). Intravascular ultrasound imaging-guided versus coronary angiography-guided percutaneous coronary intervention: A systematic review and meta-analysis. J. Am. Heart Assoc..

[129] Uus A (2016). Patient-Specific Blood Flow Modelling in Diagnosis of Coronary Artery Disease.

[130] Garber, L., Khodaei, S. & Keshavarz-Motamed, Z. The critical role of lumped parameter models in patient-specific cardiovascular simulations. *Arch. Comput. Methods Eng.* 1-24 (2022).

[131] Rocatello G (2019). The impact of size and position of a mechanical expandable transcatheter aortic valve: Novel insights through computational modelling and simulation. J Cardiovasc. Transl. Res..

[132] Luraghi G (2019). On the modeling of patient-specific transcatheter aortic valve replacement: A fluid-structure interaction approach. Cardiovasc. Eng. Technol..

[133] Schultz C (2016). Patient-specific image-based computer simulation for theprediction of valve morphology and calcium displacement after TAVI with the Medtronic CoreValve and the Edwards SAPIEN valve. EuroIntervention J. Eur. Collab. Work. Group Interv. Cardiol. Eur. Soc. Cardiol..

[134] Pakravan HA, Saidi MS, Firoozabadi B (2017). A multiscale approach for determining the morphology of endothelial cells at a coronary artery. Int. J. Numer. Methods Biomed. Eng..

[135] Guo X (2018). Combining IVUS and optical coherence tomography for more accurate coronary cap thickness quantification and stress/strain calculations: A patient-specific three-dimensional fluid-structure interaction modelling approach. J. Biomech. Eng..

[136] Guo X (2017). Quantify patient-specific coronary material property and its impact on stress/strain calculations using in vivo IVUS data and 3D FSI models: A pilot study. Biomech. Model. Mechanobiol..

[137] Jahromi R, Pakravan HA, Saidi MS, Firoozabadi B (2019). Primary stenosis progression versus secondary stenosis formation in the left coronary bifurcation: A mechanical point of view. Biocybern. Biomed. Eng..

[138] Gholipour A, Ghayesh MH, Zander A, Mahajan R (2018). Three-dimensional biomechanics of coronary arteries. Int. J. Eng. Sci..

[139] Bukač M, Čanić S, Tambača J, Wang Y (2019). Fluid–structure interaction between pulsatile blood flow and a curved stented coronary artery on a beating heart: A four stent computational study. Comput. Methods Appl. Mech. Eng..

[140] Wang L (2021). Optical coherence tomography-based patient-specific residual multi-thrombus coronary plaque models with fluid–structure interaction for better treatment decisions: A biomechanical modeling case Study. J. Biomech. Eng..

[141] Shen J, Faruqi AH, Jiang Y, Maftoon N (2021). Mathematical reconstruction of patient specific vascular networks based on clinical images and global optimization. IEEE Access.

[142] Gholipour A, Ghayesh MH, Zander A (2018). Nonlinear biomechanics of bifurcated atherosclerotic coronary arteries. Int. J. Eng. Sci..

[143] Rabbi MF, Laboni FS, Arafat MT (2020). Computational analysis of the coronary artery hemodynamics with different anatomical variations. Inform. Med. Unlocked.

[144] Keshavarz-Motamed, Z., del Alamo, J. C., Bluestein, D., Edelman, E. R. & Wentzel, J. J. Novel methods to advance diagnostic and treatment value of medical imaging for cardiovascular disease. *Front. Bioeng. Biotechnol*. (Biomechanics section), 1501 (2022). 10.3389/fbioe.2022.987326 PMC9471953 36118589

