# Supplementary material for: Long-term prognostic impact of paravalvular leakage on coronary artery disease requires patient-specific quantification of hemodynamics
Source: Sci Rep. 2022 Dec 9;12:21357. doi: 10.1038/s41598-022-21104-8 (PMC9734172; doi:10.1038/s41598-022-21104-8)
Supplement: Supplementary file 1 — Supplementary Information. [file 41598_2022_21104_MOESM1_ESM.docx]

**Supplemental Material**

## ***2.3. Fluid-structure simulation study***

## *2.3.2.* Governing equations for fluid domain

Blood flow was considered to be incompressible, Newtonian with a density of 1060 kg/m3 and dynamic viscosity of 0.004 Pa·s ^1,2^. Since the Reynolds number is less than 500 inside the coronary arteries without severe stenosis (i.e. the case for the patients in our study), the blood flow is assumed to be laminar^3^. Using 3-D incompressible Navier-Stokes equations in the form of Arbitrary Lagrangian-Eulerian (ALE), the continuity and momentum equations can be described as ^4–6^:

| $\oint_{s} (n.V)ds=0$ | (24) |
| --- | --- |
| $\int_{\forall} \frac{\partial V}{\partial t}d\forall+\oint_{s} n.(V-V_{s})Vds=\frac{1}{\rho}\oint_{s} n.[\mu\nabla V]ds-\frac{1}{\rho}\int_{\forall} \nabla pd\forall$ | (25) |

where *n,* $\forall$ , *V, µ*, *P*, *ρ* and *Vs* represent the normal vector to the surface *S,* the volume, fluid velocity, fluid dynamic viscosity, blood pressure, fluid density, and velocity of the surface respectively.

### 2.3.3. Governing equations for solid domain

The coronary artery is comprised of three layers, intima, media, and adventitia, with the center layer, the media, constituting the majority of coronary tissue thickness ^7^. Obtaining the human coronary tissue thickness and material properties are difficult due to the scarcity of human coronary tissue samples. In vivo quantification of material properties is even harder ^8^. Creating an empirical model to describe the behavior of coronary artery has been of great interest with several previous designs ^9–15^. Such in vitro studies, although proven to be reliable, were applied to the arteries after harvesting them from the body. Therefore, the properties and loading conditions in their studies do not accurately replicate the physiological conditions ^16^. There are several studies on solid modelling of the coronary arteries based on clinical in vivo measurements, using IVUS and OCT ^17–22^. However, both of these intracoronary imaging modalities introduce catheter based probes into the coronary artery and therefore, are invasive ^16,20^. Using these techniques would be almost impossible for old patients with TAVR, for whom the coronary artery examination is limited to only non-invasive hemodynamic assessment using computed coronary angiography ^23–25^. Due to these formidable challenges, there is no non-invasive method for obtaining patient specific material properties of coronary artery tissue to date ^26^.

Unlike previous FSI models with an idealized and simplified geometry for the coronary tissue ^27–35^, in this study, an average thickness of 1 mm was extruded from the patient specific fluid domain wall according to the previous clinical measured data for the tissue (solid domain) ^36^. Moreover, we assumed an isotropic neo-Hookean solid model for the coronary tissue ^37–40^. As shown in previous studies, the stiffness of coronary tissue increases significantly for older patients and with coronary artery disease due to complications such as collagen deposition or elastin fragmentation ^15,41–43^. Since all patients in our study were elderly (average of 86 years old) and diagnosed with CAD, a Young’s modulus of 4 MPa was assumed for all patients (Poisson’s ratio for both patients: 0.49; density for both patients: 1000 kg/m^3^) based on previous experiments to consider more realistic behavior of the tissue ^15,41^.

The governing momentum equation of solid domain in terms of initial undeformed configuration can be written as ^44–46^:

| $\int_{\forall_{0}} \rho_{0}\frac{\partial^{2}u}{\partial t^{2}}d\forall_{0}=\oint_{s_{0}} \sigma.(JF^{-T}.n_{0})ds_{0}+\int_{V_{0}} \rho_{0}bdV_{0}$ | (26) |
| --- | --- |

where the subscript 0 denotes the undeformed configuration and u is the displacement vector. F is the deformation gradient tensor, described by *F = I + (∇u)^T^,* where *I* is the second order identity tensor and the Jacobian of the deformation gradient is *J = det[F]*.

| $\sigma=\mu\mathrm{dev}\left[ \bar{b} \right]+\frac{k}{2}( \frac{J^{2}-1}{J} )$ | (27) |
| --- | --- |

For a neo-Hookean solid, σ can be expressed as ^46,47^:

| $\bar{b}=J^{-\frac{2}{3}}F.F^{T}$ | (28) |
| --- | --- |

Where µ is the shear modulus and K is the bulk modulus (related to the Young’s modulus and Poisson’s ratio of material).

### 2.3.4. Fluid-structure interaction (FSI)

The coronary arteries were simulated under pathophysiological flow and pressure conditions with the strong coupling of the fluid and solid solvers. The fluid and solid domains system of equations were solved independently using the partitioned approach and the data was transferred at the FSI interface. Fluid and solid solvers were coupled by satisfying the kinematic and dynamic conditions. The kinematic and dynamic coupling conditions in the simulation process were ^44^:

| $u_{f,i}=u_{s,i}$ | (29) | |
| --- | --- | --- |
| $V_{f,i}=V_{s,i}$ | (30) | |
| $n_{i}.\sigma_{f,i}=n_{i}.\sigma_{s,i}$ | (31) |  |

where subscripts *i, s,* and *f* indicate the interface, solid and fluid regions, respectively. Equation (31) indicates that the forces at the interface must be in equilibrium.

*2.3.5. Grid independence study*

For FSI simulations in the coronary arteries, different studies in the literature reported different acceptable error values between medium and refined mesh for the mesh independency. Several studies attained a reasonable accuracy once the variation of the peak velocity inside coronary was less than 5% ^48–51^. Several other studies considered the flow results to be acceptable with variations of lower than 3% in the average of velocity ^52^, flow rate ^53–55^ or pressure ^53–55^ for mesh independency. In addition, regarding the mesh independency with respect to wall shear stress (WSS), previous studies considered to be acceptable either less than 5% variation in peak (maximum) WSS ^52,56^ or less than 1% variation in the average WSS ^51,57–59^.

We used an open-source mesh generation software, SALOME, to produce the mesh for all models ^60^. The number of elements for FSI simulation was optimized through the examination of spatial mesh resolution. We had performed a grid convergence analysis and the spatial resolution for our models ranged between 0.2 mm to 1 mm (unstructured tetrahedral elements with refined mesh near walls) (See Figures 6 and 7 for a sample of our grid convergence study). With optimized non-orthogonality and skewness values, the mesh deﬁnition for both the fluid and solid domains was considered acceptable when the velocity profiles in successive meshes showed a variation of less than 0.5%, such that this difference was not signiﬁcant. The fluid dynamic mesh was controlled by Laplace mesh motion, which was subsequently influenced by variable diffusivity, to ensure the initial quality of the cells was maintained ^4,61^. Mesh at the interface of the fluid and solid domains was not conformal. Consequently, following the face-interpolation and vertex-interpolation procedures, interpolation could be performed between the fluid and solid boundaries ^4^. Indeed, we observed 0.5% variation as the maximum error in our mesh independency study in the entire velocity domain. At any other point in the domain and at any instance during diastole, the maximum error was less than 0.5% and the variation of average velocity was less than 0.3%. In our study, the maximum WSS variations occurred at the PVL site and was less than 3% (Figure 7) while the variation of the average WSS at the PVL site was less than 1%. In addition, the variation of the maximum value of the Von Mises stress for the coronary arteries (solid domain) was less than 2% (Figure 7) ^48,62^.

Moreover, time step independency had been studied for all models. We found that a maximum Courant number of 0.5 was yielded as the solution marched in time with a time step of 0.00001s. For all simulations investigated in this study, the Courant number was lower than 0.5. This limit improved the accuracy of the numerical solution and reduced numerical dispersion. Convergence was obtained when all residuals reached a value lower than 10^-6^. Temporal discretization and spatial discretization were performed using a second-order Euler backward scheme and a second-order accurate scheme, respectively.

### 2.3.6. Model reconstruction

We segmented and reconstructed the 3-D geometries of the ascending aorta, aortic root, sinotubular junction, sinus of Valsalva, aortic valve and main coronary arteries (proximal RCA, LMCA, proximal LCX and proximal LAD) in patients with aortic stenosis (Pre-TAVR) and Post-TAVR from CT images using ITK-SNAP (version 3.8.0-BETA) ^63^, a 3-D image processing and model generation software package (Figure 4). These 3-D reconstructions of the model were performed based on images taken at the beginning of diastole. The reconstructed 3-D models were then used for FSI simulations during diastole. We used a smoothing procedure for the surfaces to overcome the challenges of computational convergence and stability. The change in the volume due to smoothing was less than 3% in all patients. The post-TAVR CT data was used to extract the exact deployed TAV configuration of the implanted valve for the simulation. In order to consider the details of implanted valve (post-TAVR) and the FSI interface of coronary arteries, four main steps were taken. First, the post-TAVR fluid domain including ascending aorta, aortic root, valve leaflets and coronary arteries were segmented and reconstructed from CT images. Then, the stent for each patient was separately segmented from the CT images considering the elliptical and asymmetrical deployment of the TAVR. The reason to segment the stent separately was the necessity of using different threshold for segmentation of stent in comparison to the tissue. In order to mimic the impermeable skirt seal of the Sapien valve, a skirt was added to the TAV stent in the CFD model^64^. Afterwards, the reconstructed deployed TAV was located in the exact post-TAVR implanted location guided by CT images using following measurements: the TAV distance to aortic wall^65^, TAV distance to left and right ostium^65^, implantation depth^65^, the heights of the left and right coronary arteries from annulus^66^ and TAV tilted angle with respect to ascending aorta^65,67^. Finally, the coronary artery walls (starting from the ostium and ending at coronary outlets) were extruded with a thickness of 1mm to create the solid domain for the coronary arteries^36^.

### 2.3.7.FSI solution and strategy

We used open-source FOAM-Extend ^68^ library to formulate the system of equations governing the FSI problem using the finite volume method (Please see Figure 5 for FSI algorithm flowchart). For both fluid and solid domains, the cell-centered finite volume method was utilized to solve this system of equations ^5,6,45,69^.

For the fluid model, the spatial discretization relied on second-order accurate cell-centred finite volume method whilst the second-order backward Euler scheme was used for numerical integration in time. The segregated PISO algorithm^61,70^ was used to couple pressure and velocity and the final system of discretized equations was solved using a preconditioned Bi-Conjugate Gradient method ^71^.

In order to consolidate the discretization of the temporal terms between the fluid and solid discretization methods and stabilize the numerical model ^72^, a second-order accurate backward scheme was employed to discretize the second-order derivative of the solid model. The system of discretized equations was solved using a preconditioned Conjugate Gradient method ^44^.

The FSI interface of the coronary artery wall was controlled by the dynamic mesh methodology which was updated according to the solid boundary movements based on Laplace equation. The dynamic mesh methodology was discretized using the cell-centered finite-volume method, and was used to update the computational and geometric nodes of the fluid mesh ^61^. A geometric agglomerated algebraic multi-grid solver was employed to solve the systems of discretized equations.

Finally, the discretized governing equations of the fluid and solid domains were coupled by means of interface Quasi-Newton-Implicit Jacobian Least-Squares (IQN-ILS) algorithm ^73^. It is worth noting that IQN-ILS algorithm has proven to outweigh in stability with better performance in comparison to monolithic method or other partitioned methods such as Aitken’s dynamic relaxation^73,74^.

2.3.8. Analysis of results

**2.3.8.1. Vortical structures**

In order to explore the washout mechanism of flow through vortex morphology, the mid planar velocity streamlines for all the leaflets (left coronary cusp (LCC), right coronary cusp (RCC) and non-coronary cusp (NCC)) is extracted at the peak and late phases of diastole.

**2.3.8.2. Stagnant flow**

The volume of regions with velocity less than 0.001 m/s was obtained for all patients as a measure of blood stasis associated with high risk of thrombus formation.

**2.3.8.3. Time averaged wall shear stress (TAWSS)**

We evaluated the total wall shear stress during diastole using the time-averaged wall shear stress (TAWSS) which is obtained with the formula $TAWSS=\frac{1}{T}\int_{0}^{T} |\tau|dt$ (T and τ are the diastole duration time and instantaneous wall shear stress, respectively). TAWSS was calculated for all patients in both pre and post TAVR states.

**2.3.8.4. Von-mises stress**

The von-Mises stress was obtained using the following equation:

| $\sigma_{VM}= \frac{1}{\sqrt{2}} \sqrt{{(\sigma_{xx}-\sigma_{yy})}^{2}+ {(\sigma_{yy}-\sigma_{zz})}^{2}+{(\sigma_{xx}-\sigma_{zz})}^{2}+6\left( \sigma_{xy}^{2}+\sigma_{yz}^{2}+\sigma_{xz}^{2} \right)^{2}}$ | (32) |
| --- | --- |

## in which *σ_xx_*, *σ_yy_* and *σ_zz_* are the normal stress components and *σ_xy_* _,_ *σ_yz_* and *σ_xz_* are the shear stress components**.**

**Supplementary results**

The hemodynamic results parameters including instantaneous wall shear stress, TAWSS, vortical structure and stagnant flow for the other 4 patients are shown in the following Figures (Figures S1 to S8). In brief, we observed similar trend of significant increase in TAWSS at the aortic root around the PVL location for all patients (Figures S1 and S2). Similar to patients #1 and #2, for the other 4 patients the diverged vortical structure reduced washout power of mainstream flow and lead to increased blood stasis in the neo-sinus regions as shown in Figures S3 to S6 for patients #3 to #6 respectively. With regards to coronary arteries wall shear stress, a universal reduction in WSS was observed for all other 4 patients for both left and right coronary branches after TAVR (Figures s7 and S8), similar to our findings for patients #1 and #2.


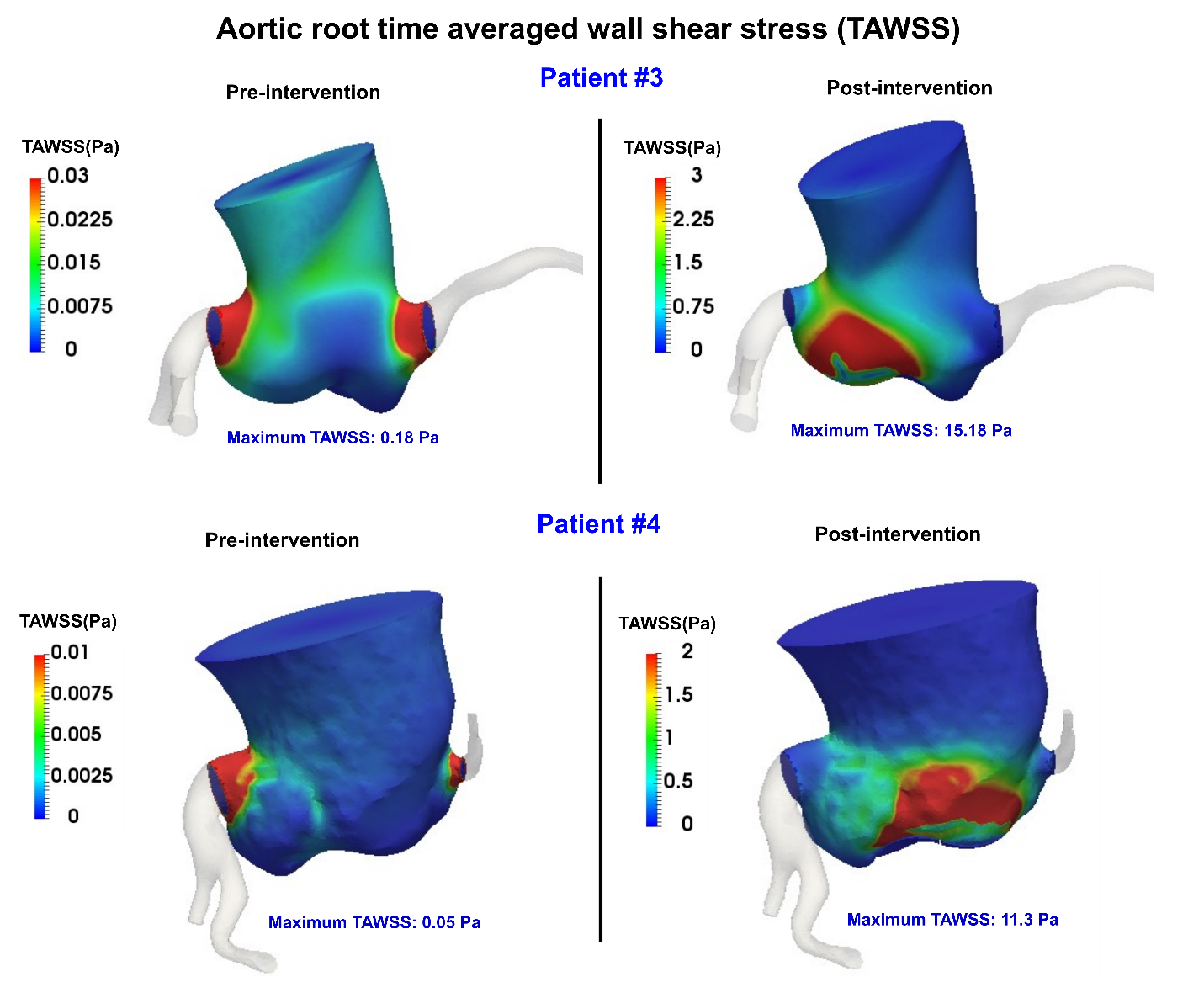


Figure S1. Time averaged wall shear stress of the aortic root during diastole for patients #3 and #4 in both pre and post intervention status


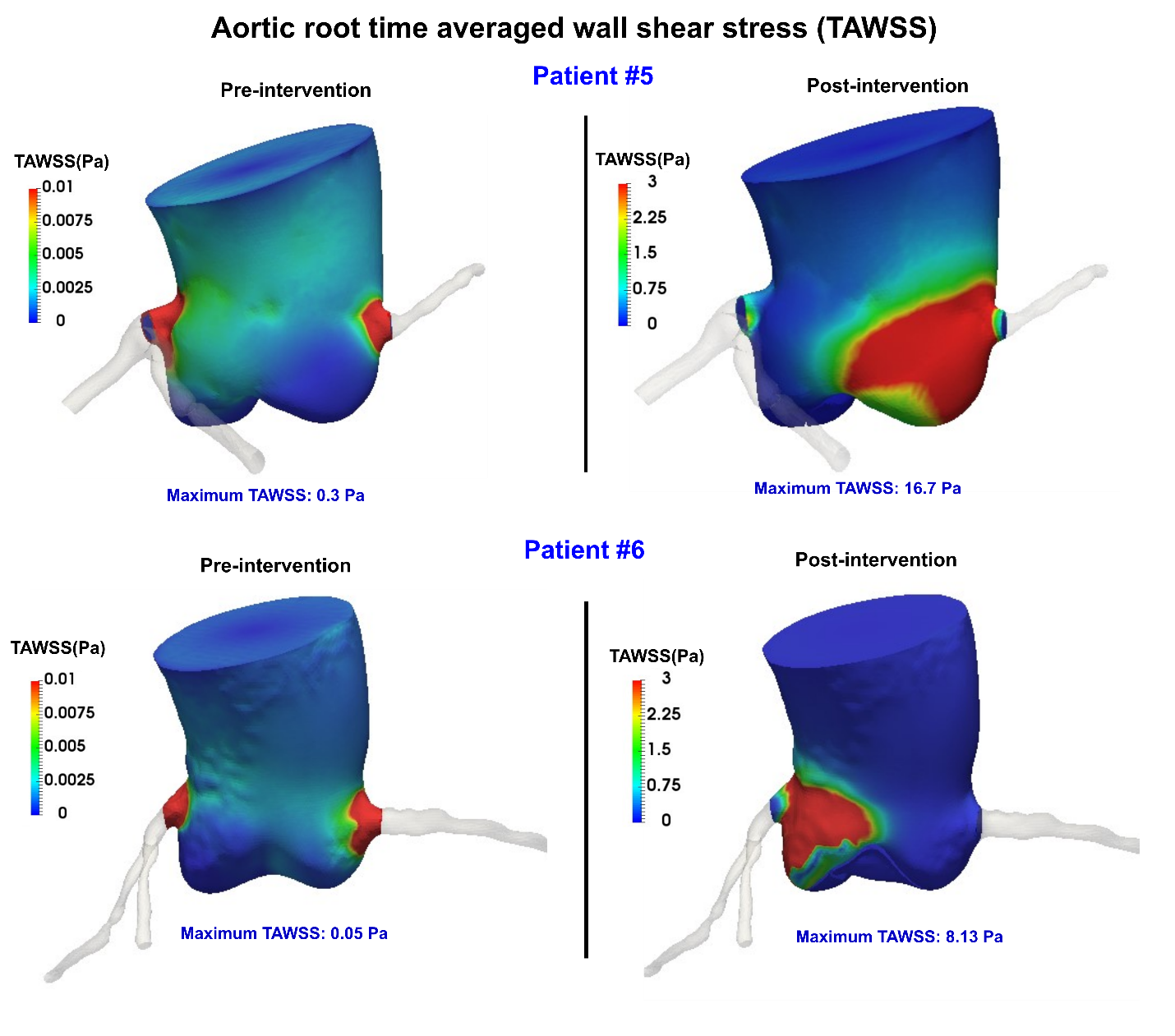


Figure S2. Time averaged wall shear stress of the aortic root during diastole for patients #5 and #6 in both pre and post intervention status


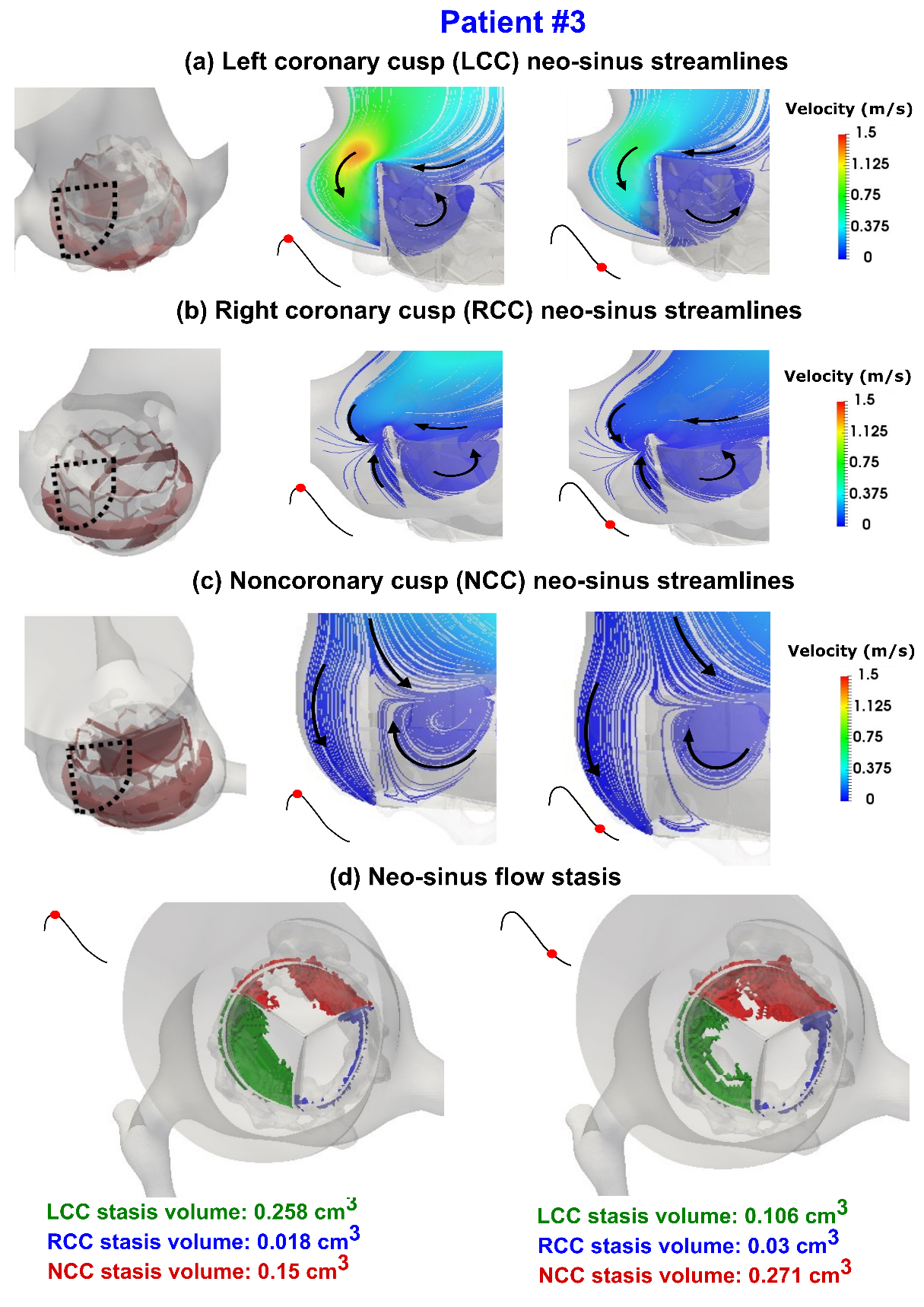


**Figure S3. Evolution of vortical structure following TAVR in the aortic root and neo-sinus regions in patient #3 during diastole**. (a) Mid-planar velocity of left coronary cusp neo-sinus; (b) Mid-planar velocity of right coronary cusp neo-sinus; (c) Mid-planar velocity of non-coronary cusp neo-sinus; (d) Blood stasis volume


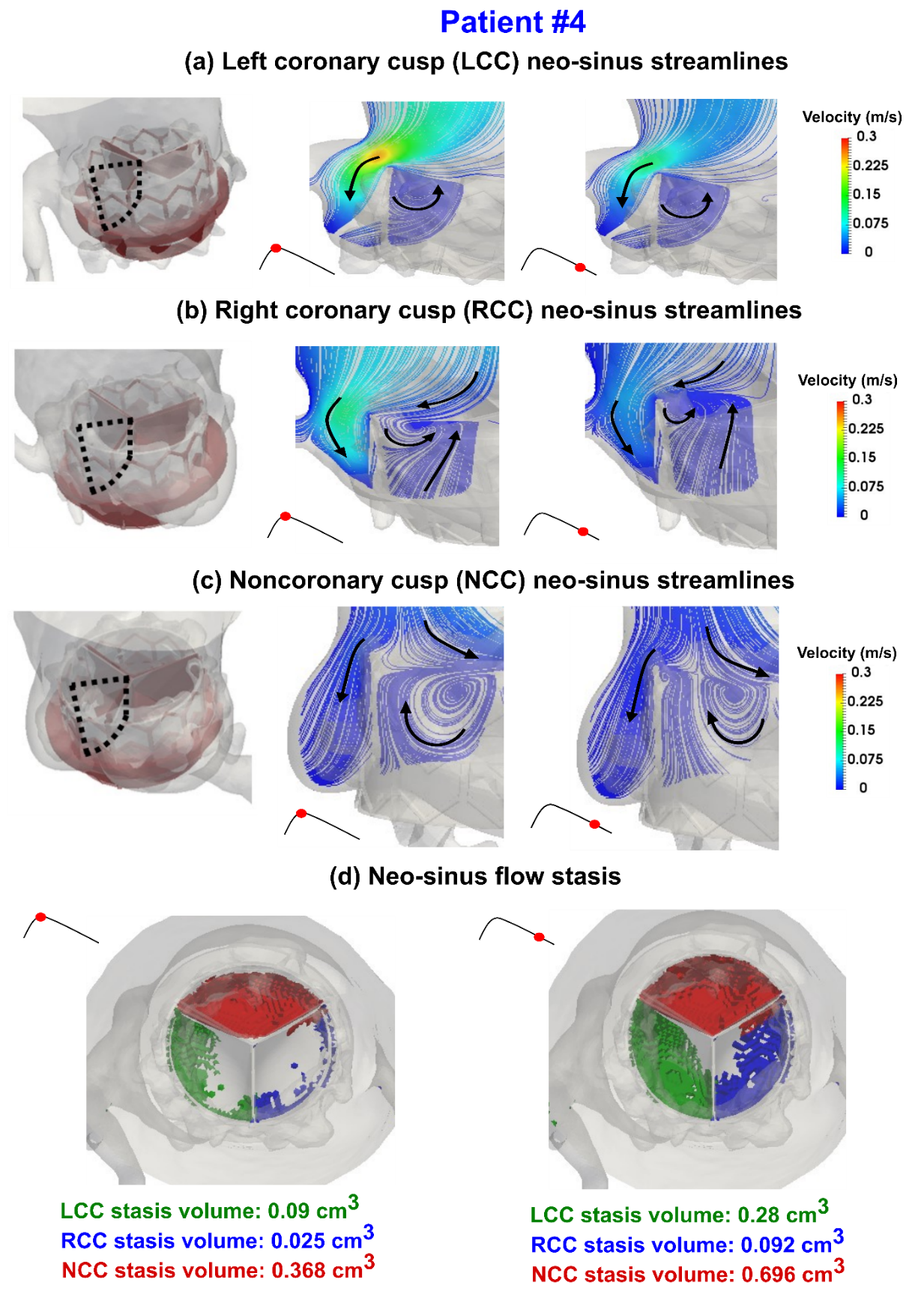


**Figure S4. Evolution of vortical structure following TAVR in the aortic root and neo-sinus regions in patient #4 during diastole**. (a) Mid-planar velocity of left coronary cusp neo-sinus; (b) Mid-planar velocity of right coronary cusp neo-sinus; (c) Mid-planar velocity of non-coronary cusp neo-sinus; (d) Blood stasis volume


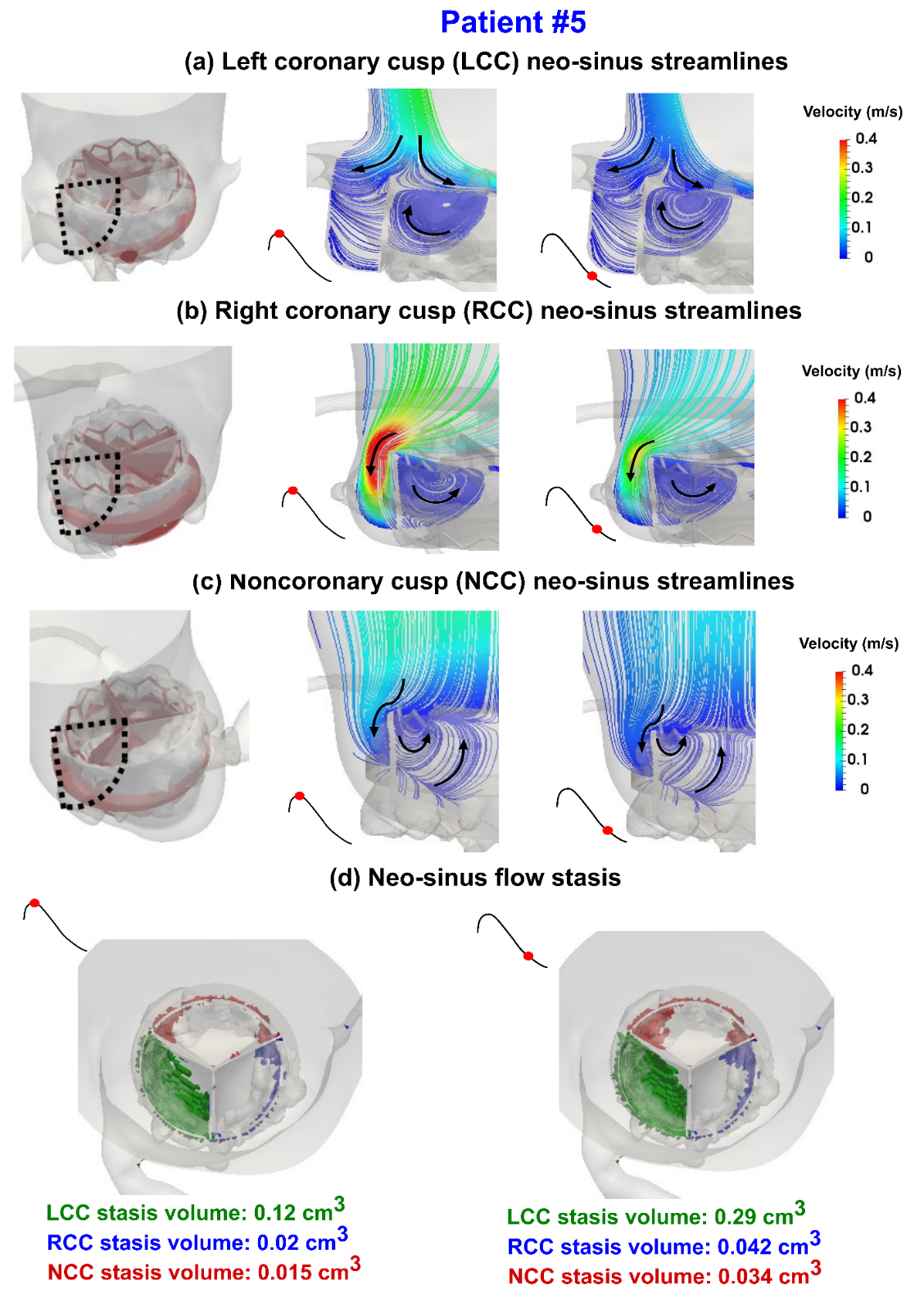


**Figure S5. Evolution of vortical structure following TAVR in the aortic root and neo-sinus regions in patient #5 during diastole**. (a) Mid-planar velocity of left coronary cusp neo-sinus; (b) Mid-planar velocity of right coronary cusp neo-sinus; (c) Mid-planar velocity of non-coronary cusp neo-sinus; (d) Blood stasis volume


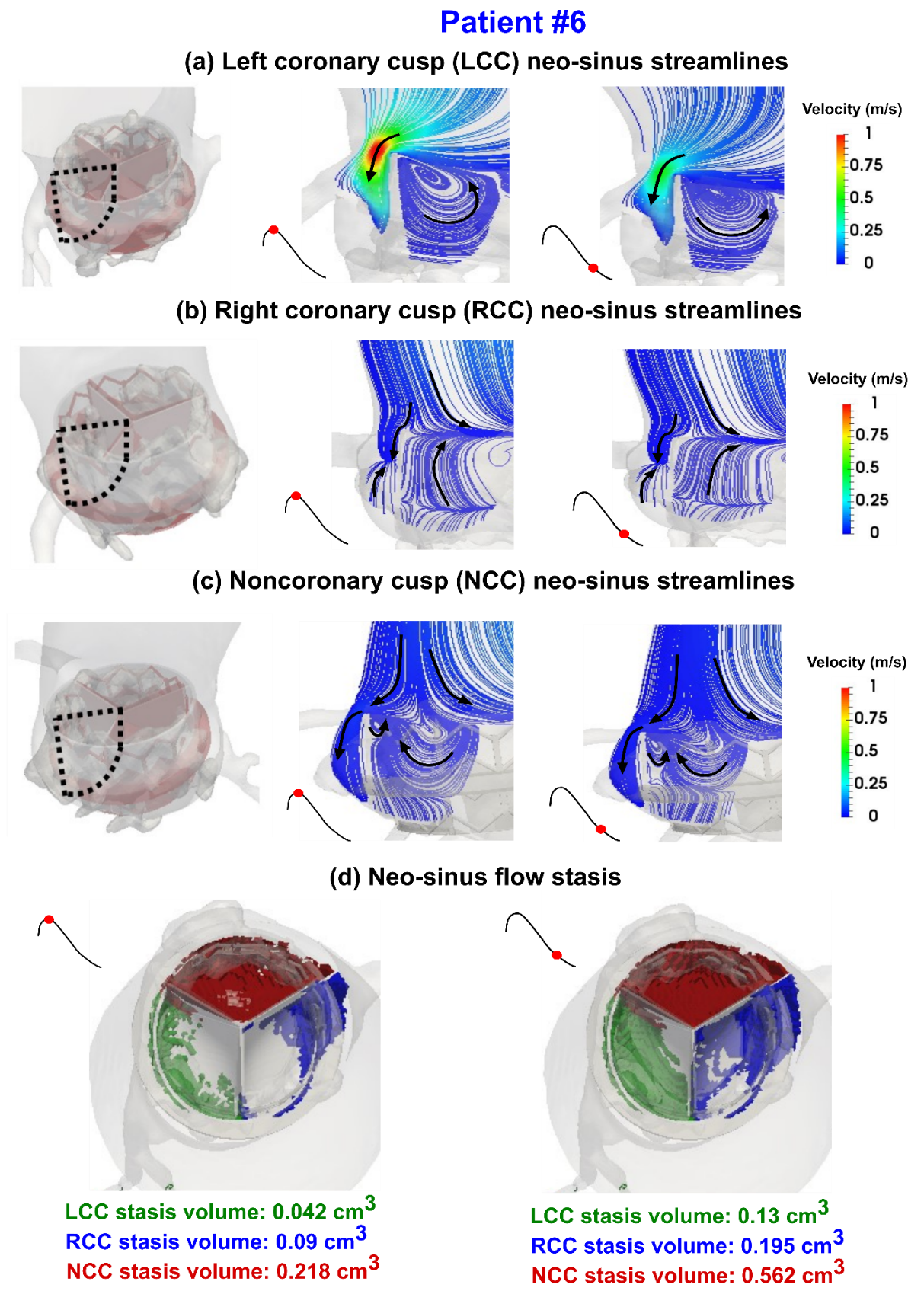


**Figure S6. Evolution of vortical structure following TAVR in the aortic root and neo-sinus regions in patient #6 during diastole**. (a) Mid-planar velocity of left coronary cusp neo-sinus; (b) Mid-planar velocity of right coronary cusp neo-sinus; (c) Mid-planar velocity of non-coronary cusp neo-sinus; (d) Blood stasis volume


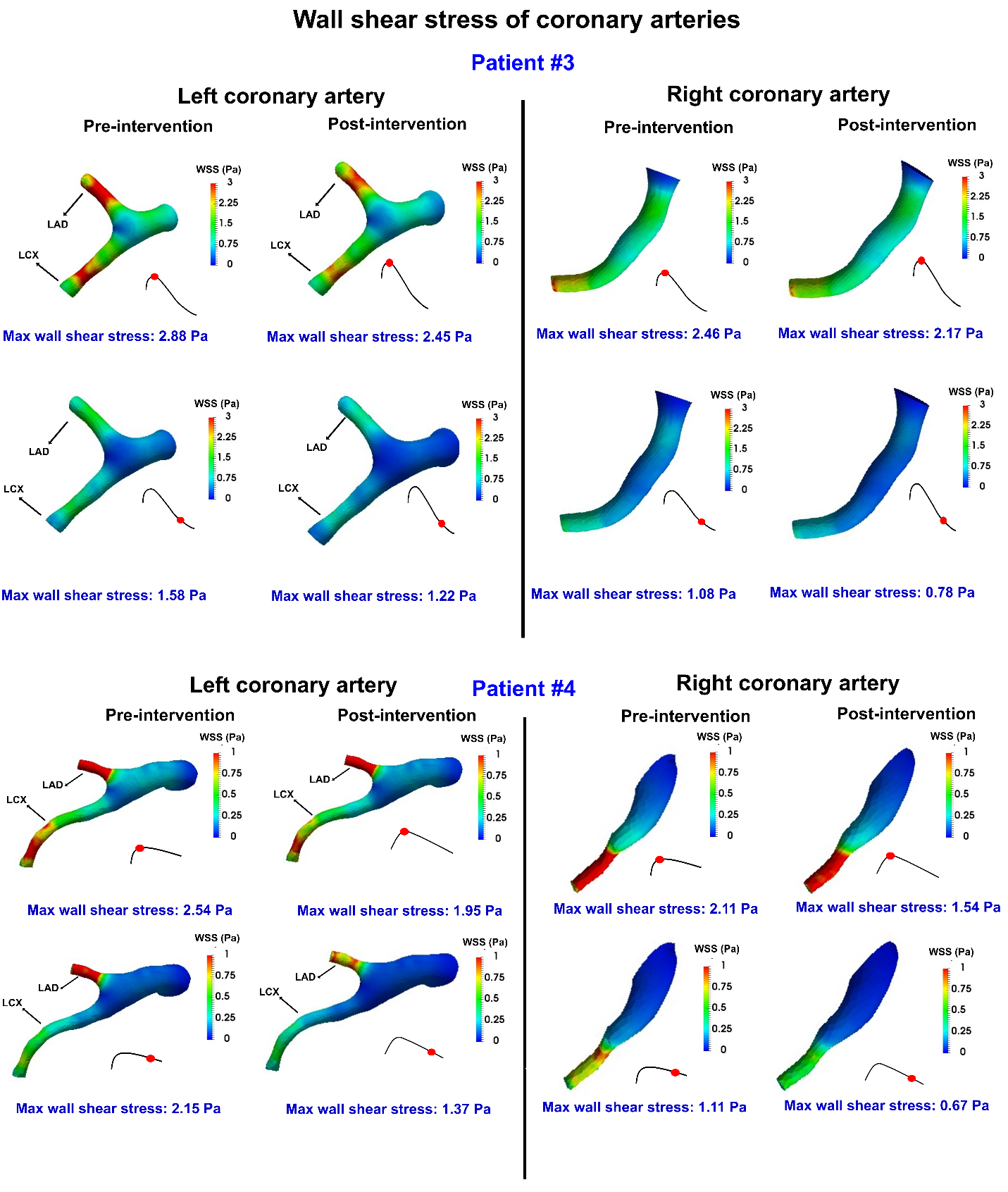


**Figure S7. 3D distribution contours of wall shear stress at peak and end diastole in patient#3 and patient#4 between baseline and 90-day post-TAVR**


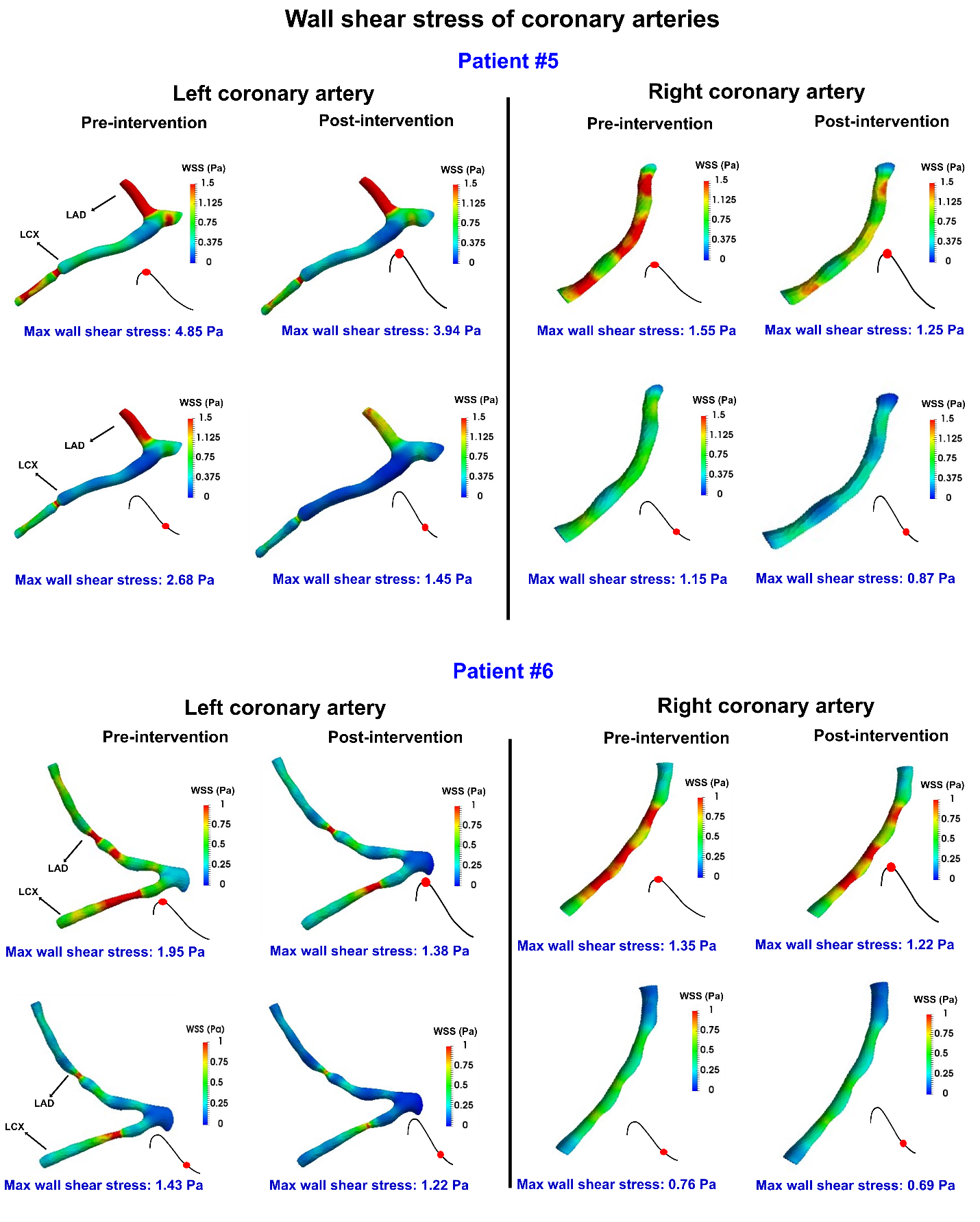


**Figure S8. 3D distribution contours of wall shear stress at peak and end diastole in patient#5 and patient#6 between baseline and 90-day post-TAVR**

**References**

1. Khodaei, S., Fatouraee, N. & Nabaei, M. Numerical simulation of mitral valve prolapse considering the effect of left ventricle. *Math. Biosci.* **285**, 75–80 (2017).

2. Pakravan, H. A., Saidi, M. S. & Firoozabadi, B. A multiscale approach for determining the morphology of endothelial cells at a coronary artery. *Int. J. Numer. Methods Biomed. Eng.* **33**, e2891 (2017).

3. Pandey, R., Kumar, M., Majdoubi, J., Rahimi-Gorji, M. & Srivastav, V. K. A review study on blood in human coronary artery: Numerical approach. *Comput. Methods Programs Biomed.* **187**, 105243 (2020).

4. Jasak, H. & Tuković, Z. *Automatic Mesh Motion for the Unstructured Finite Volume Method*. (2004).

5. Khodaei, S. *et al.* Towards a non-invasive computational diagnostic framework for personalized cardiology of transcatheter aortic valve replacement in interactions with complex valvular, ventricular and vascular disease. *Int. J. Mech. Sci.* **202–203**, 106506 (2021).

6. Khodaei, S. *et al.* Personalized intervention cardiology with transcatheter aortic valve replacement made possible with a non-invasive monitoring and diagnostic framework. *Sci. Rep.* **11**, 10888 (2021).

7. Taki, A., Kermani, A., Ranjbarnavazi, S. M. & Pourmodheji, A. Chapter 4 - Overview of Different Medical Imaging Techniques for the Identification of Coronary Atherosclerotic Plaques. in *Computing and Visualization for Intravascular Imaging and Computer-Assisted Stenting* (eds. Balocco, S., Zuluaga, M. A., Zahnd, G., Lee, S.-L. & Demirci, S.) 79–106 (Academic Press, 2017). doi:10.1016/B978-0-12-811018-8.00004-7.

8. Guo, X. *et al.* Quantify patient-specific coronary material property and its impact on stress/strain calculations using in vivo IVUS data and 3D FSI models: a pilot study. *Biomech. Model. Mechanobiol.* **16**, 333–344 (2017).

9. Kural, M. H. *et al.* Planar biaxial characterization of diseased human coronary and carotid arteries for computational modeling. *J. Biomech.* **45**, 790–798 (2012).

10. Teng, Z., Tang, D., Zheng, J., Woodard, P. K. & Hoffman, A. H. An experimental study on the ultimate strength of the adventitia and media of human atherosclerotic carotid arteries in circumferential and axial directions. *J. Biomech.* **42**, 2535–2539 (2009).

11. Barrett, S. R. H., Sutcliffe, M. P. F., Howarth, S., Li, Z.-Y. & Gillard, J. H. Experimental measurement of the mechanical properties of carotid atherothrombotic plaque fibrous cap. *J. Biomech.* **42**, 1650–1655 (2009).

12. Pandit, A., Lu, X., Wang, C. & Kassab, G. S. Biaxial elastic material properties of porcine coronary media and adventitia. *Am. J. Physiol. Heart Circ. Physiol.* **288**, H2581-2587 (2005).

13. Holzapfel, G. A., Sommer, G. & Regitnig, P. Anisotropic mechanical properties of tissue components in human atherosclerotic plaques. *J. Biomech. Eng.* **126**, 657–665 (2004).

14. Holzapfel, G. A., Stadler, M. & Schulze-Bauer, C. A. J. A Layer-Specific Three-Dimensional Model for the Simulation of Balloon Angioplasty using Magnetic Resonance Imaging and Mechanical Testing. *Ann. Biomed. Eng.* **30**, 753–767 (2002).

15. Karimi, A., Navidbakhsh, M., Shojaei, A. & Faghihi, S. Measurement of the uniaxial mechanical properties of healthy and atherosclerotic human coronary arteries. *Mater. Sci. Eng. C* **33**, 2550–2554 (2013).

16. Carpenter, H. J., Gholipour, A., Ghayesh, M. H., Zander, A. C. & Psaltis, P. J. A review on the biomechanics of coronary arteries. *Int. J. Eng. Sci.* **147**, 103201 (2020).

17. Guo, X. *et al.* Combining IVUS and Optical Coherence Tomography for More Accurate Coronary Cap Thickness Quantification and Stress/Strain Calculations: A Patient-Specific Three-Dimensional Fluid-Structure Interaction Modeling Approach. *J. Biomech. Eng.* **140**, (2018).

18. Wang, L. *et al.* Fluid-structure interaction models based on patient-specific IVUS at baseline and follow-up for prediction of coronary plaque progression by morphological and biomechanical factors: A preliminary study. *J. Biomech.* **68**, 43–50 (2018).

19. Fan, R. *et al.* Human coronary plaque wall thickness correlated positively with flow shear stress and negatively with plaque wall stress: an IVUS-based fluid-structure interaction multi-patient study. *Biomed. Eng. OnLine* **13**, 32 (2014).

20. Gholipour, A., Ghayesh, M. H., Zander, A. C. & Psaltis, P. J. In vivo based biomechanics of right and left coronary arteries. *Int. J. Eng. Sci.* **154**, 103281 (2020).

21. Gholipour, A., Ghayesh, M. H., Zander, A. C. & Psaltis, P. J. In vivo based biomechanics of right and left coronary arteries. *Int. J. Eng. Sci.* **154**, 103281 (2020).

22. Guo, X. *et al.* Quantify patient-specific coronary material property and its impact on stress/strain calculations using in vivo IVUS data and 3D FSI models: a pilot study. *Biomech. Model. Mechanobiol.* **16**, 333–344 (2017).

23. Faroux, L. *et al.* Coronary Artery Disease and Transcatheter Aortic Valve Replacement: JACC State-of-the-Art Review. *J. Am. Coll. Cardiol.* **74**, 362–372 (2019).

24. Chieffo, A. *et al.* Routine Screening of Coronary Artery Disease With Computed Tomographic Coronary Angiography in Place of Invasive Coronary Angiography in Patients Undergoing Transcatheter Aortic Valve Replacement. *Circ. Cardiovasc. Interv.* **8**, e002025 (2015).

25. Ahmad, Y. *et al.* Coronary Hemodynamics in Patients With Severe Aortic Stenosis and Coronary Artery Disease Undergoing Transcatheter Aortic Valve Replacement: Implications for Clinical Indices of Coronary Stenosis Severity. *JACC Cardiovasc. Interv.* **11**, 2019–2031 (2018).

26. Zhong, L. *et al.* Application of Patient-Specific Computational Fluid Dynamics in Coronary and Intra-Cardiac Flow Simulations: Challenges and Opportunities. *Front. Physiol.* **9**, (2018).

27. Pakravan, H. A., Saidi, M. S. & Firoozabadi, B. A multiscale approach for determining the morphology of endothelial cells at a coronary artery. *Int. J. Numer. Methods Biomed. Eng.* **33**, e2891 (2017).

28. Ahmadi, M. & Ansari, R. Computational simulation of an artery narrowed by plaque using 3D FSI method: influence of the plaque angle, non-Newtonian properties of the blood flow and the hyperelastic artery models. *Biomed. Phys. Eng. Express* **5**, 045037 (2019).

29. He, F., Hua, L. & Guo, T. Fluid–structure interaction analysis of hemodynamics in different degrees of stenoses considering microcirculation function. *Adv. Mech. Eng.* **13**, 1687814021989012 (2021).

30. Gholipour, A., Ghayesh, M. H. & Zander, A. Nonlinear biomechanics of bifurcated atherosclerotic coronary arteries. *Int. J. Eng. Sci.* **133**, 60–83 (2018).

31. Rotman, O. M., Zaretsky, U., Shitzer, A. & Einav, S. Pressure drop and arterial compliance – Two arterial parameters in one measurement. *J. Biomech.* **50**, 130–137 (2017).

32. Jahromi, R., Pakravan, H. A., Saidi, M. S. & Firoozabadi, B. Primary stenosis progression versus secondary stenosis formation in the left coronary bifurcation: A mechanical point of view. *Biocybern. Biomed. Eng.* **39**, 188–198 (2019).

33. Gholipour, A., Ghayesh, M. H., Zander, A. & Mahajan, R. Three-dimensional biomechanics of coronary arteries. *Int. J. Eng. Sci.* **130**, 93–114 (2018).

34. Kabinejadian, F. & Ghista, D. N. Compliant model of a coupled sequential coronary arterial bypass graft: Effects of vessel wall elasticity and non-Newtonian rheology on blood flow regime and hemodynamic parameters distribution. *Med. Eng. Phys.* **34**, 860–872 (2012).

35. Nejad, A. A., Talebi, Z., Cheraghali, D., Shahbani-Zahiri, A. & Norouzi, M. Pulsatile flow of non-Newtonian blood fluid inside stenosed arteries: Investigating the effects of viscoelastic and elastic walls, arteriosclerosis, and polycythemia diseases. *Comput. Methods Programs Biomed.* **154**, 109–122 (2018).

36. Gradus-Pizlo, I. *et al.* Left anterior descending coronary artery wall thickness measured by high-frequency transthoracic and epicardial echocardiography includes adventitia. *Am. J. Cardiol.* **91**, 27–32 (2003).

37. Maldonado, N. *et al.* A mechanistic analysis of the role of microcalcifications in atherosclerotic plaque stability: potential implications for plaque rupture. *Am. J. Physiol.-Heart Circ. Physiol.* **303**, H619–H628 (2012).

38. Cardoso, L., Kelly-Arnold, A., Maldonado, N., Laudier, D. & Weinbaum, S. Effect of tissue properties, shape and orientation of microcalcifications on vulnerable cap stability using different hyperelastic constitutive models. *J. Biomech.* **47**, 870–877 (2014).

39. Akyildiz, A. C. *et al.* Effects of intima stiffness and plaque morphology on peak cap stress. *Biomed. Eng. OnLine* **10**, 25 (2011).

40. Maldonado, N., Kelly-Arnold, A., Cardoso, L. & Weinbaum, S. The explosive growth of small voids in vulnerable cap rupture; cavitation and interfacial debonding. *J. Biomech.* **46**, 396–401 (2013).

41. Kohn, J. C., Lampi, M. C. & Reinhart-King, C. A. Age-related vascular stiffening: causes and consequences. *Front. Genet.* **6**, (2015).

42. Morović, S. & Demarin, V. Arterial Stiffness and Aging. in *Mind and Brain: Bridging Neurology and Psychiatry* (ed. Demarin, V.) 129–135 (Springer International Publishing, 2020). doi:10.1007/978-3-030-38606-1_11.

43. Wu Shouling *et al.* Aging, Arterial Stiffness, and Blood Pressure Association in Chinese Adults. *Hypertension* **73**, 893–899 (2019).

44. Tuković, Ž., Karač, A., Cardiff, P., Jasak, H. & Ivanković, A. OpenFOAM Finite Volume Solver for Fluid-Solid Interaction. *Trans. FAMENA* **42**, 1–31 (2018).

45. Cardiff, P. & Demirdžić, I. Thirty years of the finite volume method for solid mechanics. *ArXiv Prepr. ArXiv181002105* (2018).

46. Cardiff, P. *et al.* An open-source finite volume toolbox for solid mechanics and fluid-solid interaction simulations. *ArXiv180810736 Phys.* (2018).

47. Simo, J. C. & Hughes, T. J. R. *Computational Inelasticity*. (Springer Science & Business Media, 2006).

48. Lee, W., Cho, S. W., Allahwala, U. K. & Bhindi, R. Numerical study to identify the effect of fluid presence on the mechanical behavior of the stents during coronary stent expansion. *Comput. Methods Biomech. Biomed. Engin.* **23**, 744–754 (2020).

49. Frattolin, J., Zarandi, M. M., Pagiatakis, C., Bertrand, O. F. & Mongrain, R. Numerical study of stenotic side branch hemodynamics in true bifurcation lesions. *Comput. Biol. Med.* **57**, 130–138 (2015).

50. Rezaeimoghaddam, M. *et al.* Patient-Specific Hemodynamics of New Coronary Artery Bypass Configurations. *Cardiovasc. Eng. Technol.* **11**, 663–678 (2020).

51. Pinto, S. I. S., Romano, E., António, C. C., Sousa, L. C. & Castro, C. F. The impact of non-linear viscoelastic property of blood in right coronary arteries hemodynamics — A numerical implementation. *Int. J. Non-Linear Mech.* **123**, 103477 (2020).

52. Wellnhofer, E. *et al.* Flow simulation studies in coronary arteries—Impact of side-branches. *Atherosclerosis* **213**, 475–481 (2010).

53. Ghorbanniahassankiadeh, A., Marks, D. S. & LaDisa, J. F., Jr. Correlation of Computational Instantaneous Wave-Free Ratio With Fractional Flow Reserve for Intermediate Multivessel Coronary Disease. *J. Biomech. Eng.* **143**, (2021).

54. Tajeddini, F. *et al.* High precision invasive FFR, low-cost invasive iFR, or non-invasive CFR?: optimum assessment of coronary artery stenosis based on the patient-specific computational models. *Int. J. Numer. Methods Biomed. Eng.* **36**, e3382 (2020).

55. Wang, L. *et al.* IVUS-Based FSI Models for Human Coronary Plaque Progression Study: Components, Correlation and Predictive Analysis. *Ann. Biomed. Eng.* **43**, 107–121 (2015).

56. Chen, X., Zhuang, J., Huang, H. & Wu, Y. Fluid–structure interactions ( FSI ) based study of low-density lipoproteins ( LDL ) uptake in the left coronary artery. *Sci. Rep.* **11**, 4803 (2021).

57. Bahrami, S. & Norouzi, M. A numerical study on hemodynamics in the left coronary bifurcation with normal and hypertension conditions. *Biomech. Model. Mechanobiol.* **17**, 1785–1796 (2018).

58. Buradi, A. & Mahalingam, A. Impact of coronary tortuosity on the artery hemodynamics. *Biocybern. Biomed. Eng.* **40**, 126–147 (2020).

59. Eslami, P. *et al.* Validation of Wall Shear Stress Assessment in Non-invasive Coronary CTA versus Invasive Imaging: A Patient-Specific Computational Study. *Ann. Biomed. Eng.* **49**, 1151–1168 (2021).

60. Ribes, A. & Caremoli, C. Salomé platform component model for numerical simulation. in *31st Annual International Computer Software and Applications Conference (COMPSAC 2007)* vol. 2 553–564 (2007).

61. Tuković, Ž. & Jasak, H. A moving mesh finite volume interface tracking method for surface tension dominated interfacial fluid flow. *Comput. Fluids* **55**, 70–84 (2012).

62. Bukač, M., Čanić, S., Tambača, J. & Wang, Y. Fluid–structure interaction between pulsatile blood flow and a curved stented coronary artery on a beating heart: A four stent computational study. *Comput. Methods Appl. Mech. Eng.* **350**, 679–700 (2019).

63. Yushkevich, P. A. *et al.* User-guided 3D active contour segmentation of anatomical structures: significantly improved efficiency and reliability. *Neuroimage* **31**, 1116–1128 (2006).

64. Mao, W., Wang, Q., Kodali, S. & Sun, W. Numerical Parametric Study of Paravalvular Leak Following a Transcatheter Aortic Valve Deployment Into a Patient-Specific Aortic Root. *J. Biomech. Eng.* **140**, 1010071–10100711 (2018).

65. Blanke, P. *et al.* Computed tomography assessment for transcatheter aortic valve in valve implantation: The vancouver approach to predict anatomical risk for coronary obstruction and other considerations. *J. Cardiovasc. Comput. Tomogr.* **10**, 491–499 (2016).

66. Midha, P. A. *et al.* The Fluid Mechanics of Transcatheter Heart Valve Leaflet Thrombosis in the Neosinus. *Circulation* **136**, 1598–1609 (2017).

67. Gorla, R. *et al.* Impact of aortic angle on transcatheter aortic valve implantation outcome with Evolut-R, Portico, and Acurate-NEO. *Catheter. Cardiovasc. Interv.* **97**, E135–E145 (2021).

68. Weller, H. G., Tabor, G., Jasak, H. & Fureby, C. A tensorial approach to computational continuum mechanics using object-oriented techniques. *Comput. Phys.* **12**, 620–631 (1998).

69. Cardiff, P. & Demirdžić, I. Thirty Years of the Finite Volume Method for Solid Mechanics. *Arch. Comput. Methods Eng.* (2021) doi:10.1007/s11831-020-09523-0.

70. Issa, R. I. Solution of the implicitly discretised fluid flow equations by operator-splitting. *J. Comput. Phys.* **62**, 40–65 (1986).

71. Demmel, J. W. Matrix Computations (Gene H. Golub And Charles F. van Loan). *SIAM Rev.* **28**, 252–255 (1986).

72. Oliveira, I. L. [UNESP *et al.* Rupture risk prediction of intracranial aneurysms using open source CFD software. *Int. Symp. Adv. Comput. Heat Transf.* 351–366 (2017).

73. Degroote, J., Bathe, K.-J. & Vierendeels, J. Performance of a new partitioned procedure versus a monolithic procedure in fluid–structure interaction. *Comput. Struct.* **87**, 793–801 (2009).

74. Degroote, J., Bruggeman, P., Haelterman, R. & Vierendeels, J. Stability of a coupling technique for partitioned solvers in FSI applications. *Comput. Struct.* **86**, 2224–2234 (2008).
